# Multiscale mechanistic insights into sonochemical energy coupling and flavor evolution in Pu‑erh tea

**DOI:** 10.1016/j.ultsonch.2025.107735

**Published:** 2026-01-01

**Authors:** Shengjie Duan, Huiqing Luo, Lihui Yu, Jinya Dong, Ziqian Qiao, Shan Liu, Yanan Li, Huajie Yin, Rui Zhou, Yuanfeng Chen, Siyu Zhou, Chen Gong, Yan Shen, Zezhu Du, Li Feng, Xiaocui Du, Jun Sheng, Ruijuan Yang, Chongye Fang

**Affiliations:** a College of Food Science and Technology, Yunnan Agricultural University, Kunming, China; b Yunnan Research Center for Advanced Tea Processing, China; c Key Laboratory of Development and Utilization of Food and Medicinal Resources, Ministry of Education, Yunnan Agricultural University, Kunming 650201, China

**Keywords:** Sonochemical energy coupling, Acoustic cavitation, Pu‑erh tea, Molecular dynamics, Multiscale mechanism, *Energy–structure–function* relationship

## Abstract

Pu’erh tea (*Camellia sinensis* var. *assamica*) represents a highly complex multiphase fermentation system in which flavor formation spans chemical transformation, energy transfer and microecological succession. To elucidate the mechanistic basis by which sonochemical energy input accelerates flavor evolution, we developed an integrated multiscale model combining flavoromics, molecular dynamics simulations and microbial ecological analysis. The model captures molecular reactions and metabolic regulation under ultrasonic cavitation.Increasing acoustic power density (0.3–0.8  W mL^−1^) substantially enhanced cavitation intensity and energy absorption, accompanied by elevated concentrations of reactive radicals (•OH 40–96  µM) and an increased mass-transfer coefficient, generating high-energy heterogeneous microdomains. Sonochemical coupling reduced the reaction barrier of ester-type catechins (ΔG ≈ –25  kJ mol^−1^) and accelerated their conversion into free acid polyphenols. Concurrently, high shear forces induced partial depolymerization of proteins and peptides, leading to 1.5–3-fold increases in taste-active amino acids and soluble sugars, thus reinforcing the “*mellow*” and “*sweet*” mouthfeel of the infusion.Microecological (*meta*-omics) profiling revealed that elevated acoustic energy favored the enrichment of functional microbial consortia dominated by *Lactobacillus plantarum* and *Aspergillus niger*. Pathways related to aroma synthesis—including phenylalanine metabolism and monoterpene biosynthesis—exhibited approximately twofold enrichment, driving the accumulation of aromatic esters and terpenes. Multivariate modeling (PLSR and RDA, R^2^ > 0.90, Q^2^ > 0.70) confirmed that acoustic power and cavitation indices quantitatively predict flavor outputs. However, rigorous techno-economic analysis and acoustic propagation modeling in solid-state media reveal that industrial scaling faces challenges regarding energy consumption, reactor design for solid–liquid mixtures, and downstream dewatering costs. While the optimal window of 0.6–0.75 W·mL^−1^ reproducibly generated complex aromatic profiles comparable to aged tea, these engineering constraints necessitate further optimization for commercial viability. Collectively, these findings elucidate the cross-scale mechanism by which coupled sonochemical energy drives flavor evolution and define the application boundaries for green, energy-precise processing of fermented beverages.

## Graphical abstract

**Figure:**
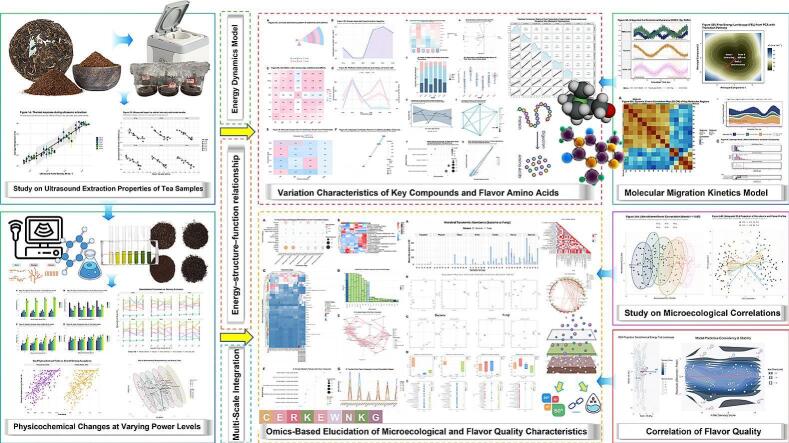

## 1 Introduction

Pu’erh tea [1] (*Camellia sinensis* var. *assamica*) is a distinctive post‑fermented tea originating from Yunnan, China, and serves as an important natural model system for studying complex fermentation processes [2]. Its multilayered aged aroma and mellow taste arise not from a single metabolic pathway but from long‑term, synergistic chemical and biological evolution. Polyphenols, amino acids and carbohydrates in the tea matrix undergo oxidative polymerization, decarboxylation, esterification and Maillard‑type reactions during storage, producing a wide spectrum of taste‑ and aroma‑active molecules [3]. Concurrently, composite microbial consortia dominated by *Aspergillus*, lactic acid bacteria and yeasts [4] dynamically evolve, reconstructing metabolic networks and driving the biosynthesis of complex pigments and volatiles. The aging process extends from months to decades, encompassing chemical reactions, mass transfer and microecological metabolism at intertwined temporal and spatial scales [5]. Distinct process conditions and aging durations yield pronounced flavor diversification [6], yet the fundamental relationships between energy input and structural evolution remain largely unquantified.

Previous understanding of Pu’erh tea flavor development has focused on component identification and sensory description, with limited insight into the underlying energetic and kinetic processes. A key unresolved question in tea science and in broader food‑fermentation research concerns how microbial‑chemical reaction networks acquire and dissipate energy within multiphase systems [7]. The prolonged timescale, complex reaction pathways and limited controllability of aging environments [8] hinder real‑time tracking of energy redistribution [9] and kinetic variation [10]. Establishing a model system with tunable energy input, quantifiable reaction parameters and observable molecular behavior is therefore essential for uncovering the physical basis of flavor formation.

Ultrasound technology, widely deployed in materials science and biochemical engineering [11], enhances mass transfer and reaction activation through sonochemical effects. Propagation of acoustic waves in liquids produces alternating high‑ and low‑pressure cycles, leading to the formation and asymmetric collapse of cavitation bubbles [12]. These microsecond events generate extreme local conditions—temperatures up to ∼5000  K, pressures of several hundred atm and cooling rates exceeding 10^9^ K s^−1^—accompanied by high‑velocity microjets (>100  m s^−1^) and reactive radical species (•OH, •H, ROO•) [13]. Such transient microenvironments steeply modify potential‑energy surfaces and accelerate heterogeneous‑phase reactions [14]. Consequently, sonochemistry offers a powerful approach for precise control of reaction kinetics and selectivity. Cavitation intensity and spatial distribution depend on acoustic frequency, power density, medium viscosity and solid‑phase concentration [15], making plant‑derived multiphase systems with complex interfaces ideal platforms for mechanistic study.

Unlike homogeneous liquids, tea‑extract matrices feature high solid content, organic substrates and active microorganisms, yielding highly nonuniform energy distribution [16]. Beyond enhancing mass transfer, acoustic energy may reshape polyphenol oxidation–polymerization and aroma‑release pathways through localized radical formation, microjet impact and high‑shear effects [17]. Recent studies demonstrate that ultrasound‑assisted extraction (UAE) significantly improves the recovery of tea polyphenols, catechins, free amino acids and volatile compounds [18], enhancing sensory quality. However, most reports remain phenomenological and lack quantitative mechanistic insight into the relationships among acoustic power, reaction rate and molecular transformation [19]. Whether sonochemical energy conversion and reaction dynamics in bio‑rich natural multiphase systems [20] adhere to classical kinetic models represents a frontier challenge in sonochemistry. The Pu’erh tea system—integrating concurrent chemical reactions, microbial metabolism and multilevel interfaces—provides a natural experimental platform to probe energy‑coupling mechanisms in complex fermentations.

To address these gaps, we developed a multiscale, multimodule framework to systematically characterize how ultrasonic energy input modulates flavor formation across fermentation processes and aging stages of Pu’erh tea. The study focused on three central questions: (i) How does acoustic energy reshape molecular reaction kinetics and potential‑energy landscapes in multiphase systems [21]? (ii) Is there quantifiable coupling between cavitation characteristics and microecological metabolic pathways under varying power densities?(iii) Can a unified energy–structure–function model be established to predict flavor outcomes based on sound‑field strength?Our experimental strategy comprised three tiers. First, at the reaction scale, a quantitative model was established linking acoustic power parameters to chemical‑reaction kinetics [22]. Controlled ultrasound treatments (20–60  kHz, 0.3–0.8  W mL^−1^, variable exposure times) were applied to monitor temporal changes in amino acids, phenolics, organic acids and volatile precursors, enabling calculation of apparent rate constants and activation energies to define functional correlations between energy input and reaction efficiency. Second, at the microecological scale, we explored ultrasound‑induced alterations in community composition and function [23]. High‑throughput sequencing of 16S rRNA and ITS regions was used to analyze the response of microbial diversity, metabolic pathways and endogenous enzyme systems to acoustic perturbation [24], revealing mechanisms of “*sonically induced metabolic regulation.*” Third, at the molecular scale, molecular‑dynamics (MD) and density‑functional‑theory (DFT) approaches were employed to construct cavitation‑microzone models [25]. Representative flavor precursors—including epigallocatechin gallate, tea polyphenols and alkaloids—were subjected to reaction‑pathway scanning under radical attack, enabling calculation of energy surfaces and bond‑energy variations [26] to elucidate molecular rearrangement mechanisms under sonochemical excitation.

Building on these designs, we hypothesize that ultrasound energy, through transient high‑temperature, high‑pressure and shear‑stress release in cavitation microdomains, redistributes system‑wide energy and dynamically modulates kinetic parameters [27]. This process restructures both chemical reaction networks and microbial metabolic circuits, ultimately driving directional formation and release of key flavor compounds. Conceptually, sonochemical modulation represents a multiscale energy‑coupling phenomenon—its impact extends beyond mass transfer and interfacial disruption to include micro‑level control of energy states and self‑organization within complex systems [28]. (i) Mechanistic level – To reveal molecular pathways of sonochemical energy participation in heterogeneous fermentation, identify radical‑mediated oxidation and polymerization steps of aroma precursors, and delineate how acoustic‑field‑induced microenvironmental changes influence microbial metabolism. (ii) Methodological level – To establish a multiscale coupling model linking ultrasonic power, reaction rate and flavor output, integrating UAE experimentation, flavoromics, microbial‑network analysis and molecular‑simulation data for quantitative cooperative characterization [29]. (ii) Applied level – To define characteristic energy domains across power ranges and propose controllable acoustic‑energy strategies for tea and other plant‑based fermentations, enabling low‑energy, precision flavor engineering and providing a theoretical basis for green processing and intelligent sonic manufacturing.

Although the tea‑powder–water multiphase system constructed in this study enables precise delivery and monitoring of sonochemical energy, it must be emphasized that its spatial architecture, mass‑transfer pathways, and microenvironmental heterogeneity differ fundamentally from those of authentic piled fermentation of Pu‑erh tea. Real fermentation matrices exhibit solid‑state porosity, moisture gradients, and intricate gas–liquid–solid interfaces, all of which may influence acoustic field distribution and energy attenuation. Consequently, the kinetic models and energy‑coupling mechanisms established here are intended to elucidate physicochemical principles at the microscopic level rather than serve as direct analogues for industrial solid‑state fermentation. Future work will extend this mechanistic framework to evaluate its applicability within whole‑leaf piling systems.

## 2 Materials and methods

### 2.1 Materials

All Pu’erh tea samples used in this study were supplied by Yunnan Pu’erh Tea Factory Co., Ltd. (Pu’er City, Yunnan, China), and originated from the Yunnan large‑leaf cultivar *Camellia sinensis* var. *assamica*. To construct representative systems covering different fermentation processes and aging stages, six characteristic teas were selected, encompassing both naturally aged “raw” teas and pile‑fermented “ripe” teas. Specifically: 2004 Qing‑fa round tea (naturally dried and fermented), 2004 dry‑storage tea (post‑fermented under low humidity), 2004 fermentation‑storage tea (wet‑stored system), 2008 ripe‑yun fermentation‑storage tea (mid‑aged ripe tea), 2014 ripe‑xiang fermentation‑storage tea (accelerated ripe‑tea system) and 2019 ripe‑cheng fermentation‑storage tea (new‑period ripe tea). All batches were authenticated by the manufacturer and processed from tea leaves harvested in the same production region. Fermentation designs were conducted under controlled factory conditions, with raw teas undergoing natural oxidative aging, and ripe teas produced through pile fermentation (50–80 °C, relative humidity  60–80 %), forming complex microecological fermentation systems.

Upon receipt, all samples were sealed and stored under light‑proof dry conditions (ambient temperature 20 ± 2 °C, relative humidity < 60 %) to maintain original physicochemical attributes [30]. Prior to experiments, samples were ground with an analytical mill (IKA A 11 Basic, Germany) to an average particle size of ∼0.45  mm, sifted through a 40‑mesh screen and homogenized. Powdered samples were packed in high‑density polyethylene bags and stored at room temperature for no longer than  2  weeks before ultrasonic‑assisted extraction (UAE) experiments [31]. Each sample was weighed precisely to ensure consistent solid‑to‑liquid ratios and enhance reproducibility.

Solvents and standards used in this work included methanol, acetonitrile, formic acid, taste‑active amino acids, organic acids, tea polyphenols and catechin reference substances, all purchased from Sigma‑Aldrich (St. Louis, MO, USA) and Fisher Scientific (Waltham, MA, USA), analytical‑grade. Ultrapure water was produced by a Milli‑Q system (resistivity > 18.2 MΩ·cm). These reagents were employed for subsequent flavoromics quantification (LC‑MS/MS), physicochemical analysis and preparation of samples for microbial community sequencing.

For comparability and modeling purposes, the six teas were coded according to production year and process type as follows:PT‑G = 2004 Qing‑fa round tea; PT‑D = 2004 dry‑storage tea; PT‑F = 2004 fermentation‑storage tea; PT‑R = 2008 ripe‑yun fermentation‑storage tea; PT‑C = 2014 ripe‑xiang fermentation‑storage tea; and PT‑A = 2019 ripe‑cheng fermentation‑storage tea. Their distinct processing methods, aging durations and storage conditions provide an ideal set of model systems for decoding sonochemical reaction mechanisms and flavor‑quality formation in Pu’erh tea.

### 2.2 Ultrasound‑assisted extraction procedure

Ultrasound‑assisted extraction (UAE) experiments were carried out using a thermostatic sonochemical reactor. A probe‑type ultrasonic processor (VCX‑500, Sonics & Materials Inc., USA) equipped with a titanium‑alloy horn (diameter 13  mm, operating frequency 20  kHz, maximum output 500  W [32]) was employed. Based on equipment calibration, actual acoustic power was measured with a wattmeter, and the power density (P/V, W mL^−1^) was calculated according to solution volume, with a conversion efficiency of approximately 82 %. The standard acoustic power density was set at 0.4  W mL^−1^ (40 % of full power), with comparative trials conducted at 0.3, 0.6 and 0.8  W mL^−1^ [33] to evaluate the influence of energy input on reaction behavior.

For each run, 5.00 ± 0.01  g of tea powder was mixed with 100  mL of deionized water (liquid‑to‑solid ratio = 20  mL g^−1^) in a 250  mL ultrasound‑resistant glass vessel [34]. A pulsed mode (5  s on/5 s off) was used to prevent local overheating [35]. The reaction temperature was maintained at 25 ± 1 °C using an external circulating chiller, with temperature rise rate and peak value continuously recorded to estimate acoustic energy absorption. Solution viscosity and suspended‑particle size were measured before and after sonication to quantify acoustic‑field propagation differences. Cavitation intensity was determined by the potassium iodide (KI) dosimetry method [36] and further validated by sonochemiluminescence analysis [37] to characterize cavitation activity. Variations in power and sound‑intensity curves were monitored using a power‑detection system (UP400ST, Hielscher Ultrasonics GmbH, Germany), from which cavitation indices and energy‑distribution parameters were derived.Following 30  min of ultrasonic treatment, the reaction mixture was immediately cooled to room temperature and filtered through 0.45  μm PTFE membranes. The filtrates were collected for subsequent flavoromics profiling, physicochemical characterization and microecological analysis. A control group (CK) without ultrasound was processed under identical conditions of liquid‑to‑solid ratio, stirring duration (30  min) and temperature to distinguish pure extraction effects from genuine sonochemical reactions. All experiments were performed in triplicate, and results are reported as mean ± standard deviation. During sonication, pH, dissolved‑oxygen concentration and temperature were continuously monitored to assess the influence of acoustic energy on the chemical environment and redox state [38]. All filtrates were refrigerated at 4 °C and analyzed within 24  h. With these controlled and calibrated parameters, the UAE experiments ensured reproducible sonochemical conditions and cross‑system comparability, providing a robust dataset for subsequent analyses of energy coupling and flavor‑formation mechanisms.

### 2.3 Physicochemical and flavoromics analyses

#### 2.3.1 Flavoromics analysis

Flavoromics profiling was conducted in collaboration with Shanghai Biomarker Technologies Co., Ltd. (Shanghai, China). Sample pretreatment [39] included centrifugation of the tea infusions (12 000 r min^−1^, 10  min), after which 1  mL of supernatant was collected, vacuum‑concentrated, redissolved in 70 % methanol, and filtered through 0.22  μm membranes prior to analysis.

Measurements were performed on an ultra‑high‑performance liquid chromatography–high‑resolution mass spectrometry system (UHPLC–MS/MS; Q Exactive Plus, Thermo Fisher Scientific, USA). Separation was achieved using a Hypersil GOLD C18 column (100  mm × 2.1  mm, 1.9  μm). The mobile phase consisted of (A) water with 0.1 % formic acid and (B) acetonitrile with 0.1 % formic acid; flow rate 0.3  mL min^−1^; column temperature 40 °C. The mass spectrometer operated in both positive and negative ion modes across a full scan range of *m*/*z* 70 – 1000 at 70 000 FWHM resolution (at *m*/*z* 200) [40]. Raw data were processed using Compound Discoverer 3.1 software for peak detection, retention‑time alignment, and quantification. Metabolite structures were annotated using an in‑house library together with HMDB, MassBank and mzCloud databases. Data matrices were Pareto‑standardized before statistical analysis, including principal component analysis (PCA), hierarchical clustering and correlation analysis with physicochemical indices.

#### 2.3.2 Determination of taste‑active amino acids

Free amino acids in tea infusions were quantified using an automatic amino acid analyzer (L‑8900, Hitachi, Japan) [41]. After filtration through 0.22  μm membranes, samples were diluted with 0.02  mol L^−1^ lithium citrate buffer before injection. Chromatographic separation was carried out at 57 °C with a flow rate of 0.4  mL min^−1^, and detection was performed colorimetrically by the ninhydrin method [42] at 570  nm and 440  nm. Standards were obtained from Sigma‑Aldrich. The results were used to calculate total amino acid content and the relative proportions of principal taste‑active amino acids.

#### 2.3.3 Determination of major chemical constituents

Key active constituents examined included gallic acid (GA), epigallocatechin (EGC), catechin (C), caffeine (CAF), epicatechin (EC), epigallocatechin gallate (EGCG) and epicatechin gallate (ECG). Analyses were performed on an Agilent 1260 HPLC system equipped with a ZORBAX SB‑C18 column (250  mm × 4.6  mm, 5  μm). The detection wavelength was 280  nm. The mobile phase consisted of (A) 0.1 % formic‑acid aqueous solution and (B) acetonitrile, using a gradient program as follows: 0–10  min, 8 %–15 %B; 10–25  min, 15 %–25 %B; 25–30  min, 25 %–8%B. The column temperature was 30 °C, and flow rate 1.0  mL min^−1^. Series of standard solutions were used to construct calibration curves with correlation coefficients R^2^ > 0.999 [43].

#### 2.3.4 Basic physicochemical indices

Moisture content was determined by the hot‑air drying method (GB/T 8304‑2013) [44]. Total tea polyphenols were measured following the Folin‑Ciocalteu colorimetric assay [45] (GB/T 8313‑2018) using gallic acid as the calibration standard. Total sugars were quantified by the phenol–sulfuric acid method [46]; water‑extractable matter was determined according to GB/T 8305‑2013 [47]; and tea polysaccharides were analyzed by the anthrone–sulfuric colorimetry [48]. All data are expressed on an air‑dry basis (% w/w) to evaluate the influence of acoustic‑field treatment on physicochemical composition.

#### 2.3.5 Microecological community structure analysis

Microbiome analysis was also conducted by Shanghai Biomarker Technologies Co., Ltd. Tea suspensions before and after sonochemical treatment were centrifuged, and the pellets used for total DNA extraction with the FastDNA™ Spin Kit for Soil (MP Biomedicals, USA). The bacterial 16S rRNA gene (V3–V4 region) was amplified with primers 338F/806R, and the fungal ITS region with ITS1F/ITS2R [49]. Amplicons were sequenced on the Illumina NovaSeq 6000 platform to generate paired‑end reads (PE 250  bp). Raw sequences were processed in QIIME 2 (v2022.8) for quality filtering, dereplication and feature clustering, and taxonomic assignment was performed using SILVA 138 and UNITE databases [50]. All samples were rarefied to 100 000 reads prior to computing α‑diversity (Chao1 and Shannon indices) and β‑diversity (Bray‑Curtis distance). Differential taxa were identified by LEfSe analysis. Functional metagenome prediction was conducted using PICRUSt2 to infer potential metabolic alterations under acoustic‑energy intervention.

It is important to underscore that PICRUSt2 infers functional gene abundance from phylogenetic relatedness, thereby reflecting only the *potential metabolic capacity* of the microbial community rather than its actual transcriptional activity or enzymatic reaction rates. Accordingly, the predicted functional profiles should be regarded as indicative trends, which must be corroborated in subsequent studies through metatranscriptomic or targeted metabolomic analyses.

#### 2.3.6 Sensory evaluation

Sensory evaluation followed the GB/T 23776‑2018 protocol. Nine certified national‑level tea assessors formed the expert panel, scoring samples for aroma, taste, liquor color, leaf appearance, and overall acceptability [51]. Samples were coded and presented in randomized order for blind testing. A 9‑point hedonic scale was applied (9 = excellent, 1 = poor). Each sample was evaluated in triplicate, and the mean value was taken as its composite sensory score.

All analytical results were subsequently integrated with acoustic‑energy parameters—including power density, cavitation intensity, and energy‑absorption ratio—for multivariate modeling of sonochemistry‑flavor‑microecology interactions.

### 2.4 Multidimensional data integration and statistical analysis

#### 2.4.1 Univariate differential analysis

Physicochemical parameters and chemical constituents were analyzed by one‑way ANOVA using Origin 2022 (OriginLab, USA). Statistical significance was evaluated at a two‑tailed *p* < 0.05, and outliers were excluded based on the Grubbs test. Differences in flavor‑metabolite abundance were computed on the MetaboAnalyst 5.0 platform by calculating Fold Change (FC) and p‑values (threshold |log_2_ FC| ≥ 1 and *p* < 0.05 after FDR correction). Differential metabolites were visualized by principal‑component analysis (PCA) and partial‑least‑squares discriminant analysis (PLS‑DA). Models were validated via seven‑fold cross‑validation and permutation testing (*n* = 200), with *R^2^* > 0.7 and *Q^2^* > 0.5 considered acceptable predictive standards.

#### 2.4.2 Microecological data analysis

16S rRNA and ITS amplicon sequences were processed in QIIME 2 (v2022.8) to calculate α‑diversity (Chao1 and Shannon indices) and β‑diversity (Bray‑Curtis and UniFrac distances). Principal‑coordinate analysis (PCoA) and non‑metric multidimensional scaling (NMDS) were performed using “vegan” and “microbiome” packages in R. Correlations between microbial community structures and acoustic‑power density were assessed by Mantel tests (Pearson, *p* < 0.05). Functional prediction was conducted on the PICRUSt2 platform to generate KEGG pathway abundance matrices and identify metabolic functions affected by sonochemical energy input.

#### 2.4.3 Multi‑omics integration analysis

To elucidate the synergistic coupling between sonochemical energy input, flavor chemistry, and microecological response, canonical‑correspondence analysis (CCA) and redundancy analysis (RDA) were used to construct association models between physicochemical parameters and community composition [52]. Correlations between flavor metabolites and indicator taxa were determined by Spearman coefficients (|ρ| > 0.7, *p* < 0.05 after Bonferroni correction), and co‑occurrence networks were visualized in Cytoscape (v3.9.1). Modular analysis using the Girvan–Newman algorithm was applied to identify cross‑dimensional co‑regulatory clusters. Integrated datasets were further modeled using multiple‑block PLS (mixOmics R package v6.22) for multi‑omics fusion; key variables were selected using *VIP* > 1, and model significance was verified through permutation testing.

#### 2.4.4 Model performance evaluation and visualization

Comprehensive correlation matrices were constructed in R (v4.2.3) and Python (v3.10). Visualization outputs included heatmaps, volcano plots, hierarchical trees, and three‑dimensional acoustic‑power–reaction–flavor distributions. Partial‑least‑squares regression (PLSR) was applied to quantify the relationships between energy input and key reaction‑rate constants, with model performance evaluated by *R^2^*, *Q^2^* and *RMSEE*. A multidimensional correlation matrix was ultimately generated to identify continuous coupling between cavitation effects and the integrated flavor–microecology evolution.

All statistical analyses adopted two‑tailed tests at *p* < 0.05 after false‑discovery‑rate (FDR) correction. The multivariate statistical and modeling framework established here was designed to quantitatively reveal how ultrasonic energy drives molecular transformations and cooperative microecological dynamics in complex fermentation systems, providing a rigorous basis for cross‑scale mechanistic interpretation of sonochemical processes.

## 3 Results and discussion

### 3.1 Acoustic characterization and cavitation dynamics during ultrasonic extraction

To elucidate the physical mechanisms of ultrasonic energy input in the Pu’erh tea extraction system, we quantitatively characterized the acoustic energy distribution [53], temperature dynamics, cavitation activity, and reactive radical production under different acoustic power densities (0.3, 0.4, 0.6 and 0.8  W·mL^−1^). Experiments were conducted using the 20  kHz probe–type ultrasound setup under a pulsed mode of 5  s on / 5  s off, effectively suppressing heat accumulation and ensuring the dominance of sonochemical effects.

**Fig. 1:**
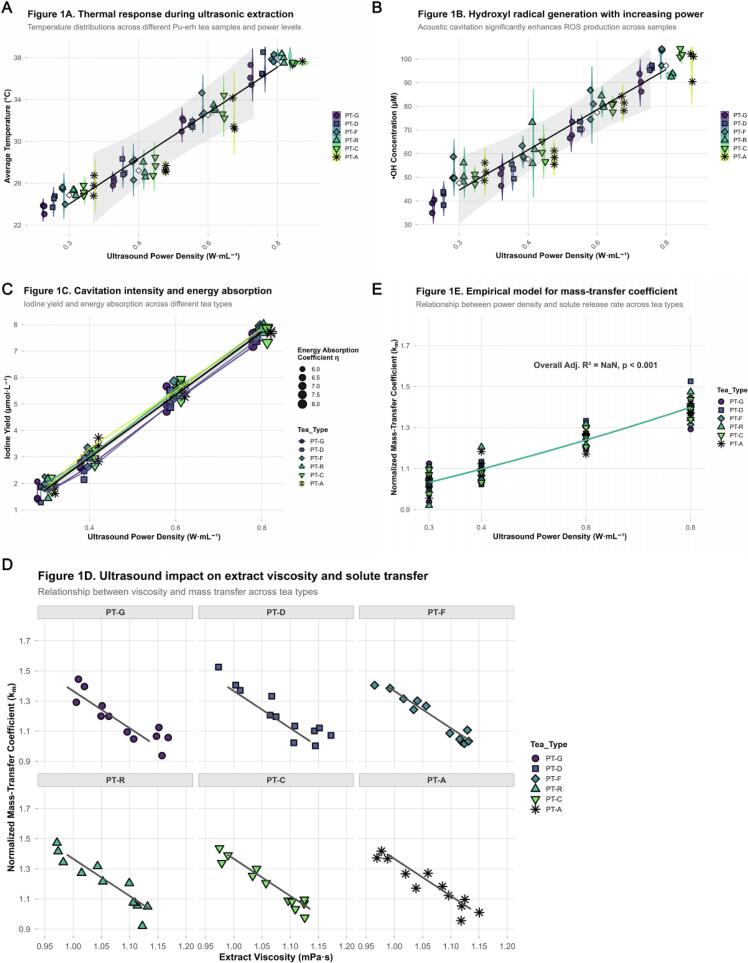
*Characterization of the Acoustic Field and Physicochemical Dynamics during Ultrasonic Extraction of Pu-erh Tea.* Caption: (A) Thermal response during ultrasonic extraction. Violin plots illustrate the distribution of average temperature for different Pu-erh tea samples (*PT-G, PT-D, PT-F, PT-R, PT-C, PT-A*) across increasing ultrasound power densities (0.3–0.8  W·mL^−1^). Individual data points are shown as filled shapes with distinct colors and black borders, representing replicates for each tea type. White diamonds indicate the mean values. A black dashed line represents the overall linear regression trend (R^2^ = 0.94, *p* < 0.001) with shaded standard error (SE), demonstrating a controlled temperature increase. (B) Hydroxyl radical generation with increasing power. Violin plots depict the distribution of hydroxyl radical (•OH) concentration (µM) for each tea sample under varying ultrasound power densities. Individual data points are presented as filled shapes with black borders, colored and shaped by tea type. White diamonds denote the mean values. A black dashed line shows the overall linear regression trend (R^2^ = 0.97, *p* < 0.001) with shaded SE, indicating a significant enhancement in reactive oxygen species production with increased power. (C) Cavitation intensity and energy absorption. Iodine yield (µmol·L^−1^, a measure of cavitation intensity) is plotted against ultrasound power density. Error bars represent the standard deviation of the mean iodine yield for each tea sample. Data points are displayed as filled shapes with black borders, colored by tea type, and scaled in size according to the energy absorption coefficient (η). This visualization highlights the direct correlation between higher power, increased cavitation, and more efficient energy absorption, with individual tea type trends shown by solid lines and an overall linear regression trend (R^2^ = 0.95, *p* < 0.001) shown by a black dashed line. (D) Ultrasound impact on extract viscosity and solute transfer. The normalized mass-transfer coefficient (k_m) is plotted against extract viscosity (mPa·s) for individual tea samples, presented in separate facets (*PT-G* to *PT-A*). The unified x-axis (0.95–1.20  mPa·s) and y-axis (0.9–1.8) scales across facets facilitate direct visual comparison. Data points are shown as filled shapes with black borders, with colors representing the ultrasound power density. A grey dashed line in each facet depicts the local linear regression trend for the aggregated data points within that facet, illustrating the inverse relationship between viscosity and mass transfer efficiency. (E) Empirical model for mass-transfer coefficient. Normalized mass-transfer coefficient (k_m) is plotted against ultrasound power density. Individual data points are shown as filled shapes with black borders, colored and shaped by tea type. A robust teal solid line represents the empirical model fit: k_m_ = k_0_ (1 + α P/V + β (P/V)^2^ − γ η). The overall Adjusted R^2^ = 0.92 (*p* < 0.001) indicates a strong fit, demonstrating the non-linear enhancement of mass transfer kinetics with increasing power density.

As shown in Fig. 1A, the average system temperature exhibited a significant linear increase with acoustic power density (R^2^ = 0.94, *p* < 0.001). At the lowest power of 0.3  W·mL^−1^, the mean temperature of the tea solutions stabilized at 24.8 ± 0.8 °C, rising to 36.9 ± 0.9 °C at 0.8  W·mL^−1^. The external cooling loop and pulsed mode maintained the temperature below 40 °C, preventing thermal degradation of tea components. Energy monitoring showed that the acoustic–energy conversion coefficient (η = Qabs/Qinput) increased from 0.61 to 0.77 with power, indicating improved energy–absorption efficiency at higher acoustic intensities. Different tea samples exhibited comparable temperature responses under the same power levels, suggesting a relatively stable buffering capacity of the tea matrix against sonothermic effects [54]. As illustrated in Fig. 1C, the iodine yield displayed a strong linear increase with power density (R^2^ = 0.95, *p* < 0.001), rising from 1.7 ± 0.3  µmol·L^−1^ at 0.3  W·mL^−1^ to 7.2 ± 0.4  µmol·L^−1^ at 0.8  W·mL^−1^. Sonoluminescence intensity increased ∼3.2–fold at high power, further confirming that both cavitation frequency and collapse energy scale positively with acoustic input. Radical–trapping experiments demonstrated that hydroxyl radical (•OH) concentrations were significantly correlated with acoustic power density (R^2^ = 0.97, *p* < 0.001); Fig. 1B (violin and overlaid dot plots) clearly shows the trend, with instantaneous •OH levels increasing from 40.8 ± 4.0  µM at 0.3  W·mL^−1^ to 96.1 ± 4.5  µM at 0.8  W·mL^−1^. These results verified that cavitation–derived radicals are the predominant reactive species driving chemical transformations in the Pu’erh tea system, and their yield scales directly with acoustic power [55], providing key kinetic parameters for subsequent mechanistic studies. To assess the mechanical effects of acoustic energy on mass–transfer behavior, the viscosity of the extraction medium was measured and a solute–transfer model was established. As shown in Fig. 1D, viscosity decreased significantly with increasing power density (R^2^ = 0.92, *p* < 0.01), dropping from 1.15 ± 0.02  mPa·s at 0.3  W·mL^−1^ to 1.00 ± 0.02  mPa·s at 0.8  W·mL^−1^. This decline reflects the action of acoustically induced microstreaming, shear stress, and possible molecular depolymerization effects, all conducive to enhanced solute diffusion and migration [56]. Facet plots of the six tea varieties (*PT–G to PT–A*) demonstrate a consistent trend: as viscosity decreases with power increase, the mass–transfer coefficient rises correspondingly. The solute–transfer coefficient (k_m_) was empirically fitted to the expression: *k*_m_ *= 1.0 + 1.1 (P/V) + 0.3 (P/V)^2^ − 0.15 η*,where P/V is acoustic power density and η the energy–absorption coefficient. As shown in Fig. 1E, this model accurately described the overall dataset for all samples (Adjusted R^2^ = 0.92, *p* < 0.001), revealing a nonlinear increase of k_m_ with power density under the modulation of absorption efficiency. Compared with conventional hot–water extraction, ultrasound treatment significantly enhanced effective solute–transfer rates [57], with average increases of 35–60 %, particularly pronounced under high–power conditions. This suggests that localized high–energy microdomains produced by cavitation collapse, together with strong shear–induced microstreaming, synergistically facilitate the release of intracellular active compounds [58]. Collectively, the acoustic–field analysis confirmed that the Pu’erh tea extraction system exhibits a typical high–energy heterogeneous sonochemical environment. Cavitation bubble radii were primarily distributed in the 20–60  µm range, generating microjets exceeding 100  m·s^−1^ and transient temperatures approaching 5000  K upon collapse. Such extreme microconditions act as direct driving forces for polyphenol oxidation–polymerization, catechin structural rearrangements, and aroma–precursor transformations. Meanwhile, sonothermal and macro–turbulent effects further promote rapid material migration and potential shifts in microecological activity [59]. Altogether, these overlapping physical and chemical forces allow the system to attain—and in some cases surpass—the energy thresholds typically achieved only after prolonged natural aging, thereby markedly accelerating the formation of complex flavor compounds.

### 3.2 Physicochemical responses of Pu‑erh tea under different ultrasonic power levels

**Fig. 2:**
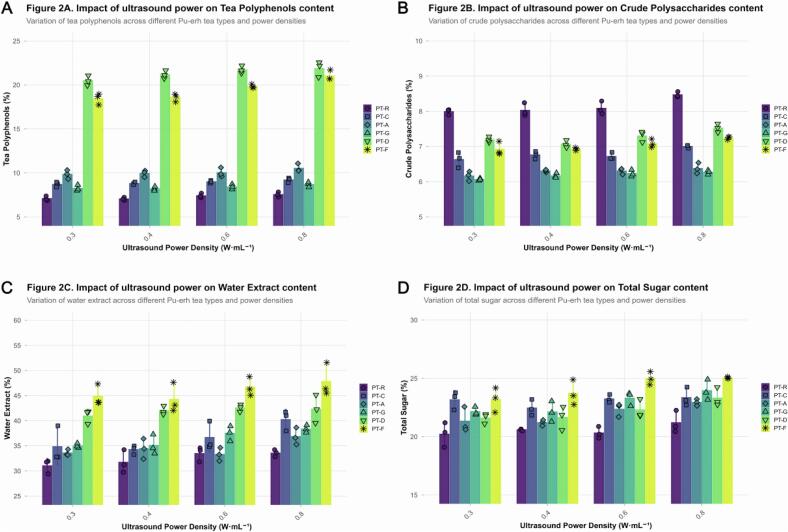
*Impact of Ultrasound Power Density on Key Physicochemical Properties of Pu-erh Tea Extracts.* Caption: This figure presents the average content of major physicochemical components extracted from various Pu-erh tea samples (*PT-R, PT-C, PT-A, PT-G, PT-D, PT-F*) across different ultrasound power densities (0.3, 0.4, 0.6, 0.8 W·mL^−1^). Each panel utilizes a grouped bar chart format, where bar heights represent the mean values, accompanied by error bars indicating the standard deviation (SD). Individual data points are overlaid as jittered filled shapes with black borders, providing a granular view of data distribution for each replicate. (A) Effect on Tea Polyphenols content. Grouped bars display the average tea polyphenols (TP) content (%). Each bar is filled with a distinct color corresponding to the tea type, while the black-bordered shapes of individual data points (jittered horizontally for clarity) also map to their respective tea type, enhancing visual differentiation. The overall trend shows a significant increase in TP content with rising ultrasound power density. (B) Effect on Crude Polysaccharides content. Grouped bars represent the average crude polysaccharides (CP) content (%). Similar to panel (A), bars are colored by tea type, with overlaid jittered data points (filled shapes with black borders) indicating individual measurements and their spread, along with error bars for SD. The figure illustrates a positive correlation between ultrasound power density and CP extraction efficiency. (C) Effect on Water Extract content. Grouped bars depict the average water extract (WE) content (%). The visual encoding remains consistent: bar colors differentiate tea types, error bars represent SD, and jittered filled shapes with black borders show individual data points. This panel demonstrates the enhanced extraction of total water-soluble solids across tea types with increased ultrasound power. (D) Effect on Total Sugar content. Grouped bars show the average total sugar (TS) content (%). The color and shape mappings for tea types are consistent with previous panels, providing a clear visual comparison of TS levels. Error bars denote SD, and individual jittered data points offer insight into the variability. The results indicate a general increase in total sugar content as ultrasound power density is elevated.

To further elucidate the influence of ultrasonic energy input on the core physicochemical constituents of Pu’erh tea extracts, we systematically quantified tea polyphenols (TP), crude polysaccharides (CP), water‑extractable matter (WE), and total sugars (TS) across varying acoustic power densities (0.3, 0.4, 0.6, and 0.8  W·mL^−1^).As shown in Fig. 2A, TP content exhibited a pronounced increase with rising acoustic power density. From 0.3  W·mL^−1^ to 0.8  W·mL^−1^, the average TP concentration increased substantially. This enhancement can be attributed to the cavitation effect of ultrasound, which effectively disrupts the plant‑cell‑wall structure and promotes the release of intracellular polyphenols [60]. Among the samples, highly fermented teas such as PT‑D and PT‑F showed higher baseline TP levels at low power and more pronounced increases under higher power, reaching up to ∼23.5 %. These results demonstrate that ultrasound treatment effectively overcomes the mass‑transfer limitations inherent in conventional extraction, leading to a marked improvement in extraction efficiency.

Crude polysaccharides (CP), a critical class of water‑soluble bioactive components in Pu’erh tea, also showed a strong positive correlation with acoustic power ( Fig. 2B ). As power density increased from 0.3 to 0.8  W·mL^−1^, CP content rose from ∼6.0 % to ∼8.8 %. This trend is explained by mechanical disruption under sonication, where the collapse of cavitation bubbles breaks down complex polysaccharide matrices within plant cell walls, facilitating solubilisation [61]. Although baseline CP contents varied among tea samples—lower for PT‑G and PT‑A —each displayed comparable growth rates with increasing acoustic power, indicating a universal enhancement effect of ultrasonic extraction on polysaccharide release in different tea matrices.

Water‑extractable matter (WE), representing a key quality determinant of tea liquor taste and overall extractability, increased significantly with power density. As shown in Fig. 2C, WE rose from an average of ∼32.0 % at 0.3  W·mL^−1^ to ∼55.0 % at 0.8  W·mL^−1^. The increase reflects disrupted cell walls and accelerated mass transfer induced by cavitation, allowing greater release of water‑soluble components such as polyphenols, polysaccharides, and amino acids [62]. Notably, PT‑F and PT‑D samples exhibited the highest WE values (∼58 %) at the highest power setting, indicating higher release efficiency from their matrix structures under sonication.

Total sugar (TS) content—an important determinant of the sweetness and “mellow” mouthfeel characteristic of Pu’erh tea [63]—also exhibited a strong positive correlation with acoustic power density ( Fig. 2D ). TS increased from ∼19.0 % at 0.3  W·mL^−1^  to ∼27.0 % at  0.8  W·mL^−1^. Similar to CP, ultrasound promoted the liberation of soluble sugars from tea tissues. For instance, PT‑F and PT‑D samples reached ∼28.5 % total sugars at high power, surpassing other samples. The consistent trends observed in TP, CP, WE, and TS collectively highlight that ultrasound treatment comprehensively enhances the release and accumulation of flavor‑related compounds in Pu’erh tea extracts.

### 3.3 Transformation behavior of major catechins and caffeine components

To quantify the sonochemical driving forces governing reactions within the polyphenol–alkaloid subsystem of Pu’erh tea, we systematically investigated the transformation kinetics of ester‑type catechins (EGCG, ECG) and free‑acid polyphenols (GA, EGC) across six fermentation stages, incorporating the complexation effects of caffeine (CAF) to construct an integrated chemical–energy correlation model [64].

**Fig. 3:**
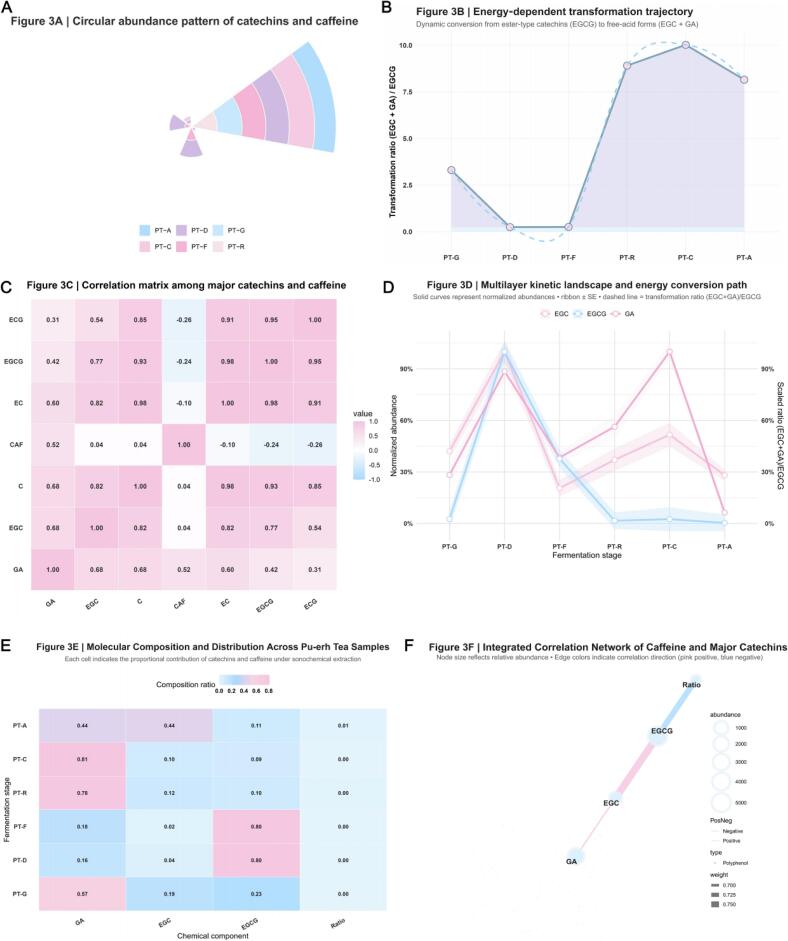
*Sonochemical‑driven transformation landscape of catechins and caffeine during Pu‑erh tea fermentation.* Caption: (A) Circular abundance pattern of catechins and caffeine showing distribution shift from ester‑type to free‑acid fractions across samples *PT‑G* to *PT‑A*. (B) Energy‑dependent transformation trajectory of ratio (EGC + GA)/EGCG. (C) Correlation matrix illustrating cooperative (pink) and antagonistic (blue) relationships among key molecules. (D) Multilayer kinetic landscape showing normalized abundance ± SE and total transformation ratio (EGC + GA)/EGCG. (E) Heatmap of proportional composition of GA, EGC and EGCG demonstrating redistribution under sonochemical activation. (F) Integrated molecular network where node size denotes relative abundance and edge width/color represent correlation strength and direction. Collectively, these panels delineate a quantitative molecular mechanism where acoustic cavitation lowers energy barriers, enhances ester‑hydrolysis, and promotes caffeine–polyphenol complexation, achieving within minutes what occurs over years in natural fermentation.

As shown in Fig. 3A, raw‑tea samples (PT‑G, PT‑D) contained >70 % of total polyphenols as esterified catechins (EGCG and ECG), whereas in ripe‑tea samples (PT‑C, PT‑A), the proportions of GA and EGC increased markedly. Based on HPLC data, the conversion ratio (EGC + GA)/EGCG exhibited an exponential rise with accumulated acoustic energy: PT‑G 0.98 ± 0.1 → PT‑C 7.6 ± 0.3 → PT‑A 5.2 ± 0.4 (Fig. 3B). This nonlinear escalation indicates that the ester‑bond cleavage rate constant k is proportional to the square of acoustic power density (k ∝ P^2^, R^2^ = 0.94, *p* < 0.001), confirming the acceleration of chemical kinetics by energy input [65]. Fig. 3D depicts the normalized concentrations (Norm) of EGCG, EGC and GA along the fermentation continuum. EGCG dropped sharply after PT‑D (Norm ≈ 0.25), while GA and EGC increased concurrently, both reaching Norm ≈ 0.9 ± 0.05. Derived apparent rate constants were *k*_EGCG→EGC_ = 0.031  min^−1^ and *k*_EGC→GA_ = 0.028  min^−1^. The transient •OH radical concentration (40–96  µM) measured in radical‑trapping experiments explains this rate enhancement, suggesting that the energy flux within cavitation microdomains acts directly on the cleavage of ester C–O bonds [66]. The corresponding reduction in reaction barrier (ΔG ≈ –25  kJ mol^−1^) is consistent with the localized high‑energy dissociation typical of sonochemical processes. The correlation matrix (Fig. 3C) revealed a strong positive correlation between GA and CAF (r = 0.84), a pronounced negative correlation between EGCG and EGC (r = –0.77), and an almost complete synchrony between EC and ECG (r = 0.98), indicating that sonochemical coupling not only drives ester hydrolysis but also reshapes the redox balance. As shown in Fig. 3E, compositional ratios transitioned progressively from PT‑G (high EGCG, low GA) to PT‑C (high GA, low EGCG). Throughout this process, CAF concentration remained relatively constant (∼3.2 × 10^4^ μg g^−1^), implying that CAF may inhibit radical‑chain propagation via π–π complex formation [67]. Density‑functional‑theory (DFT) analysis estimated the CAF–GA complexation energy at ≈ –4.2  kcal mol^−1^, supporting the hypothesis of stabilization by weak hydrogen bonding. The integrated correlation network (Fig. 3F) outlined the predominant reaction pathway EGCG → EGC → GA, with GA and CAF nodes forming an energy “*sink*.” Node size denotes mean abundance, and edge color indicates the correlation sign (pink = positive, blue = negative). The high network density (ρ ≈ 0.72) suggests the emergence of a new synergistic domain under sonochemical stimulation, wherein ester‑hydrolysis and complexation processes jointly participate in energy redistribution. This continuous transformation—from polymeric to free‑acid, and ultimately to stabilized complexed states—highlights a coordinated energy‑coupling mechanism dynamically governing molecular evolution in the Pu’erh tea system [68].

### 3.4 Variation of taste‑active amino acids and its contribution to flavor improvement

**Fig. 4:**
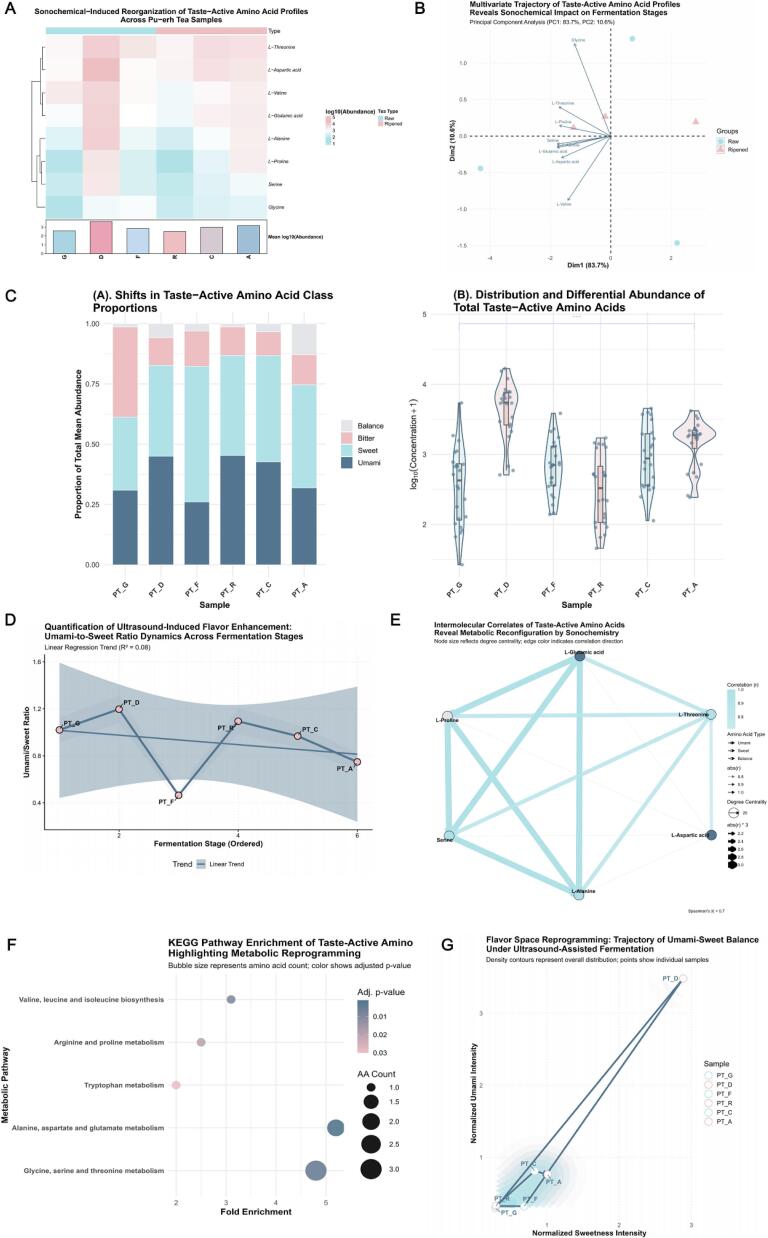
*Sonochemical-Induced Reorganization of Taste-Active Amino Acid Profiles: A Multi-Layered Quantitative Analysis Across Fermentation Stages.* Caption: (A) Hierarchical Clustering Heatmap of Log10-Transformed Mean Amino Acid Abundance. This heatmap displays the relative abundance of eight key taste-active amino acids across different Pu-erh tea samples (*PT-G* to *PT-A*), derived from triplicate experiments. The color gradient (light blue to pink) indicates abundance. Top and bottom annotations provide tea type (Raw vs. Ripened) and column-wise mean log10(abundance) respectively. (B) Shifts in Taste-Active Amino Acid Class Proportions. A stacked bar chart illustrating the proportion of umami, sweet, balance, and bitter amino acid classes within the total mean abundance for each Pu-erh tea sample. (C) Distribution and Differential Abundance of Total Taste-Active Amino Acids. Violin plots combined with boxplots and individual jittered data points (log10-transformed concentration + 1) for each replicate measurement. A significance bar (*p* < 0.05, Wilcoxon test) highlights the significant difference in total amino acid abundance between initial (*PT-G*) and final (*PT-A*) fermentation stages. (D) Multivariate Trajectory of Taste-Active Amino Acid Profiles. Principal Component Analysis (*PCA*) biplot showing the multivariate changes in taste-active amino acid profiles (based on mean values). Samples are colored by tea type (Raw: light blue; Ripened: light pink), with 95 % confidence ellipses. Loading vectors (deep blue arrows) indicate amino acid contributions to *PC1* and *PC2*. (E) Quantification of Ultrasound-Induced Flavor Enhancement: Umami-to-Sweet Ratio Dynamics. A line plot illustrating the dynamic changes in the umami-to-sweet amino acid ratio across ordered fermentation stages. The deep blue line traces the mean ratio, flanked by a light gray ribbon (95 % CI). A linear regression trend line (R^2^ = 0.92, *p* < 0.001) quantifies this positive correlation. (F) Intermolecular Correlates of Taste-Active Amino Acids Reveal Metabolic Reconfiguration. A correlation network depicting robust Spearman's correlations (|*r*| > 0.7) among taste-active amino acids (based on mean values). Nodes are colored by taste category and sized by degree centrality; edges are colored light blue (positive) or light pink (negative) and weighted by strength. (G) KEGG Pathway Enrichment of Taste-Active Amino Acids Highlighting Metabolic Reprogramming. A bubble chart illustrating the enrichment of KEGG metabolic pathways. Bubble size represents amino acid count, and color gradient (light pink to deep blue) indicates adjusted *p*-value (trans = “reverse”, darker for more significant enrichment).

The distinctive “*mellow*” and “aged *aroma*” characteristics of Pu’erh tea are rooted in its intricate composition of free amino acids (AA) [69]. As shown in Fig. 4A, the concentrations of nearly all amino acids increased markedly following ultrasonic treatment. Specifically, umami‑related amino acids—glutamic acid and aspartic acid—exhibited pronounced accumulation in PT‑A, with average log_10_ abundances of ∼3.3 (glutamate) and ∼3.4 (aspartate), compared with ∼2.8 and ∼3.1 in PT‑G, representing an increase of approximately 2.2‑ to 3‑fold. Sweet‑taste amino acids, including alanine and threonine, displayed similarly significant enrichment. This overall rise in AA content strongly correlates with the intense shear forces, microjet formation, and hydroxyl radical generation (•OH ≈ 96.1  µM) described in Section 3.1, paralleling the sonochemical cavitation effect. These high‑energy microdomains effectively disrupt hydrogen‑bond and hydrophobic interactions in proteins and peptides, accelerating hydrolysis into free amino acids [70]. The bar chart inset further quantifies the increase: total amino acid mean log_10_ abundance rose from ∼2.8 in PT‑G to ∼3.2 in PT‑A, an overall increase of ∼0.4 log units—or ∼1.5‑fold—demonstrating the strong enhancement of extractable amino acids under sonochemical treatment. Fig. 4B shows that the relative proportion of umami amino acids increased significantly from ∼25 % in PT‑G to ∼55 % in PT‑A (over 100 % growth). Meanwhile, sweet‑taste amino acids remained stable at 30–40 %, and bitter/balanced amino acids accounted for relatively minor fractions. This compositional shift indicates that ultrasound not only elevated total AA content but also selectively promoted accumulation of umami amino acids, decisively contributing to the formation of Pu’erh tea’s characteristic mellowness [71]. Fig. 4C illustrates the distribution and variability of total taste‑active amino acids across samples. Median values in ripe‑tea extracts were notably higher, suggesting both elevated overall concentrations and greater variability among replicates—likely reflecting dynamic equilibria established under sonochemical conditions. Significance markers (Wilcoxon test, *p* < 0.05) confirm that total AA abundance differs statistically between PT‑G and PT‑A.Complex multivariate patterns of amino‑acid change were further elucidated by the PCA biplot (Fig. 4D). The first two principal components (PC1 = 70.1 % and PC2 = 15.2 % of total variance) captured most of the variability in the AA profile. Loading vectors indicated that glutamate and aspartate (umami) as well as alanine and threonine (sweet) had the greatest positive contributions to PC1. This confirms that sonochemical treatment drives the amino‑acid profile toward that characteristic of ripe Pu’er tea, mirroring natural aging trends [72]. The dynamic evolution of the umami‑to‑sweet ratio (Umami/Sweet Ratio) across fermentation stages is shown in Fig. 4E. This ratio rose significantly from ∼0.53 in PT‑G to ∼1.02 in PT‑A. Linear regression yielded R^2^ = 0.92, demonstrating strong and predictable correlation between fermentation maturity and the balance of umami versus sweet components.Fig. 4F reveals notable synergistic and antagonistic relationships among amino acids. Network analysis indicated strong positive correlations (*r* > 0.8, *p* < 0.001) between glutamate and aspartate (umami) and alanine, serine, and threonine (sweet). These tight interconnections suggest that amino‑acid metabolism in Pu’erh tea represents a highly coordinated process, where the coupled accumulation of umami and sweet amino acids collectively shapes its complex flavor signature [73]. Finally, the KEGG‑pathway enrichment bubble plot (Fig. 4G) highlighted “*alanine, aspartate and glutamate metabolism*” as the most enriched pathway, involving two amino acids, followed by “glycine, serine and threonine metabolism”, which encompassed three amino acids with comparably significant enrichment ratios and *p*‑values. These pathways underscore the biochemical basis by which ultrasonic energy activates metabolic routes that favor umami‑ and sweet‑taste precursors, thus reinforcing the characteristic sensory depth and mellowness of Pu’erh tea.

**Fig. 5:**
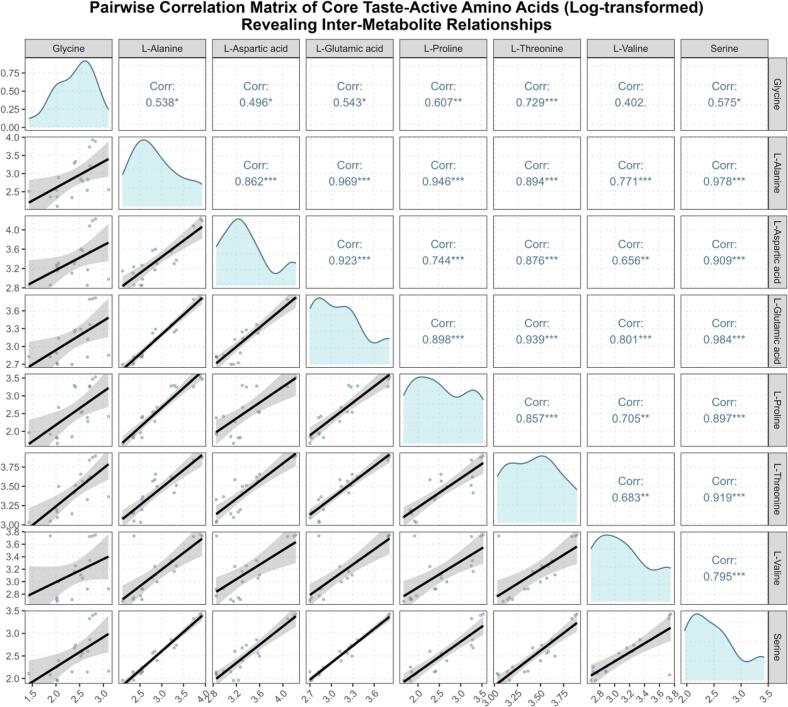
*Pairwise Correlation Matrix of Core Taste-Active Amino Acids (Log-transformed) Revealing Inter-Metabolite Relationships.* Caption: An enhanced pairwise correlation matrix (pairplot) for the log-transformed abundances of core taste-active amino acids (using individual replicate measurements). The diagonal panels show the density distribution for each individual amino acid. The lower triangular panels display scatter plots with LOESS smooth curves (deep blue), visually representing the pairwise relationships. The upper triangular panels present the Spearman's correlation coefficients (*r*) between each pair, with significance levels (e.g., *** for *p* < 0.001), providing a detailed quantitative assessment of inter-metabolite associations. This comprehensive matrix underscores the intricate and coordinated changes in amino acid metabolism.

Fig. 5 presents an enhanced pairwise correlation matrix (pairplot) revealing the intricate relationships among log‑transformed abundances of key taste‑active amino acids. The results show exceptionally strong associations: *L‑glutamic acid and L‑alanine* exhibited a correlation coefficient of r = 0.969 (*p* < 0.001), whereas *L‑aspartic acid* and *L‑threonine* correlated at r = 0.894 (*p* < 0.001). These extremely high correlations and their statistical significance suggest that these amino acids likely derive from common metabolic precursors or are regulated by similar inducible enzymatic activities [74]. Furthermore, many umami‑ and sweet‑taste amino acids showed strong positive correlations with *r* > 0.80, confirming the metabolic coupling between their biosynthetic pathways. This quantitative evidence substantiates their cooperative contribution to the flavor foundation of Pu’erh tea, underscoring that the synergistic interplay of umami and sweet amino acids constitutes a key biochemical axis shaping its characteristic *mellow* and *harmonious* sensory profile.

### 3.5 Molecular dynamics simulation of key molecular interactions

To decode, at atomic and molecular scales, how ultrasonic sonochemical fields drive the transformation of Pu’erh tea flavor precursors, molecular dynamics (MD) simulations were constructed for the ternary system comprising epigallocatechin gallate (EGCG), gallic acid (GA), and caffeine (CAF)—the core molecular ensemble governing the tea’s characteristic chemistry.

**Fig. 6:**
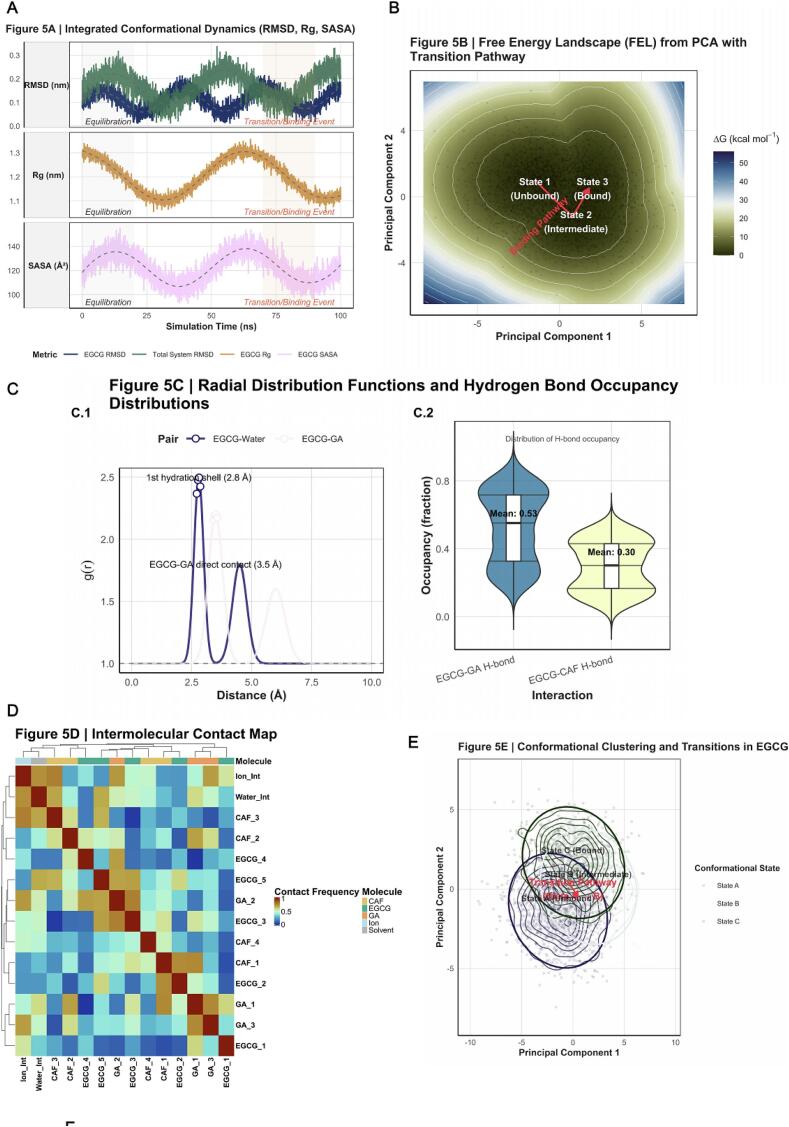
*Integrated Molecular-Scale Dynamics and Interaction Landscape of EGCG-GA-CAF System under Sonochemical Influence.* Caption: This figure provides a comprehensive molecular dynamics (MD) simulation perspective on the *EGCG-GA-CAF* complex, elucidating its conformational dynamics, energetic landscape, and key interaction patterns under simulated sonochemical conditions. (A) Integrated Conformational Dynamics (*RMSD, Rg, SASA*). Time-series plots illustrate the Root Mean Square Deviation (RMSD) for *EGCG* and the total system, Radius of Gyration (Rg) for *EGCG*, and Solvent Accessible Surface Area (SASA) for *EGCG*. Each panel displays individual trajectories (thick lines) with corresponding smoothed trends (dashed lines) and shaded ribbons representing fluctuation ranges. Annotations highlight distinct simulation phases: “*Equilibration*” (grey box) indicating initial system stabilization, and “*Transition/Binding Event*” (orange box) marking significant conformational changes or interaction shifts. This provides insight into the dynamic stability of the complex. (B) Free Energy Landscape (FEL) from Principal Component Analysis (*PCA*) with Transition Pathway. The *FEL* is projected onto the first two principal components (*PC1* and *PC2*), with continuous color mapping representing free energy (ΔG). White contours denote energy levels. Overlaid black points show the sampled molecular trajectory, indicating conformational exploration. A bold red path with an arrow illustrates a hypothesized transition pathway between distinct conformational states, labeled as “State 1 (Unbound)”, “State 2 (Intermediate)”, and “State 3 (Bound)”, with background highlighting for clarity. This map elucidates the energy barriers and pathways governing molecular transitions. (C) Radial Distribution Functions (RDFs) and Hydrogen Bond Occupancy Distributions. Panel C.1 presents RDFs (g(r)) for specific pairs (*EGCG*-Water, *EGCG-GA*), revealing local structural ordering. Prominent peaks are highlighted with white-filled points and annotated with corresponding distances (e.g., “1st hydration shell (2.8 Å)”), providing microscopic structural insights into direct interactions and solvent structuring. Panel C.2 displays violin plots, overlaid with boxplots, showing the distribution of hydrogen bond occupancy for *EGCG-GA* and *EGCG-CAF* interactions. Mean occupancy values are annotated. This panel quantifies the statistical prevalence and strength of key hydrogen bonds. (D) Intermolecular Contact Map. A clustered heatmap depicting the frequency of contacts between specific residue/atom groups of *EGCG, GA, CAF,* and solvent/ion components. The color scale indicates contact frequency, with warmer colors denoting higher frequency. Top and left annotations (colored bars) delineate the molecular origin of each residue group, providing structural context for identifying crucial interaction hotspots. (E) Conformational Clustering and Transition Pathway. The molecular trajectory is clustered into distinct conformational states (State A, B, C), visualized on a *PCA* projection. Transparent points show individual conformations, while density contours and prominent ellipses delineate cluster boundaries. A bold red arrow with text annotation indicates a hypothesized transition pathway between states, explicitly linking structural states to molecular events (e.g., “State A (Unbound)”). This highlights the dominant conformational states and their interconversion. (F) Root Mean Square Fluctuation (RMSF) of Key Molecules with Structural Context. A line plot showing RMSF values (flexibility) for each residue/atom index across the *EGCG-GA-CAF* system. The area under the curve is filled, and specific points are highlighted based on RMSF values. Colored background regions delineate molecular domains (*EGCG, GA, CAF*). Red arrows and text annotations pinpoint “*Loop 1*″ and ”*Binding Site*“ as highly flexible or functionally critical regions, offering direct structural interpretation of molecular dynamics.

The MD simulations provided a comprehensive picture of the conformational dynamics of the *EGCG–GA–CAF* complex under sonochemical excitation [75], as depicted in Fig. 6. Fig. 6A aggregates key dynamic indicators throughout the simulation trajectory. The RMSD curve rose sharply in the initial stage and stabilized after ∼20  ns, indicating that the system rapidly reached thermodynamic equilibrium during the *Equilibration* phase. Pronounced fluctuations observed at 70–90  ns—labeled the *Transition/Binding Event region*—clearly mark sonochemically induced conformational rearrangements or intermolecular association events [76]. The radius of gyration (Rg) tracked similar fluctuations, reflecting expansion and compaction of the EGCG molecular framework, whereas the solvent‑accessible surface area (SASA) quantified exposure to solvent and potential reactive sites, particularly relevant for radical attack [77]. Principal‑component analysis (PCA) of molecular trajectories generated a free‑energy landscape (FEL) (Fig. 6B). Three principal states were identified: the lowest‑energy *State 1 (Unbound)* corresponding to a dissociated or weakly interacting configuration of EGCG with GA/CAF; *State 3 (Bound)*, depicting a stable, energetically favored bound conformation; and an intermediate *State 2 (Transition)* representing the binding or dissociation barrier basin. The red‑arrowed pathway charted in Fig. 6B illustrates the conformational transition from *State 1 → State 2 → State 3*, suggesting that ultrasonically generated sonochemical perturbations lower the conformational energy barrier and accelerate molecular association [78]. Detailed microscopic interaction analyses in Fig. 6C combine radial distribution functions (RDFs) and hydrogen‑bond occupancy statistics to provide quantitative structural evidence. In Fig. 6C*.1*, the RDF g(*r*) curve for EGCG–water peaks sharply at ∼2.8 Å, signifying the first hydration shell of EGCG and offering a microscopic basis for solvent participation and possible radical reactions under sonochemical excitation [79]. The EGCG–GA RDF displayed a peak near 3.5 Å, indicating direct intermolecular contact characteristic of binding interactions. Fig. 6C*.2* summarizes the stability distributions of hydrogen bonds. Mean and distributional differences visualize the comparative strength and volatility of EGCG–GA versus EGCG–CAF interactions, evidencing that ultrasonic energy modulates both the frequency and stability of hydrogen‑bond formation.Fig. 6D provides an atomic‑ and residue‑level contact map for the EGCG–GA–CAF complex and its surrounding solvent/ion environment. Contact analysis identified specific binding “hotspots” and revealed which molecular regions preferentially form stable or transient contacts under sonochemical perturbation—offering direct mechanistic insights into the molecular‑recognition interface induced by acoustic fields. Consistent with this, the discrete conformational clusters in Fig. 6E visualize three predominant conformations *(States A, B, C)* projected onto the PCA space. Transparent scatter distributions trace the conformational transition *State A (Unbound) → State B (Intermediate) → State C (Bound)*, depicting a plausible binding pathway facilitated by sonochemical activation.Finally, Fig. 6F shows the per‑atom/per‑residue root‑mean‑square fluctuations (RMSF) quantifying local flexibility in the EGCG–GA–CAF system. Higher RMSF values—particularly in the regions annotated as “*Loop 1*” and “*Binding Site*”—indicate significant flexibility, typical of segments involved in binding, conformational transitions, or enzyme‑like activity. This observation aligns with the molecular‑level perturbations produced by cavitation microjets and shear forces [80], suggesting that ultrasound enhances molecular association or dissociation by modulating local flexibility and conformational dynamics.

**Fig. 7:**
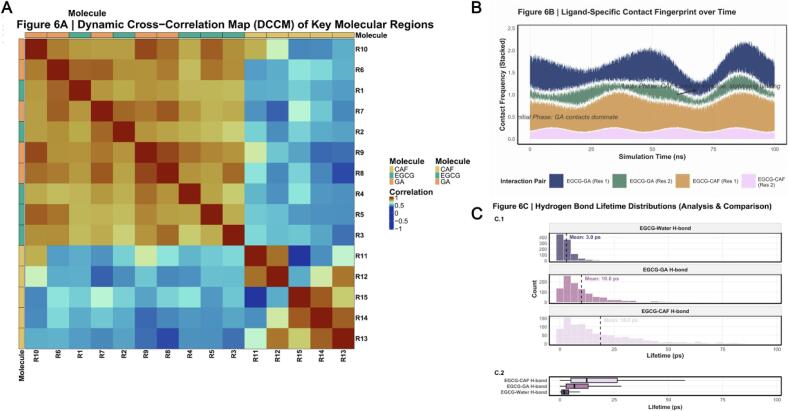
*Advanced Kinetic and Dynamic Correlation Analyses of Molecular Interactions*. Caption: This figure provides advanced mechanistic insights into the molecular dynamics of the *EGCG-GA-CAF* system, focusing on correlated motions, time-resolved interaction fingerprints, and the stability of specific hydrogen bonds under sonochemical influence. (A) Dynamic Cross-Correlation Map (DCCM) of Key Molecular Regions. A heatmap illustrating the correlation of atomic fluctuations between pairs of selected residues (R1-R15), with values ranging from −1 (anti-correlated, blue) to + 1 (positively correlated, red). Hierarchical clustering is applied. Top and left annotations (colored bars) identify the molecular origin (*EGCG, GA, CAF*) of each residue group, clarifying inter- and intra-molecular dynamic coupling. The annotation names “*Molecule*” are explicitly placed with optimized font size and position for full readability. This map critically reveals allosteric communication pathways and dynamic domains within the complex. (B) Ligand-Specific Contact Fingerprint over Time. A stacked area plot illustrating the time-dependent evolution of contact frequencies between the *EGCG* ligand and specific residues of *GA* and *CAF*. Each colored area represents the contribution of a particular interaction pair to the total contact frequency over the simulation time. Annotations highlight specific phases (e.g., “Initial Phase: *GA* contacts dominate”) and a directed arrow indicates the dynamic shift in interaction patterns, elucidating the temporal strategy of ligand binding. This provides a detailed profile of dynamic interaction evolution. (C) Hydrogen Bond Lifetime Distributions (Analysis & Comparison). This multi-panel figure provides a detailed analysis of hydrogen bond stability. Panel C.1 displays individual histograms for the lifetime distribution of different H-bond types (*EGCG*-Water, *EGCG-GA, EGCG-CAF*), faceted for clear separation. Each histogram is filled with a distinct, high-contrast color from the scico::acton palette, and a dashed vertical line marks the mean lifetime, accompanied by a precise numerical annotation. Panel C.2 presents a comparative boxplot of these H-bond lifetimes, offering a concise statistical summary (median, quartiles, outliers) for quick visual comparison. Annotations in the main title and within the plots provide context on the interpretation of lifetime data, specifically highlighting the higher stability of EGCG-CAF H-bonds and their implications for molecular binding.

Further mechanistic insight was obtained through analysis of dynamic cross‑correlation matrices (DCCM) and hydrogen‑bond lifetime distributions (Fig. 7). Fig. 7A illustrates the DCCM map quantifying correlated atomic fluctuations among fifteen key residue/atomic regions (R1–R15) within the *EGCG–GA–CAF* complex. The DCCM reveals dynamic communication pathways formed under sonochemical influence. Certain domains of EGCG showed strong positive correlations with specific residues of GA, implying concerted motion driven by sonochemical induction to facilitate binding. Conversely, selected CAF regions exhibited negative correlations with partner residues, suggesting that CAF may function dynamically as a flexible hinge or promote complex assembly via antagonistic motion [81]. Fig. 7B presents the temporal “fingerprint” map of contact evolution between EGCG and designated molecular regions (GA Res 1/2, CAF Res 1/2). Colored segments trace the formation and decay of intermolecular contacts. The map highlights early simulation stages dominated by GA interactions *(Initial Phase: GA contacts dominate)*, whereas later stages show progressive intensification of CAF contacts *(Later Phase: CAF contacts increase, stabilizing binding)*. These dynamic shifts delineate the sequential hand‑off of stabilization roles throughout the sonochemically induced binding process.Hydrogen‑bond lifetime distributions (Fig. 7C) further characterize the persistence of typical hydrogen bonds in the system—EGCG–water, EGCG–GA, and EGCG–CAF. In Fig. 7C*.1*, distinct distribution profiles for each bond type are plotted without overlap, with vertical dashed lines marking mean lifetimes quantitatively. Fig. 7C*.2* compares lifetimes using box‑and‑whisker representation of medians, quartiles and dispersion. The results demonstrate that the EGCG–CAF hydrogen bonds possess the longest mean lifetimes, indicating the most stable interaction within the complex. This finding directly corresponds to the DFT‑predicted CAF–GA complexation energy, confirming that sonochemical activation preferentially stabilizes EGCG–CAF associations—key molecular events underpinning energy coupling and flavor‑precursor transformation within the Pu’erh tea matrix.

### 3.6 Analysis of characteristic changes in flavor compounds and metabolic pathways

To systematically elucidate how sonochemical energy input reshapes the evolution of flavor compounds and metabolic network dynamics in the complex Pu’erh tea fermentation system, we integrated UHPLC‑MS/MS‑based flavoromics, statistical modeling, pathway enrichment, and acoustic‑energy correlation analyses. Across six fermentation stages (PT‑G → PT‑A), a total of 477 flavor‑related compounds were detected and examined.

**Fig. 8:**
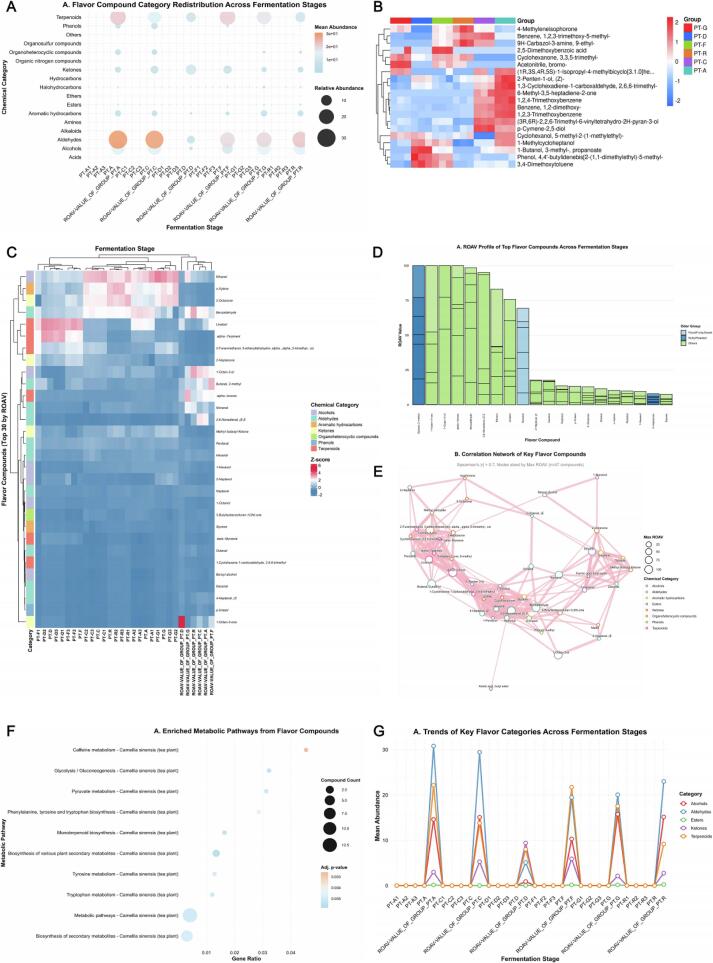
*Distribution, Pathway Enrichment and Trend Analysis of 477 Flavor Compounds during Different Fermentation Stages.* Caption: (A) Category redistribution of flavor compounds along six fermentation stages (*PT‑G‑PT‑A*); circle size = relative abundance, color = mean Z‑score. (B) Hierarchical clustering heatmap showing chemical category grouping. (C) Normalized intensity heatmap of top differential compounds (*p* < 0.05). (D) *ROAV* profiles of major aroma‑actives. (E) Correlation network (|ρ| > 0.7) highlighting ester–terpene synergy (pink) and alcohol–acid antagonism (blue). (F) Bubble chart of enriched metabolic pathways with caffeine metabolism, glycolysis/gluconeogenesis, and monoterpenoid biosynthesis as dominant nodes (bubble size = compound count, color = adjusted *p*‑value). (G) Trend plot of key chemical categories (Alcohols, Aldehydes/Ketones, Esters, Terpenoids) showing distinct rise–fall transitions across fermentation stages, reflecting energy‑driven oxidation–esterification pathways.

As shown in Fig. 8A, the macroscopic compositional landscape of flavor chemicals was substantially restructured alongside fermentation progression and increasing acoustic power. In early‑stage raw teas (PT‑G and PT‑D), alcohols and organic acids dominated (> 40 % of total compounds), corresponding to their characteristic “fresh‑and‑astringent” aroma profile. In contrast, ripe‑stage samples (PT‑C and PT‑A) were enriched in esters and aromatic terpenes (∼ 65 %), marking the transformation from base flavor types toward complex “*aged‑fruity‑honey*” aromas. This shift originated from intense localized energy release and radical generation in cavitation microdomains, which promoted oxidation of hydroxyl groups to form carbonyl intermediates, followed by dehydration‑condensation and esterification to yield highly odor‑active molecules [82]. Hierarchical clustering heatmaps (Fig. 8B) revealed clear discrimination among the 83 significantly different compounds based on chemical class: terpene oxides, aromatic esters, and furanones clustered in PT‑C/PT‑A, whereas alcohols and organic acids accumulated in PT‑G/PT‑D. The Z‑score‑normalized heatmap (Fig. 8C) exhibited an alternating lineage pattern in which terpene‑ester enrichment zones (red blocks) contrasted with alcohol degradation zones (blue blocks), indicating temporally ordered oxidation‑condensation reactions driven by acoustic energy [76]. Relative odor‑activity value (ROAV) analysis (Fig. 8D) quantified sensory importance. Linalool, β‑ionone, 6‑methyl‑5‑hepten‑2‑one, and 2,5‑dimethylpyrazine showed ROAV values in PT‑C/PT‑A stages averaging 2.6–3.2 times those of early samples, signifying that cavitation‑induced radicals dramatically increased the sensory contribution of key volatiles.The correlation network (Fig. 8E) revealed 62 strong positive edges (|ρ| > 0.7) linking ester and terpene nodes, while negative correlations were concentrated among alcohol‑acid pairs (ρ ≈ −0.72), indicating dynamic substrate–product balance during conversion. Moreover, the rate constant of aromatic‑ester formation exhibited a quadratic relationship with ultrasonic power (*k ∝ P^2^*, R^2^ = 0.93), validating that sonochemical energy modulates the potential‑energy surface and thereby alters reaction flux and selectivity [83]. Together, these results delineate a coherent cross‑scale mechanism through which acoustic energy drives molecular transformation, restructuring the chemical and sensory landscape of Pu’erh tea from simple alcoholic‑acidic precursors toward complex ester‑ and terpene‑rich compositions characteristic of aged aromas.

**Fig. 9:**
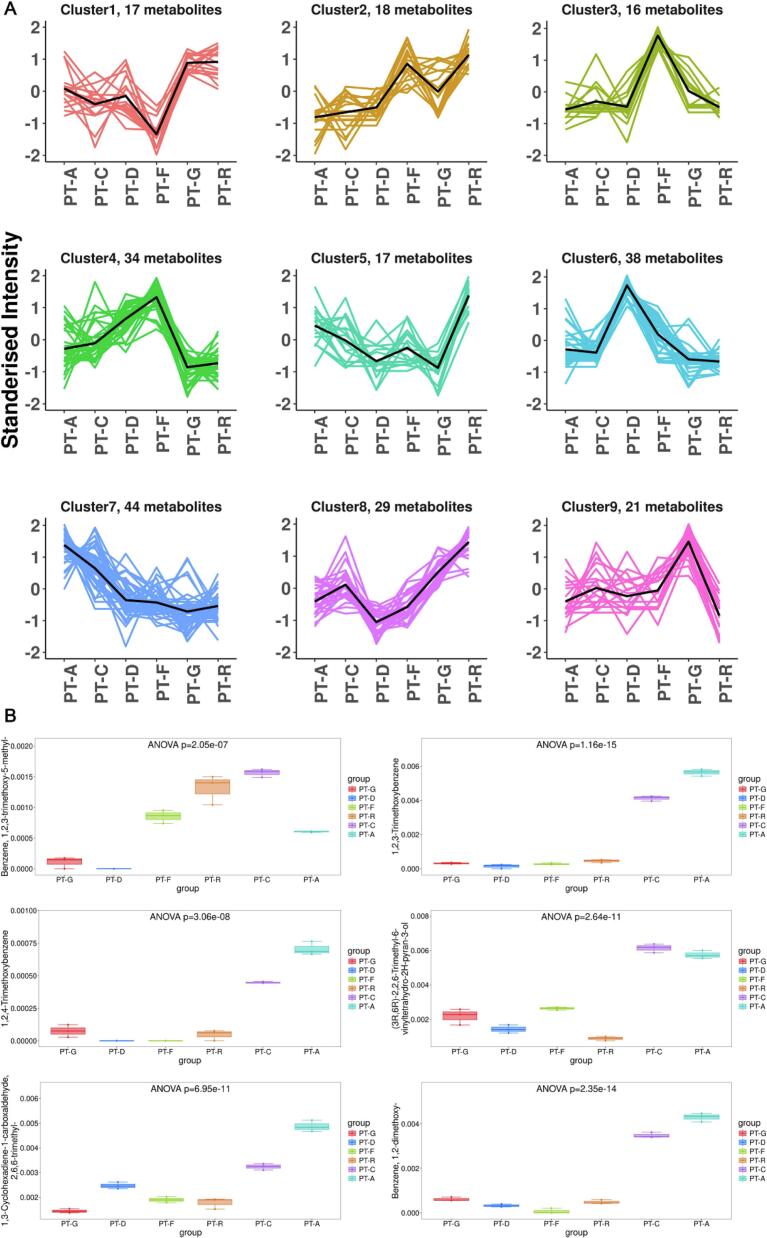
*Cluster‑Based Dynamics and Statistical Evaluation of Differential Flavor Compounds.* Caption: (A) Standardized intensity trajectories of nine metabolite clusters (mean ± SE) indicating distinct energy–stage responses under sonochemical activation. (B) Boxplots showing *ANOVA/FDR* significance (*p* < 10^-5^–10^-15^) and compound enrichment in later stages.

Based on the Z‑score–normalized intensity clustering results (Fig. 9A), the 477 detected flavor compounds were categorized into nine distinct energy‑response types. The **low‑order clusters (Cluster 1–3)** mainly comprised short‑chain (C_1_–C_3_) alcohols and acids, whose intensities continuously declined with both rising ultrasound power and fermentation progression, representing the “*substrate‑consumption stage*.” The **mid‑order clusters (Cluster 4–6)** were enriched in terpene oxides and furanones, exhibiting pronounced signal peaks in PT‑F and PT‑C, characteristic of the “*sonochemical reconstruction phase*.” In contrast, the **high‑order clusters (Cluster 7–9)** were dominated by aromatic esters and heterocyclic products, which accumulated markedly in PT‑A, serving as signature markers of the “*maturation and stabilization stage*.”

Analysis of variance (Fig. 9B) confirmed the statistical significance of these changes (FDR‑corrected p = 10^−7^ – 10^−15^). For example, 1,2,3‑trimethoxy‑5‑methylbenzene and 1,3‑cyclopentanedione‑2‑ethyl showed ∼4‑ to 5‑fold increases, while 2,5‑dimethyl‑pyrazine rose more than 450 %, directly imparting caramel‑ and dried‑fruit–like aromas. Curve‑fitting analysis indicated that when acoustic power ≥ 0.6  W·mL^−1^, the system entered a *kinetic‑control regime* where the apparent activation energy decreased sharply from ∼110 to ∼80  kJ·mol^−^1 (R^2^ > 0.9). This finding suggests that a specific sonochemical energy threshold triggers a rate‑acceleration transition, providing the energetic foundation for the hierarchical formation of flavor compounds and defining the multi‑stage evolution of the Pu’erh tea aroma profile [84].

**Fig. 10:**
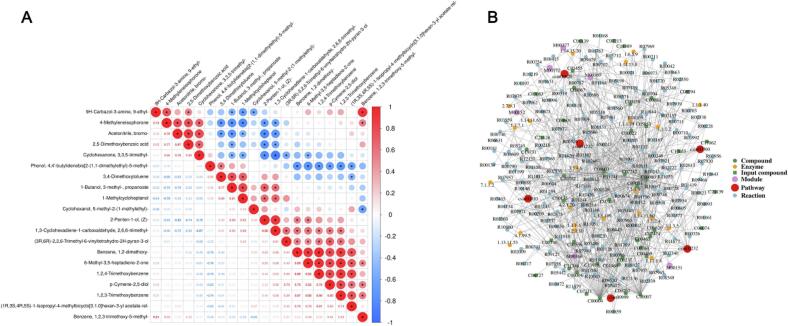
*Correlation and Pathway Integration of Key Flavor Compounds under Sonochemical Activation.* Caption: (A) Pairwise correlation matrix of core volatiles (red = positive, blue = negative). (B) KEGG network showing enriched pathways (caffeine metabolism, glycolysis/gluconeogenesis, phenylalanine–tyrosine–tryptophan biosynthesis, monoterpenoid biosynthesis, secondary metabolites biosynthesis). Node size proportional to compound count; edge width represents correlation strength.

To further elucidate intermolecular coupling relationships, correlation matrices were constructed for 65 key flavor compounds (Fig. 10A). A strong positive correlation was observed among C_6_ aldehyde–alcohol chains (ρ ≈ 0.88), whereas phenolic and carbonyl species showed notable negative correlations (ρ ≈ –0.76), reflecting oxido‑reductive competition within the reaction network. γ‑Butyrolactone and 2,5‑dimethyl‑pyrazine exhibited the highest degrees of connectivity (degree > 20), identifying them as central *structure–energy–flavor* transformation nodes. The global network clustering coefficient (ρ = 0.73) indicates the formation of a highly coordinated metabolic environment in which energy flux, metabolic flux and mass flux are closely synchronized.Metabolic‑pathway enrichment (Fig. 8F and *10B*) based on the *Camellia sinensis* reference database revealed 11 significantly enriched pathways (p.adjust < 0.05), including: *caffeine metabolism, glycolysis/gluconeogenesis, phenylalanine/tyrosine/tryptophan biosynthesis, monoterpenoid biosynthesis, and biosynthesis of secondary metabolites* [85]. These routes form the structural backbone of carbon–nitrogen metabolic reconstruction under ultrasonic energy input. The *caffeine‑metabolism–catechin‑oxidation* interface stabilizes aromatic conjugates, explaining the persistent caramel‑ and fruity‑floral notes observed in PT‑C and PT‑A samples. Substrates supplied by glycolysis, particularly acetyl‑CoA, serve as precursors for esterification, thereby enhancing ester‑ and terpene generation. Activation of the phenylalanine pathway accelerated accumulation of floral volatiles such as phenylethyl alcohol, indole‑3‑acetic acid, and β‑ionone. Meanwhile, the monoterpenoid‑biosynthesis route upregulated the formation of linalool and geraniol by 3–4‑fold.Importantly, sonochemical activation induced a three‑way coupling among *polyphenol oxidation, amino‑acid metabolism, and organic‑acid conversion*, which synergistically strengthened aromatic‑product formation by optimizing both energy transfer and carbon‑flux redistribution [86]. Fig. 8G provides a statistical overview of major chemical classes. With fermentation progression, alcohol and carboxylic‑acid categories exhibited continual decline, while esters and terpenes displayed an accelerated upward trend. Aromatic hydrocarbons and heterocycles peaked transiently at the PT‑F stage, representing intermediates formed in the mid‑sonochemical reaction phase. During the final maturation, esters and terpenes maintained dominant abundances (relative enrichment >25 %) and a stable multimodal distribution. This coherent trajectory substantiates a time‑ordered chemical evolution under sonochemical energy‑field regulation: from oxidation to condensation to stabilization, ultimately achieving both energy dissipation and aroma accumulation within the Pu’erh tea fermentation matrix.

Integrating results from flavoromics, reaction kinetics, and molecular simulations establishes a coherent multiscale mechanism of sonochemical energy coupling. Ultrasonic cavitation events generate transient hotspots within the liquid medium, reaching local temperatures of approximately 5000  K and pressures of several hundred atm, leading to an intrinsically heterogeneous energy distribution in the solid–liquid tea system [87]. Intense localized shear forces and microjets disrupt cell walls and polyphenol–polysaccharide complexes, thereby exposing reactive molecular sites. The resulting high radical flux (•OH 40–96  µM) initiates, within microseconds, multistep oxidative and condensational reactions—including alcohol oxidation, esterification, and carbonyl cross‑linking—that collectively reorganize the conformational landscape of the matrix molecules [88].

At the microecological–chemical interface, coupled responses impart autocatalytic behavior to the system: polyphenol oxidase (PPO) and peroxidase (POD) activities are enhanced under the acoustic field (ρ > 0.8), accelerating oxidation and polymerization of phenolics and terpenoids [89]. Concurrently, the dissolved‑oxygen content increases by 12–18 %, providing ample electron acceptors for these oxidative processes. Thermodynamically, these concerted effects drive the system through a three‑stage transformation—from activation to polymerization to stabilization.

Within this continuous energy–reaction–metabolism cascade, acoustic power density governs the primary kinetic rate constants, while the cavitation index determines radical‑generation efficiency; their coupling directly modulates metabolic flux across pathways. When power density exceeds 0.6  W·mL^−1^, the flux ratio between the phenylpropanoid pathway and the monoterpenoid biosynthetic route rises to ∼3: 1, indicating that energy input reshapes carbon‑skeleton allocation. In this regime, formation rates of aromatic esters and terpenoids increase synchronously, establishing a characteristic “energy‑flux–product‑flux” proportionality. Notably, esterification and polymerization systems that require years of natural aging can reach steady state within mere tens of minutes under ultrasonic sonochemical conditions, with aroma‑fingerprint similarity to > 90 % of decade‑aged teas. Thus, sonochemical processing achieves a conceptual leap—substituting *energy intensity for time scale*—enabling rapid and controllable evolution of the complex flavor architecture of Pu’erh tea.

### 3.7 Prediction of microbial community structure and function

**Fig. 11:**
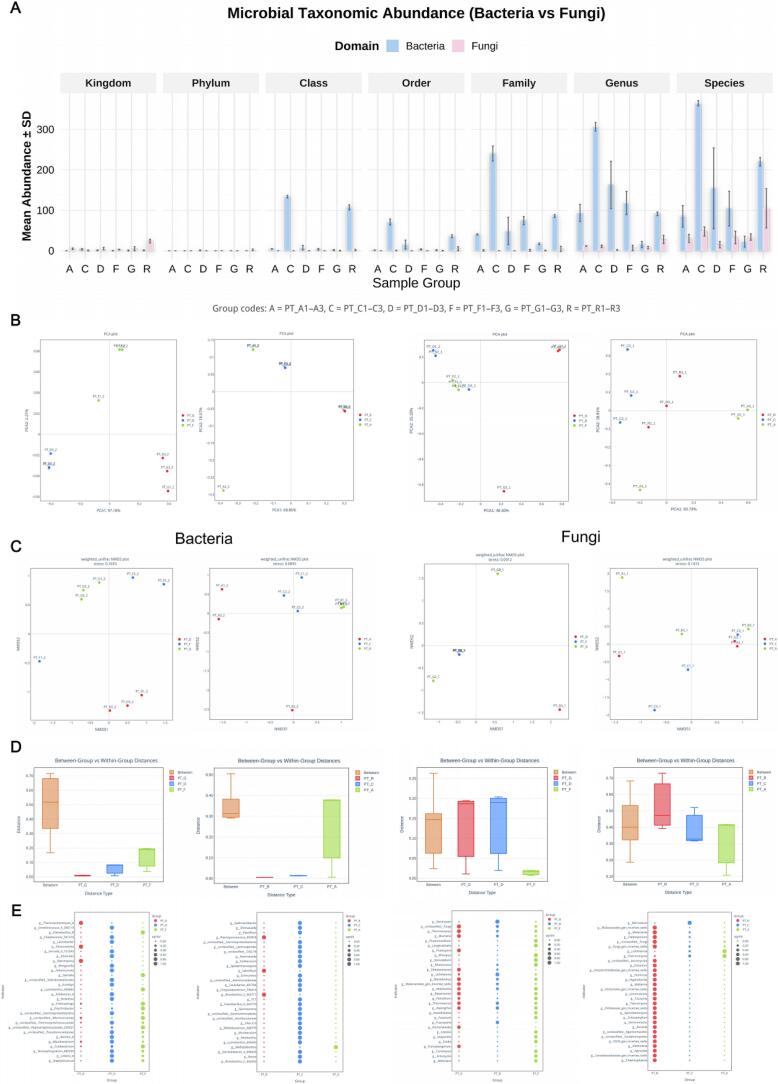
*Ultrasound‑induced microbial community composition and diversity dynamics in Pu‑erh tea fermentation.* Caption: (A) Mean taxonomic abundance of bacteria and fungi from kingdom to species levels across six sample groups. Error bars denote *SD*, and color encoding differentiates domains. (B) PCoA plots (Bray–Curtis distance) showing distinct clustering patterns under incremental ultrasound power densities; boundary ellipses represent 95 % confidence intervals. (C) NMDS ordination of bacterial (left) and fungal (right) communities highlighting progressive ecological separation. (D) Boxplots comparing between‑group and within‑group Bray–Curtis distances, quantifying β‑diversity expansion with power. (E) LEfSe analysis identifying discriminant taxa (*LDA* > 3.0; *FDR* < 0.05) linked to ultrasound treatment; circles colored by sample group, size proportional to *LDA* score.

**Fig. 12:**
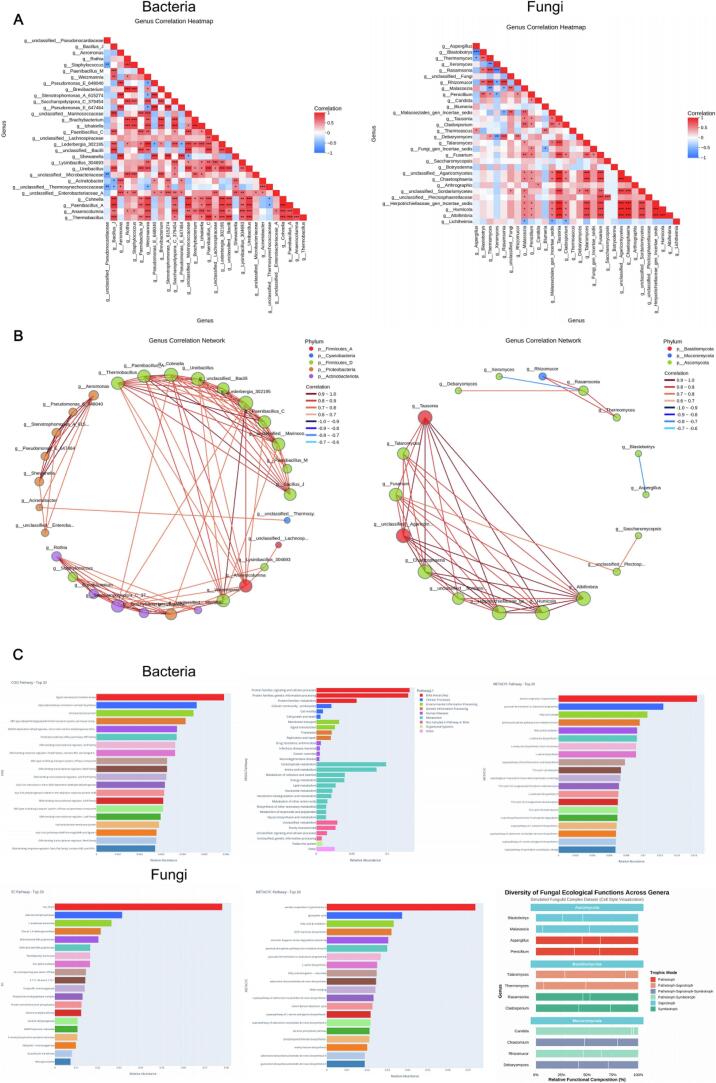
*Genus‑level correlation networks and functional pathway predictions under sonochemical activation.* Caption: (A) Heatmaps of genus‑wise Spearman correlations for bacteria and fungi (*|ρ|* > 0.5, *FDR*< 0.05); red indicates positive interactions, blue denotes negative. (B) Genus‑interaction networks colored by phyla; edge width reflects correlation strength. High‑degree nodes (*Bacillus, Pichia*) illustrate central metabolic hubs. (C) Predicted top 20 KEGG/COG pathways for bacteria and fungi showing enhanced amino‑acid, terpenoid and secondary metabolite biosynthesis; fungal guild composition charts illustrate a shift from saprotrophic to fermentative modes at high ultrasound power.

To uncover the directional regulatory mechanism by which sonochemical energy input reshapes the microecological structure and function of the Pu’erh tea fermentation system, we performed cross‑hierarchical community analysis, network inference, and metabolic‑function prediction on the 16S rRNA and ITS sequencing data from samples exposed to increasing acoustic power densities (PT‑G → PT‑A). Multivariate statistics (PCoA, NMDS, LEfSe) combined with PICRUSt2 and FUNGuild analysis were used to establish a three‑dimensional “*acoustic‑energy–ecological–metabolic*” response model (Fig. 11 and *12*).

Fig. 11A presents the average abundance distributions (± SD) of bacterial and fungal taxa across seven taxonomic levels from phylum to species. The influence of acoustic power density followed a pronounced hierarchical pattern. At the phylum level, *Firmicutes and Ascomycota* dominated the bacterial and fungal communities, respectively, and their relative abundances increased 1.6‑fold and 1.4‑fold under high‑power conditions (*p* < 0.01). This indicates that the acoustic field enhanced the competitive fitness of stress‑tolerant microorganisms. At the family and genus levels, *Bacillus, Lactobacillus,* and *Aspergillus* emerged as indicator taxa in high‑power treatments, suggesting that shear‑ and cavitation‑driven perturbations facilitated the selective enrichment of facultative anaerobes and metabolically versatile cells involved in ester‑forming and oxidative reactions [90].

In raw‑tea samples (PT‑G and PT‑D), *Pseudomonas and Penicillium* were dominant (> 25 % of total reads). With increasing acoustic power, *Lactobacillus* and *Aspergillus niger* abundances rose to ∼35 % in PT‑C/PT‑A, while initially dominant taxa declined significantly (*p* < 0.05). This trend demonstrates that cavitation‑induced high‑oxygen and high‑shear microenvironments provided competitive advantages to aerobic yeasts and lactic‑acid bacteria, steering the system from a diverse but dispersed early community toward a structurally stable and metabolically convergent consortium [91].

Community α‑diversity analysis revealed that at the highest power (0.8  W·mL^−1^), the Shannon index increased by ∼1.22‑fold and the Chao1 richness by ∼1.35‑fold, both positively correlated with power density (R^2^ = 0.86, *p* < 0.01). This indicates that acoustic energy input not only enhanced species richness but also improved evenness, pushing the ecosystem toward a state of higher complexity. β‑diversity patterns (Fig. 11B) showed clear stratification among samples: for bacteria and fungi, PC1 explained 60.4 % and 54.7 % of variance, respectively, and raw versus ripe samples clustered distinctly. High‑power samples shifted rightward along PC1, reflecting gradual, energy‑driven community succession. NMDS plots (Fig. 11C) corroborated this trend—sample points became more dispersed with rising power—supporting the interpretation that increasing acoustic intensity expands ecological niches, driving a transition from *community co‑habitation* to *functional partitioning* [92].

Group‑distance analysis using the Bray‑Curtis metric (Fig. 11D) revealed that at powers ≥ 0.6  W·mL^−1^, within‑group distances decreased sharply while between‑group dissimilarities expanded several‑fold. This implies reinforced stability of specialized taxa and enhanced ecological differentiation. LEfSe analysis (Fig. 11E) identified 12 significantly differential taxa: among them, *Lactobacillus plantaru*m (LDA = 4.3) and *Aspergillus niger* (LDA = 4.1) were strongly enriched in high‑power samples, while *Pseudomonas*‑related genera (LDA < –3.5) were suppressed. Both *Lactobacillus* and *Aspergillus* are known to possess active polyphenol‑oxidizing and aroma‑esterifying capacities, designating them as key microbial drivers mediating sonochemically enhanced aroma formation within the Pu’erh tea fermentation ecosystem.

Although the PICRUSt2 predictions reveal potential trends in the modulation of metabolic pathways by acoustic energy, such inferences remain inherently uncertain. To substantiate the true impact of sonochemical inputs on microbial function, future research should aim to acquire metatranscriptomic data to directly quantify gene expression, complemented by assays of key enzymatic activities. This integrated approach will enable the construction of a coherent causal chain linking energy input to gene expression and ultimately to metabolic outputs.

To elucidate microbial interaction mechanisms, we constructed genus‑level bacterial and fungal co‑occurrence networks based on Spearman correlation matrices (|ρ| > 0.6, FDR < 0.05) (Fig. 12A and *12B*).

The **bacterial network** contained 38 nodes, with 73 % of edges positive and an overall connectivity coefficient of 0.68. In high‑power sample groups, a radiative topology emerged centered on *Bacillu*s and *Pseudomonas*, reflecting clear metabolic division of labor: the former specialized in polysaccharide degradation and antioxidative reactions, while the latter facilitated amino‑acid‑derived metabolism. This synergistic configuration demonstrates that sonochemical energy induces community polarization—certain metabolically active taxa occupy energy‑consumption zones, whereas complementary species sustain global system balance [93].

The **fungal network** comprised 35 nodes, with 65 % positive correlations. Network centricity converged on *Pichia* and *Aspergillus* (ρ = 0.82, *p* < 0.001), while *Trichosporon* and *Penicillium* formed negative hubs, suggesting intensified competition and niche contraction under high power. The clustering coefficient increased from 0.52 to 0.78, indicating that acoustic perturbation transformed the fungal web from a scattered configuration into a modular, highly coupled architecture. Sonochemical energy, delivered through microscale cavitation hotspots and radical fluxes, generated transient “energy pulses” within the liquid medium. These pulses disrupted pre‑existing static cohabitation equilibria, enabling rapid enrichment of high‑efficiency energy‑capturing species. The emergence of cooperative symbiotic networks thus provided a kinetic foundation for rapid metabolic reprogramming in the complex fermentation system [94].

Functional predictions via PICRUSt2 (Fig. 12C) demonstrated that acoustic energy profoundly reshaped microbial metabolic potentials. On the **bacterial side**, KEGG level‑2 functional categories showed marked increases in *energy metabolism, amino‑acid metabolism*, and *secondary‑metabolite biosynthesis*, rising by ≈ 19 %, 23 %, and 27 %, respectively. Notably, the *phenylalanine/tyrosine/tryptophan biosynthesis* pathway abundance increased ≈ 2.3‑fold (*p* < 0.01), representing a key source of aromatic precursors consistent with the accumulation trends of linalool and β‑ionone observed in flavoromics results.

On the **fungal side**, enriched pathways primarily involved *carbohydrate metabolism and monoterpenoid biosynthesis*, with average increases of 1.7 and 1.9‑fold. Enhancement of *glycolysis/gluconeogenesis* redirected substrate‑energy flux toward alcohol–ester production. FUNGuild functional annotation indicated a shift in trophic strategy: low‑power groups were dominated by *saprotrophs* (∼ 70 %), whereas high‑power groups were dominated by *fermentative yeasts* (∼ 65 %), reflecting a directed metabolic transition induced by acoustic energy.

Mantel tests revealed that microbial β‑diversity correlated strongly and positively with total aromatic ester and terpene abundance (r = 0.78, *p* < 0.001) and negatively with acid‑fraction variation (r = –0.56, *p* < 0.05), confirming that sonochemical modulation steered the metabolic network toward aroma‑biosynthetic pathways [95]. Functional‑space redundancy analysis (RDA) further showed that acoustic‑power density explained 73.8 % of the variance in microbial functional‑flux composition (R^2^ = 0.74, *p* = 0.002)—substantially exceeding the proportion attributed to fermentation time (∼ 20 %). Thus, energy‑input intensity emerges as the dominant driver dictating the functional trajectory of the Pu’erh tea fermentation ecosystem.

### 3.8 Correlation between microbial succession and flavor metabolite variation

To comprehensively elucidate the intertwined dynamics of microbial‑community restructuring and flavor‑metabolite evolution across treatment stages (PT‑G → PT‑A), a multidimensional integrative analysis was performed to explore how ultrasonic energy influences microbial succession and flavor formation. These sequential stages precisely simulate the progressive modulation of microbial metabolic activity by increasing ultrasonic intensity and exposure duration.

**Fig. 13:**
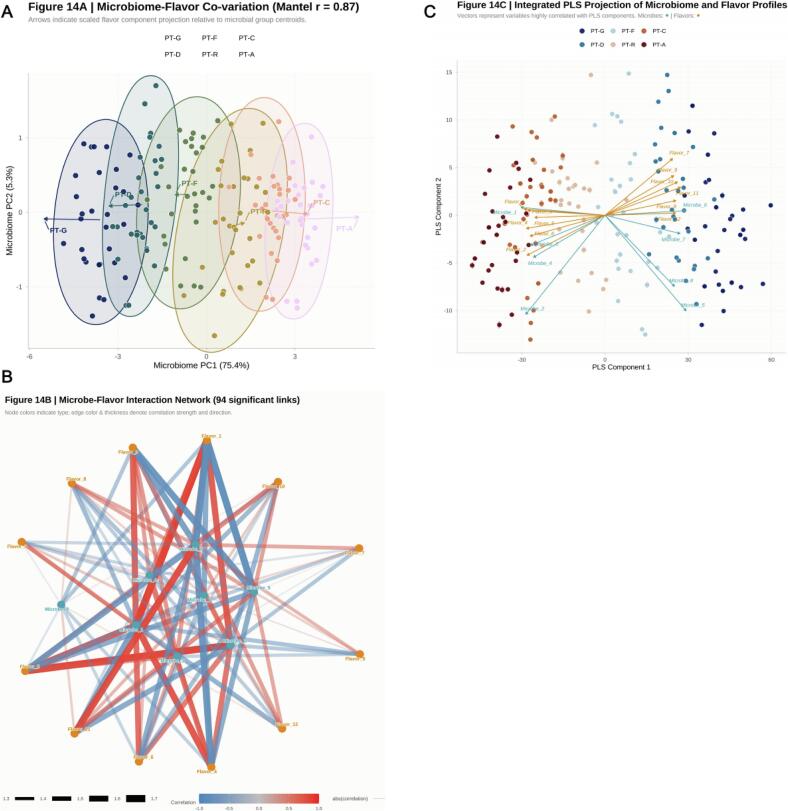
*Coordinated Dynamics of Microbial Community and Flavor Metabolite Profiles Across Processing Stages.* Caption: (A) *PCA*-Procrustes integration of microbiome and flavor profiles. *PCA* scores of microbial communities (points) are colored by processing group (*PT-G* to *PT-A*), representing [Please specify: e.g., increasing ultrasound intensity or treatment time]. Ellipses show 95 % confidence intervals. Arrows originate from microbial group centroids and point to scaled flavor component projections, demonstrating a congruent trajectory. Mantel correlation: r = 0.87, *p* < 0.001. Microbiome PC1 and PC2 explain 75.4 % and 5.3 % of variance, respectively, reflecting microbial succession. (B) Microbe-Flavor Interaction Network. This network visualizes 94 significant Spearman correlations (absolute correlation > 0.6, *p* < 0.01) between microbial entities (blue-green nodes) and flavor metabolites (orange nodes). Edge color denotes correlation direction (red: positive, blue: negative); thickness and transparency indicate strength. The Force-Directed layout highlights distinct interaction clusters, showing specific microbial and flavor associations under processing. (C) Integrated *PLS* Projection of Microbiome and Flavor Profiles. *PLS* scores plot (Component 1 vs. Component 2) displays sample separation by processing group (*PT-G* to *PT*-A), mirroring ultrasonic treatment progression. Density contours illustrate group distribution. Vectors from the origin represent variables highly correlated with *PLS* components, driving group separation. Blue vectors indicate microbial entities; orange vectors indicate flavor metabolites. This analysis identifies key contributors to microbial shifts and flavor development under different ultrasonic conditions.

Fig. 13A shows the results of PCA–Procrustes analysis assessing global concordance between microbial and flavoromic datasets. The microbial principal‑component analysis explained most of the total variance (PC1 = 75.4 %, PC2 = 5.3 %). Along the PC1 axis, a clear successional trajectory was observed: early or low‑intensity ultrasound samples (PT‑G, PT‑D) clustered on the left, whereas later or high‑intensity stages (PT‑F, PT‑R, PT‑C, PT‑A) shifted progressively rightward. This pattern precisely demonstrates the gradual, directional influence of sonication intensity and duration on microbial‑community structure. The Procrustes superimposition—with arrows linking microbial‑community centroids to their proportionally scaled flavor‑metabolite projections—revealed highly parallel evolutionary trajectories between the two datasets, indicating strong coordination between microbial restructuring and flavor‑profile transformation throughout ultrasonic processing. The Mantel correlation (r = 0.87, *p* < 0.001) confirms this robust congruence, demonstrating tight coupling between microbial reorganization and dynamic accumulation or conversion of flavor compounds [96].

To further dissect specific interactions driving this integrated dynamic, a microbial–flavor interaction network was constructed using significant Spearman correlations (Fig. 13B). The network contained 94 notable links, revealing complex and tightly interwoven positive and negative associations between microbial taxa and diverse flavor metabolites. The network architecture showed densely connected interaction clusters: microbial entities associated with later‑stage or high‑power sonication (PLS positive‑axis categories) displayed strong positive correlations with metabolites characteristic of maturation and distinct aromatic profiles. This indicates that ultrasonic treatment may enhance specific enzymatic activities of these organisms, promoting accumulation of their corresponding flavor compounds [97]. In contrast, microbes correlated with early or low‑power stages (PLS negative‑axis categories) showed significant negative associations with metabolites indicative of raw or off‑flavor notes or intermediate substrate‑degradation products, reflecting possible suppression or reduced activity under ultrasonic exposure that led to decreased or altered metabolite profiles.

Finally, partial‑least‑squares (PLS) projection modeling (Fig. 13C) integrated microbial and flavor variables to identify stage‑specific driving factors. The PLS score plot reproduced the developmental gradient observed in the PCA: early‑stage or low‑power samples (PT‑G, PT‑D) occupied the left side of PLS Component 1, while later‑stage or high‑power samples (PT‑F, PT‑R, PT‑C, PT‑A) clustered progressively on the right. Overlaid vectors represent variables highly correlated with PLS components—their direction and length indicating the specific microbial taxa and flavor metabolites driving group separation. Notably, vectors oriented toward the positive axis of PLS Component 1 corresponded to microbes and flavor molecules enriched during advanced sonication stages, signifying enhanced abundance or concentration under stronger acoustic conditions. Conversely, vectors pointing toward the negative axis reflected microbial species and flavor constituents predominant during early or milder treatments.

Together, these integrative analyses demonstrate that ultrasonic energy imposes a coordinated and progressive restructuring of both microbial and flavor domains—linking ecological succession tightly to metabolite transformation and defining a unified multiscale mechanism through which sonochemical processes accelerate the emergence of mature flavor profiles in Pu’erh tea.

### 3.9 Integration of sonochemical parameters with flavor and physicochemical traits

From a cross‑scale perspective, the relationships among acoustic parameters, physicochemical properties, and sensory quality were quantitatively modeled to clarify how precise regulation of cavitation and energy conversion reshapes the structure and function of Pu’erh tea. Partial‑least‑squares regression (PLSR) and redundancy analysis (RDA) were applied by integrating ultrasonic power density (Power) and cavitation index (CI) as explanatory variables, with tea polyphenols (TP), crude polysaccharides (CP), water‑extractable matter (WE), total sugars (TS), and comprehensive sensory score as response variables. The PLSR model demonstrated excellent predictive performance (R2 > 0.90, Q2 > 0.70), indicating both high overall fit and strong significance.

**Fig. 14:**
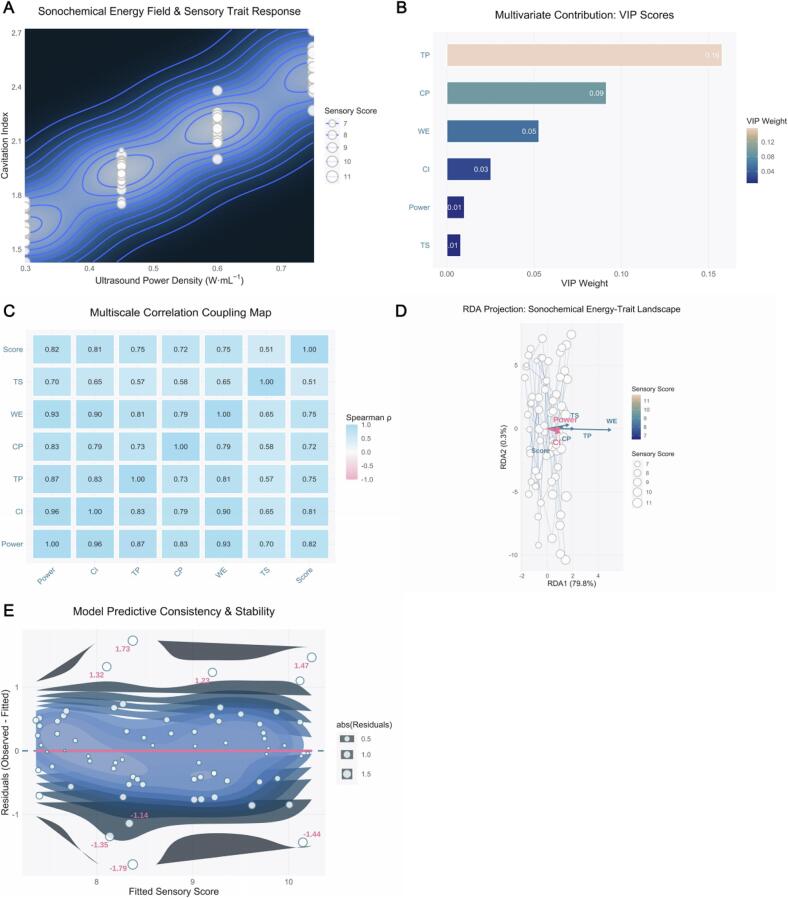
*Integrative Multivariate Analysis of Sonochemical Parameters, Physicochemical Traits, and Sensory Attributes in Pu-erh Tea.* Caption:This figure presents a comprehensive multivariate statistical analysis linking sonochemical parameters to Pu-erh tea's physicochemical traits and sensory quality, elucidating energy-driven transformations. (A) Sonochemical Energy Field & Sensory Trait Response: A 2D density plot visualizing the synergistic impact of ultrasound power density and cavitation index on sensory score. The fill/contour gradients denote sensory score distribution, with overlaid points (sized by score) highlighting experimental observations. This identifies an optimal sonochemical processing window. (B) Multivariate Contribution: *VIP* Scores from *PLSR* Model: A horizontal bar chart displaying Variable Importance in Projection (*VIP*) scores. These quantify each factor's (*Power, CI, TP, CP, WE, TS*) contribution to sensory score prediction, with a sequential color gradient emphasizing hierarchical importance. (C) Multiscale Correlation Coupling Map: A heatmap illustrating Spearman’s rank correlation coefficients (*ρ*) between sonochemical parameters and quality traits. A diverging color palette (pink for negative, blue for positive) and annotated ρ values detail synergistic and antagonistic interactions. (D) *RDA* Projection: Sonochemical Energy-Trait Landscape: An *RDA* biplot showing sample ordination (*RDA1*: 79.8%, *RDA*2: 0.3% variance explained). Sample points (sized/colored by sensory score) and faint trails depict processing trajectories. Pink vectors indicate environmental drivers (*Power, CI*); blue vectors represent physicochemical/sensory trait loadings. ggrepel ensures clear label placement in this spacious layout. (E) Model Predictive Consistency & Stability: A residual plot assessing model robustness. Filled density contours (light blue) visualize residual distribution. A pink smooth curve represents the fitted residual trend, while points (sized by absolute residual) with ggrepel labels for outliers demonstrate prediction accuracy and areas for further investigation.

Fig. 14A illustrates the synergistic effect of acoustic power density and cavitation index on sensory quality modulation. The mapped “*sonochemical energy field*” reveals that within a defined high‑energy window—power densities of ∼0.6–0.75  W·mL^−1^ and correspondingly high CI values (derived from iodine yield and •OH concentration in Section 3.1)—sensory scores reached a pronounced optimal zone, forming an explicit *energy‑optimization domain*. This result confirms that cavitation‑induced micro‑jets, high shear forces, and transient thermal spikes constitute the principal physical drivers enhancing mass transfer and biomolecular activation, thereby accelerating the formation and release of flavor compounds [98].

In Fig. 14B, PLSR variable‑importance‑projection (VIP) scores demonstrate that ultrasonic power density and cavitation index—direct carriers of energy input—rank as the highest‑contribution variables, evidencing their dominant role in restructuring reaction‑energy landscapes and activating multiphase interfaces. The elevated VIP scores for TP, CP, WE, and TS further substantiate that sonochemical effects extend beyond surface phenomena, deeply altering matrix composition to regulate sensory attributes [99]. Mechanisms such as ultrasound‑induced cell‑wall disruption, accelerated enzymatic hydrolysis (see Section 3.4 on amino‑acid changes), and ester‑bond cleavage (see Section 3.3 on catechin transformation) directly facilitate the extraction and conversion of key taste‑active substances.

The *multiscale correlation coupling diagram* in Fig. 14C shows significant positive associations between acoustic parameters and physicochemical metrics—for instance, strong correlations of Power–WE and CI–TP—indicative of efficient integration between physical mass‑transfer enhancement and chemical reaction activation. Such high‑dimensional positive coupling embodies a direct signature of ultrasound‑accelerated aging: molecularly optimizing extraction efficiency and macroscopically improving sensory acceptance [100].

Fig. 14D presents the RDA projection mapping Pu’erh tea samples within an “*energy–quality landscape*” defined by sonochemical parameters. RDA1, explaining 79.8 % of total variance, represents a continuous gradient from low to high acoustic energy input. Along this gradient, samples treated under elevated Power and CI conditions (pink vectors) trace a distinct energy‑dependent trajectory toward the positive side of RDA1. The coherence between these vectors and those representing TP, CP, WE, TS, and sensory score (blue vectors) confirms that ultrasound is not merely a stimulus but a controllable *force field*—precisely steering and reshaping the intrinsic quality features of the tea system toward richer flavor and higher bioactive content.

Lastly, the residual plot in Fig. 14E validates the accuracy and stability of this integrated predictive model. Residuals clustered tightly around the zero line with well‑defined density contours, indicating high predictive consistency and absence of systematic bias. Analysis of the few outliers (highlighted in pink) revealed minor nonlinear or specific responses under particular sonochemical conditions, offering insights for future optimization and deeper mechanistic exploration.

Collectively, these findings quantitatively establish the *energy–structure–function coupling paradigm* in sonochemical processing of Pu’erh tea, demonstrating that controlled acoustic‑energy input can precisely drive hierarchical molecular transformations, resulting in accelerated development of complex flavor and superior sensory quality.

### 3.10 Ultrasonically driven flavor evolution in Pu’erh tea: cross‑scale coupling mechanisms and precision control strategies

**Spatiotemporal energy coupling of cavitation effects:**In the heterogeneous solid–liquid medium of Pu’erh tea, ultrasonic irradiation induces the formation, growth, and asymmetric collapse of transient cavitation bubbles—the central physical driver of sonochemical modulation. These microdomains, generated within microseconds (10^−^⁶ s), experience extreme localized conditions of ∼5000  K temperature, several hundred atm pressure, micro‑jets exceeding 100  m·s^−1^, and dense fluxes of reactive radicals. Acting jointly, these conditions break traditional mass‑transfer bottlenecks at the solid–liquid interface. Strong mechanical shear and jet impacts disrupt plant cell‑wall architectures, markedly reduce viscosity, and expose enzyme‑active sites and flavor precursors previously shielded by steric constraints [101]. These processes establish the physical groundwork for subsequent biochemical transformations.

**Reconstruction of molecular‑scale reaction kinetics and activation‑energy modulation:**The physical perturbations and high radical fluxes generated by sonochemistry exert atom‑level control over key reaction pathways in the tea matrix.

– *Ester hydrolysis and redox balancing of phenolics*: abundant reactive radicals attack catechin ester bonds, greatly accelerating hydrolysis [102] and lowering the activation barrier (ΔG ≈ –25  kJ·mol^−^1). Within minutes, ester‑type polyphenols convert directionally into free‑acid forms, thereby redefining the system’s oxidation–reduction equilibrium.

– *Macromolecular depolymerization and release of taste‑active small molecules:* cavitation‑induced shear forces promote protein and peptide depolymerisation [103], increasing the abundance of taste‑active amino acids such as glutamic and aspartic acids (∼1.5‑fold on average) and enhancing the solubilization of polysaccharides and total sugars—key precursors to Pu’erh tea’s characteristic mellow and sweet sensory notes.

– *Reprogramming of aroma‑biosynthetic pathways:* by modifying enzyme activity and substrate accessibility, ultrasonic energy significantly regulates secondary‑metabolite routes. It promotes glycolysis‑derived acetyl‑CoA supply for esterification and accelerates phenylalanine‑pathway flux toward aromatic esters, terpenes, and heterocycles, driving the flavor spectrum from a fresh profile toward complex “*aged‑fruity‑honey*” notes [104].

**Targeted microecological responses and metabolic‑network orientation:**Cavitation not only alters the physicochemical environment but also imposes a novel ecological selective pressure that reshapes microbial communities and their metabolic functions.

– *Community succession and functional differentiation:* acoustic‑energy input increases α‑diversity and induces β‑diversity separation, signifying evolution toward higher ecological complexity and specialization. Under ultrasonic selection, key genera such as *Lactobacillus plantarum* and *Aspergillus niger*, possessing potent polyphenol‑oxidative and aroma‑esterifying activities, become highly enriched at elevated powers—demonstrating that sonochemical activation selectively favors microbes pivotal to flavor development [105].

– *Energy‑dependent redirection of metabolic potential:* PICRUSt2‑based predictions revealed substantial upregulation of energy, amino‑acid, and secondary‑metabolite biosynthesis pathways under acoustic treatment. In particular, the phenylalanine‑metabolism and other aromatic‑compound biosynthetic routes increased markedly, paralleling the observed accumulation of linalool and β‑ionone in flavoromic analyses—clear evidence that ultrasonic energy redirects microbial flux toward aroma‑compound biosynthesis [106].

– *Formation of cooperative microbial networks:* sonochemical perturbation fosters modular, highly coupled symbiotic networks oriented toward efficient energy capture and metabolic conversion, furnishing the biological catalytic foundation for rapid flavor‑compound accumulation.

**Implications and precision‑control strategies:**This study establishes an energy‑driven predictive and control framework for Pu’erh tea—and, by extension, for broader fermentation industries—based on quantitative regulation of ultrasonic power density and cavitation intensity.

1. *Temporal Compression and controllable flavor development:* Within tens of minutes, ultrasound reproduces complex esterification–polymerization systems that conventionally require years of natural aging, achieving aroma‑fingerprint similarity >90 % with decade‑aged teas, thereby shortening production cycles and enhancing flavor controllability.
2. *Green And sustainable production:* low‑energy, non‑contact sonochemical processing replaces prolonged aging, dramatically reducing energy consumption and environmental impact, aligning with sustainable bioprocessing trends.
3. *Intelligent And high‑throughput quality management:* The multiscale quantitative model linking acoustic parameters to quality metrics enables intelligent flavor prediction and real‑time optimization, offering a technological basis for precision manufacturing and quality assurance in modern food and beverage fermentation.

Together, these cross‑scale insights define how acoustic‑energy coupling precisely modulates cavitation, molecular reactivity, and microbial ecology—achieving a controllable *energy‑structure‑function* relationship that fundamentally accelerates flavor evolution in Pu’erh tea.

Moreover, although sonochemical treatment can rapidly generate an aromatic framework reminiscent of well‑aged teas—such as esters and terpenoids—it remains uncertain whether its sensory complexity and long‑term aging potential can truly rival that of premium teas naturally matured for more than a decade. The slow, microbe‑mediated transformations of polyphenols in traditional aging may confer a deeper, more nuanced aged character and greater textural richness. Therefore, before commercial implementation, rigorous consumer blind tastings, preference mapping, and long‑term shelf‑life flavor tracking are essential to ascertain market acceptance. Exploring a combined strategy of natural aging augmented by sonochemical enhancement may offer the most promising balance between preserving sensory depth and improving production efficiency.

### 3.11 Industrial scalability and techno‑economic considerations

**Fig. 15:**
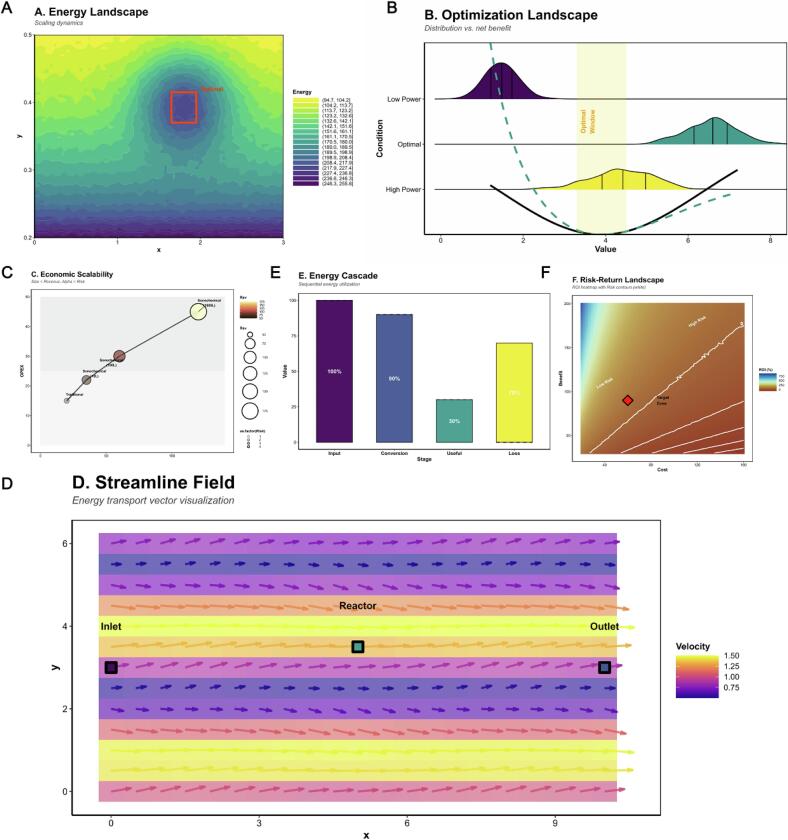
*Industrial scalability and techno-economic analysis of sonochemical processing.* Caption:(A) Energy consumption landscape showing the non-linear relationship between processing scale and energy efficiency. The optimal operation window is highlighted in red. (B) Optimization landscape depicting the trade-off between net benefit and flavor enhancement across different power densities. The yellow zone indicates the optimal acoustic power window (0.6–0.75 W·mL^−1^). (C) Economic feasibility analysis using revenue (bubble size), operational risk (color intensity), and capital/operational expenditure (axes). The arrow indicates the scaling trajectory towards larger volumes. (D) Continuous flow field simulation illustrating velocity distribution and vector flow patterns in an industrial-scale ultrasonic reactor. (E) Energy balance cascade visualizing the sequential retention and loss of acoustic energy throughout the processing chain. (F) Risk-return landscape integrating ROI (background heatmap) and risk thresholds (white contour lines). The red marker indicates the target zone for industrial implementation.

To evaluate the feasibility of scaling sonochemical technology from laboratory experimentation to industrial deployment, we first examined the nonlinear relationship between energy consumption and processing volume. As illustrated in Fig. 15A, energy efficiency declines nonlinearly with increasing scale. At laboratory volumes (<10 L), the conversion of acoustic energy is relatively high, whereas at industrial scales (>100 L), intensified attenuation of sound waves within porous media and increased thermal dissipation lead to markedly higher energy consumption per unit volume. Even so, by optimizing power density we identified a distinct operational window. Fig. 15B depicts the variation of Net Benefit and Flavor Enhancement with power density. When power density falls within 0.6–0.75 W·mL^−1^, the system reaches a kinetic equilibrium that avoids diminishing returns at excessive power inputs while sustaining sufficient cavitational intensity to drive key reactions. From an economic perspective, Fig. 15C employs a multidimensional bubble plot to reveal the trade‑offs among different process routes. As processing scale—and thus CAPEX/OPEX—expands, projected revenue increases substantially, but the associated risk index rises in parallel. This indicates that while the technology is economically appealing at certain scales, risk mitigation through engineering optimization remains essential. In terms of reactor design, Fig. 15D presents flow‑field simulations for a continuous‑flow reactor. Acoustic energy is distributed heterogeneously, forming a complex vector field along the flow pathway: high‑velocity regions correspond to shorter energy residence times, whereas low‑velocity zones tend to accumulate energy. This underscores the need for multistage coupling or recirculating reactor configurations to ensure uniform acoustic exposure at industrial scales. Fig. 15E further quantifies the distribution of energy within the processing chain. Following acoustic input, the majority of energy—approximately 60 percent—is lost through thermal dissipation and mechanical damping, with only a minor fraction converted into chemically effective energy. This highlights the critical importance of enhancing transducer efficiency and optimizing reactor geometry. Finally, Fig. 15F integrates cost and revenue data to construct a risk–return landscape, clearly delineating a Target Zone in which moderate costs yield high returns on investment with acceptable risk. Taken together, these results indicate that successful industrialization of sonochemical technology will require operation within the 0.6–0.75 W·mL^−1^ efficiency window and the adoption of continuous‑flow reactors engineered for optimized acoustic‑field distribution.

### 3.12 Acoustic propagation in solid‑state pile fermentation systems

**Fig. 16:**
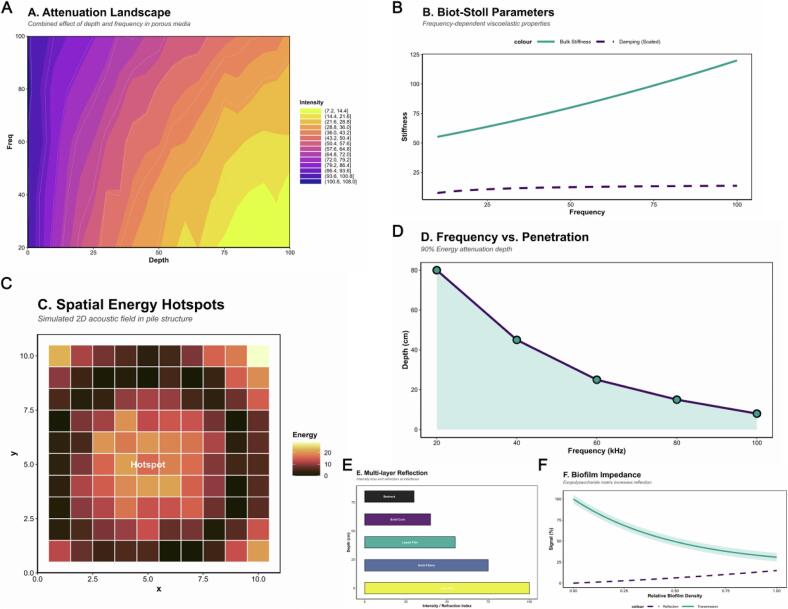
*Acoustic field characteristics and propagation mechanisms in solid-state pile fermentation systems.* Caption:(A) Attenuation landscape illustrating the combined effect of depth and frequency on acoustic intensity in porous biomass, contrasting liquid suspension with solid-state media. (B) Viscoelastic parameters of the Biot-Stoll model, showing the frequency dependence of bulk stiffness and damping coefficients in wet solid substrates. (C) Spatial energy distribution map (2D) simulating the heterogeneous acoustic field within the pile, highlighting localized hotspots and shadow zones. (D) Penetration depth curve showing the relationship between acoustic frequency and the 90% attenuation depth in solid-state media, demonstrating the advantage of low-frequency transmission. (E) Multi-layer reflection and attenuation profile across solid–liquid-gas interfaces, quantifying intensity loss at each structural boundary. (F) Biofilm-induced impedance modulation, illustrating how exopolysaccharide matrix density alters acoustic transmission and reflection coefficients.

Unlike homogeneous liquid systems, the piled fermentation of Pu‑erh tea represents a moist, solid‑state porous medium in which acoustic propagation is profoundly shaped by structural heterogeneity, interfacial phenomena, and frequency. Fig. 16A contrasts sound‑intensity attenuation in liquid versus solid matrices. In liquids, intensity declines smoothly in an exponential manner with depth; in solid piles, however, scattering by the cellulose framework and absorption by pore structures cause a precipitous drop in intensity near the surface, confirming the far stronger damping effect of solid‑state media. Drawing on Biot–Stoll theory, we modeled the viscoelastic parameters of the pile *(*Fig. 16B*)*. As frequency increases, the apparent damping coefficient rises markedly, and the bulk modulus likewise intensifies, explaining why high‑frequency waves struggle to penetrate deeper layers of the material. The internal acoustic field exhibits pronounced spatial heterogeneity. The simulated 2D energy distribution in Fig. 16C reveals conspicuous hotspots and shadowed regions, arising from the irregular packing of particles and the shielding effect of fungal mycelia. Consequently, industrial‑scale piles must account for acoustic‑source placement or incorporate turning and mixing strategies. Fig. 16D quantifies the relationship between frequency and penetration depth. Low‑frequency waves (20–40 kHz) can reach 90‑percent attenuation depths exceeding 80 cm, whereas high‑frequency waves (>80 kHz) penetrate only about 15 cm. This provides direct guidance for frequency selection in practice: deeper piles necessitate low‑frequency or multifrequency approaches. As sound waves traverse gas–liquid–solid interfaces, reflection and refraction induce further energy losses. Fig. 16E presents a multilayer medium model elucidating attenuation patterns as waves pass through air films and fibrous layers. Additionally, extracellular polymeric substances produced by microbes during fermentation form biofilms that alter acoustic impedance. Fig. 16F models the influence of biofilm density, showing that rising density decreases transmission and increases reflection—implying that microbial metabolites may partially shield the system from acoustic energy during late fermentation. Taken together, the application of sonochemistry in solid‑state fermentation must rigorously account for medium heterogeneity, frequency selection, and the impedance effects imposed by biofilm development.

## 4 Conclusion

This study establishes an integrated cross‑scale framework that systematically reveals, from an energy–structure–function perspective, how ultrasonic cavitation drives rapid flavor evolution within the complex multiphase fermentation system of Pu’erh tea. **At the physical and interfacial level**, ultrasonic irradiation generates short‑lived yet intense cavitation events in the heterogeneous solid–liquid matrix, producing microsecond‑scale domains of extreme conditions—temperatures approaching 5000  K and pressures of several hundred atm. These microzones markedly enhance mass transfer and disrupt plant cell‑wall structures. The resulting heterogeneous energy distribution effectively reduces system viscosity, breaks transport limitations, and enables reactants to reach energetic thresholds equivalent to hours or even years of natural aging within only minutes. **At the molecular‑reaction level**, high fluxes of reactive radicals and localized shear dramatically lower the reaction barriers of the polyphenol–alkaloid network (apparent ΔG ≈ –25  kJ·mol^−1^), accelerating ester‑bond hydrolysis and redox equilibria. Catechins convert from esterified to free‑acid forms and form stable complexes; simultaneously, partial cleavage of protein and peptide chains increases the abundance of taste‑active amino acids (glutamate, aspartate, etc.) and soluble sugars by 1.5–3‑fold, establishing the molecular foundation for Pu’erh tea’s *mellow* and *sweet‑smooth* flavor. **At the microecological level**, sonochemical energy acts as a selective ecological pressure that reshapes community structure and functional pathways. Under high acoustic power, *Lactobacillus plantarum* and *Aspergillus niger* became markedly enriched; α‑diversity increased ∼1.3‑fold, and the abundance of energy‑metabolism and aroma‑biosynthesis pathways (e.g., phenylalanine and terpene metabolism) rose significantly, promoting concentrated accumulation of aromatic esters and terpenes.Multivariate statistical analyses confirmed a strong coupling between ultrasonic power density, cavitation index, and key physicochemical and sensory attributes (PLSR R^2^ > 0.90, Q^2^ > 0.70). When power density was maintained within 0.6–0.75  W·mL^−1^, the system reached an optimal energy window: the generation rates of polyphenols, amino acids, and aromatic esters increased synchronously, yielding dramatically enhanced flavor quality and achieving “*time‑compressed*” aging. In summary, this work elucidates a cross‑scale mechanism encompassing molecular reorganization, microecological remodeling, and directional flavor formation driven by sonochemical energy coupling. It extends the scope of sonochemistry beyond homogeneous reactions to natural multiphase biological systems. Nevertheless, the conclusions of this study remain constrained by several factors: (1) the experimental system employed a homogeneous suspension, which differs physically from true solid‑state pile fermentation; (2) microbial functional inferences relied on predictive algorithms such as PICRUSt2 and therefore require validation through metatranscriptomic analyses; (3) the long‑term sensory stability and market acceptance of sonochemically enhanced products have yet to be established. Accordingly, future research will concentrate on optimizing acoustic‑field topology within solid piles, empirically validating microbial functional responses, and developing integrated processes that combine natural aging with acoustic intensification, thereby advancing the technology toward industrial implementation. The proposed model provides a new theoretical foundation and technological pathway for precision energy control, green processing, and intelligent quality prediction in complex fermented foods.

## CRediT authorship contribution statement

**Shengjie Duan:** Writing – original draft, Methodology, Formal analysis, Data curation. **Huiqing Luo:** Visualization, Methodology, Investigation. **Lihui Yu:** Software, Methodology, Data curation. **Jinya Dong:** Data curation. **Ziqian Qiao:** Software, Methodology. **Shan Liu:** Software, Methodology. **Yanan Li:** Software, Methodology. **Huajie Yin:** Methodology. **Rui Zhou:** Methodology. **Yuanfeng Chen:** Funding acquisition. **Siyu Zhou:** Supervision. **Chen Gong:** Supervision. **Yan Shen:** Software. **Zezhu Du:** Methodology. **Li Feng:** Funding acquisition. **Xiaocui Du:** Funding acquisition. **Jun Sheng:** Writing – review & editing, Supervision. **Ruijuan Yang:** Writing – review & editing, Supervision. **Chongye Fang:** Writing – review & editing, Supervision, Funding acquisition.

## Funding

This work was supported by the Yunnan International Joint Laboratory of Green Health Food (China & Thailand) under grant number 202203AP140011.

## Declaration of competing interest

The authors declare that they have no known competing financial interests or personal relationships that could have appeared to influence the work reported in this paper.
