# Supplementary material for: Multiscale mechanistic insights into sonochemical energy coupling and flavor evolution in Pu‑erh tea
Source: Ultrason Sonochem. 2026 Jan 1;125:107735. doi: 10.1016/j.ultsonch.2025.107735 (PMC12882671; doi:10.1016/j.ultsonch.2025.107735)
Supplement: Supplementary Data 1 [file mmc1.docx]

**Supplementary Figure Legends**

**Detailed Supplementary Figure Captions**

**Supplementary Figure 3.1A**

**Thermal response of Pu-erh tea extracts under varying acoustic power densities.**

Scatter plots show the average extraction temperature for six representative tea types (PT-G, PT-D, PT-F, PT-R, PT-C, PT-A) across acoustic power densities ranging from 0.3 to 0.8 W·mL^-1^ (n = 6 per group). Black regression line represents the overall linear fit, illustrating a significant positive correlation between temperature and power density. Error distribution and color coding correspond to individual tea types.

**Supplementary Figure 3.1B**

**Hydroxyl radical (OH) concentration trends as a function of acoustic power density.**

Violin plots depict OH radical concentrations (µM) for each tea type under different power densities. Overlaid jittered points indicate individual measurements, with a black linear fit showing the overall trend. Data reveal a marked increase in OH generation with rising acoustic input, consistent across all fermentation stages.

**Supplementary Figure 3.1C**

**Iodine yield as a measure of cavitation intensity in Pu-erh tea extracts.**

Scatter plots present iodine yield (µmol·L^-1^) for each tea type (color-coded), plotted against acoustic power density (0.3–0.8 W·mL^-1^). Black regression line shows a significant positive relationship, supporting that higher ultrasound power promotes stronger cavitation events and greater oxidative potential.

**Supplementary Figure 3.1D**

**Viscosity–mass transfer coefficient (kₘ) relationships under ultrasonic treatment.**

Scatter plots, faceted by tea type, show the relationship between extract viscosity (mPa·s) and normalized mass transfer coefficient under different power densities. Color gradient represents acoustic power level. Across all tea types, viscosity shows a clear negative association with kₘ, reflecting effective cavitation-induced microstreaming and shear forces.

**Supplementary Figure 3.1E**

**Nonlinear relationship between acoustic power density and mass transfer coefficient (kₘ).**

Scatter plots indicate kₘ values for individual tea types, with a second-order polynomial regression (black curve) capturing the nonlinear enhancement of mass transfer at higher power inputs. Supports empirical model predictions relating physical energy input to solute transport efficiency.


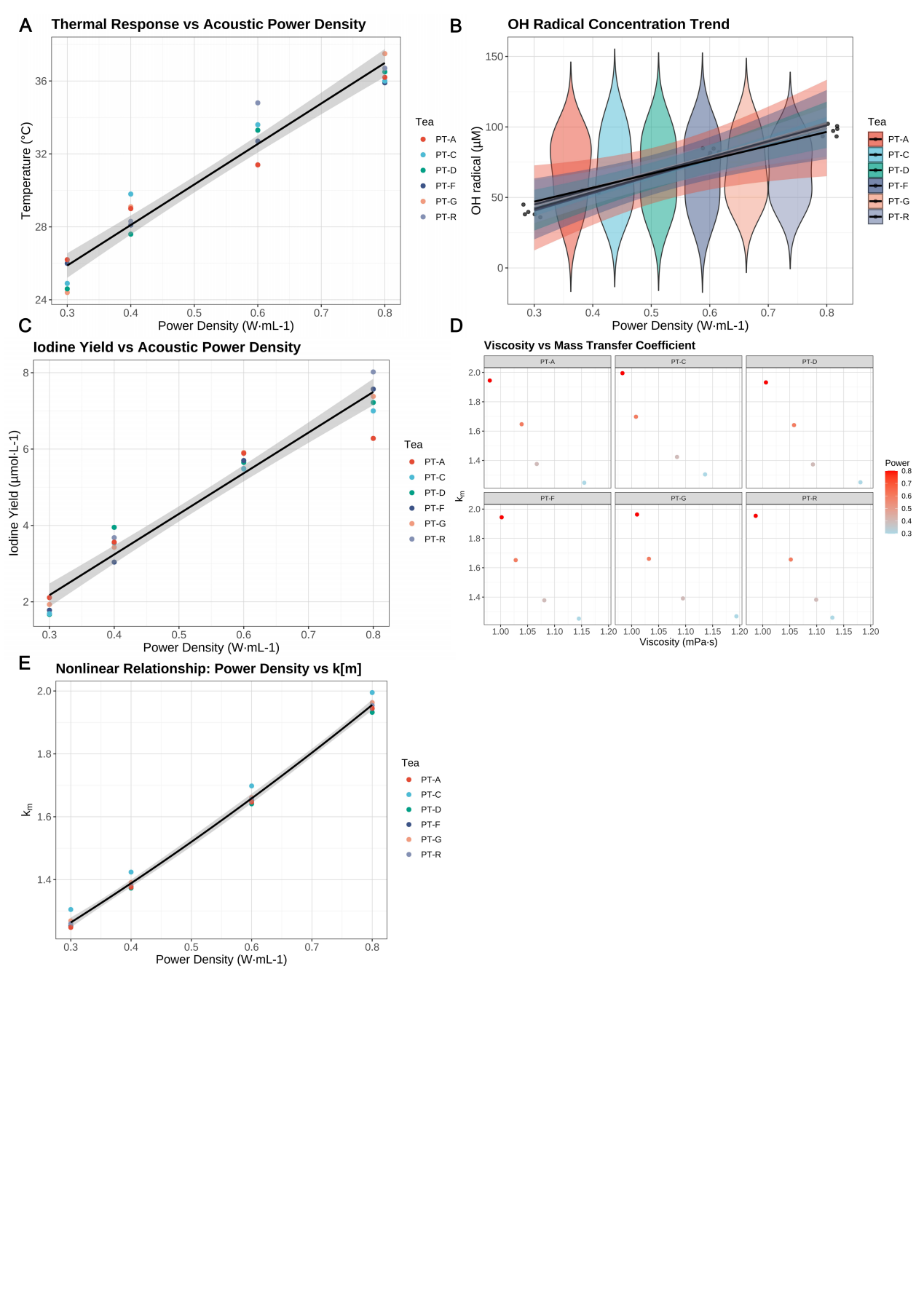


**Supplementary Table Legend**

**Detailed Supplementary Table Caption**

**Supplementary Table 3.1**

*Summary of acoustic field characteristics, cavitation dynamics, and mass transfer properties of Pu-erh tea extracts under varying acoustic power densities.*

Values represent mean ± standard deviation (n = 6 per power density). Measured parameters include extraction temperature (℃), energy absorption coefficient (η), iodine yield (µmol·L^-1^), hydroxyl radical concentration (µM), viscosity (mPa·s), and normalized mass transfer coefficient (kₘ).

| **Power** | **Temperature_mean** | **Temperature_sd** | **Eta_mean** | **Eta_sd** | **Iodine_mean** | **Iodine_sd** |
| --- | --- | --- | --- | --- | --- | --- |
| 0.3 | 25.1666666666667 | 0.750111102881878 | 0.608333333333333 | 0.0231660671385254 | 1.81166666666667 | 0.175318757315544 |
| 0.4 | 28.65 | 0.796868872525461 | 0.661666666666667 | 0.0116904519445001 | 3.53333333333333 | 0.299977776954672 |
| 0.6 | 33.2333333333333 | 1.12901136693422 | 0.716666666666667 | 0.00816496580927727 | 5.68666666666667 | 0.186619041543639 |
| 0.8 | 36.4666666666667 | 0.58878405775519 | 0.768333333333333 | 0.0263944438597722 | 7.245 | 0.58589248160392 |

Continuation of Table 3.1

| **OH_mean** | **OH_sd** | **Viscosity_mean** | **Viscosity_sd** | **km_mean** | **km_sd** |
| --- | --- | --- | --- | --- | --- |
| 40.2166666666667 | 3.78598291948956 | 1.15666666666667 | 0.0257578467008923 | 1.26433333333333 | 0.0212947567881548 |
| 55.7166666666667 | 1.64853470290033 | 1.0865 | 0.0117260393995586 | 1.38733333333333 | 0.0190122767354851 |
| 81.9333333333333 | 2.51369581824585 | 1.03616666666667 | 0.0179601410536406 | 1.65916666666667 | 0.0202525718531417 |
| 97.55 | 3.67246511215559 | 0.994666666666667 | 0.012863384728238 | 1.9555 | 0.0219704346793595 |
