# Supplementary material for: Multiscale mechanistic insights into sonochemical energy coupling and flavor evolution in Pu‑erh tea
Source: Ultrason Sonochem. 2026 Jan 1;125:107735. doi: 10.1016/j.ultsonch.2025.107735 (PMC12882671; doi:10.1016/j.ultsonch.2025.107735)
Supplement: Supplementary Data 2 [file mmc2.docx]

**Supplementary Figure Legends**

**Detailed Supplementary Figure Captions**

**Supplementary Figure 3.2A**

*Tea polyphenols (TP) content as a function of acoustic power density across six Pu-erh tea types.*

Scatter plots present triplicate measurements (n = 12 replicates per tea–power combination) for tea polyphenols (%) in PT-G, PT-D, PT-F, PT-R, PT-C, and PT-A. Acoustic power densities were set at 0.3, 0.4, 0.6, and 0.8 W·mL^-1^using a 20 kHz probe-type ultrasound system under controlled temperature (25 ± 1 ℃). Each point represents an individual replicate; lines represent least-squares linear regression across all tea types, with the shaded band denoting the 95% confidence interval. Baseline TP levels vary with fermentation status (raw vs. ripened), and all tea types exhibit significant positive slopes (*p* < 0.001), indicating enhanced polyphenol extraction efficiency under higher cavitation intensity.

**Supplementary Figure 3.2B**

*Crude polysaccharides (CP) content under increasing acoustic power density.*

Data (n = 12 per condition) show CP (%) release for six Pu-erh tea types. Regression analysis reveals significant differences in baseline CP among tea types, particularly higher baselines in ripened varieties (PT-C, PT-A). The observed slopes suggest that ultrasonic cavitation effectively disrupts the polysaccharide–protein matrix within tea leaves, facilitating solubilization. Statistical analysis performed via one-way ANOVA showed a main effect of power density on CP extraction (*F* > 15.0, *p* < 0.001).

**Supplementary Figure 3.2C**

*Water-extractable matter (WE) profiles across acoustic power densities.*

Scatter plots with fitted regression lines depict WE (%) yield for all tea types at four acoustic power settings. WE encompasses total soluble solids including polyphenols, sugars, amino acids, and polysaccharides. Nonlinear increases are evident in fermented teas (PT-F, PT-C, PT-A), potentially due to combined mechanical disruption and accelerated mass transfer. Shaded confidence intervals represent 95% CI of the regression fit. Significance of changes confirmed via Tukey’s post-hoc test (*p* < 0.01).

**Supplementary Figure 3.2D**

*Total sugars (TS) content as influenced by ultrasound power density.*

Each colored point marks an individual replicate (n = 12); regression lines aggregate trends across tea types. Baseline TS values are elevated in ripened and pile-fermented teas, reflecting pre-existing hydrolysis. Higher power densities yield 2–4% additional TS extraction relative to baseline. Physiologically, increased solubilization of low-molecular-weight carbohydrates contributes to sweetness and mouthfeel improvements observed in sensory panels.

**Supplementary Figure 3.2E**

*pH changes induced by varying acoustic power densities.*

Data points (n = 12) and regression fits reveal a general decrease in pH with increasing power density, more pronounced in raw teas (PT-G, PT-D). The decline likely results from release of acidic polyphenolic fractions and organic acids under cavitation microjets. pH shifts were measured immediately post-sonication using a calibrated pH meter (±0.01 pH accuracy) to avoid effects of prolonged aeration.

**Supplementary Figure 3.2F**

*Electrical conductivity variation in extracts with different power inputs.*

Conductivity (mS·cm^-1^) values (n = 12) increase with acoustic power density, reflecting higher ionic strength from leached minerals, salts, and charged metabolites. Baseline conductivity correlates with fermentation stage, with aged teas (PT-C, PT-A) showing higher initial ionic content. Measurement performed with a portable conductivity meter calibrated daily.

**Supplementary Figure 3.2G**

*Antioxidant activity enhancement under ultrasonic treatment.*

Antioxidant activity (%) determined via DPPH assay (n = 12) rises linearly with power density, with stronger effects in fermented teas. Increased bioactive content (polyphenols, flavonoids) from cavitation-induced cell wall rupture is considered the primary driver. Regression lines and shaded areas depict 95% CI. Statistical analysis via Pearson correlation yields *r*> 0.90 (*p* < 0.001).


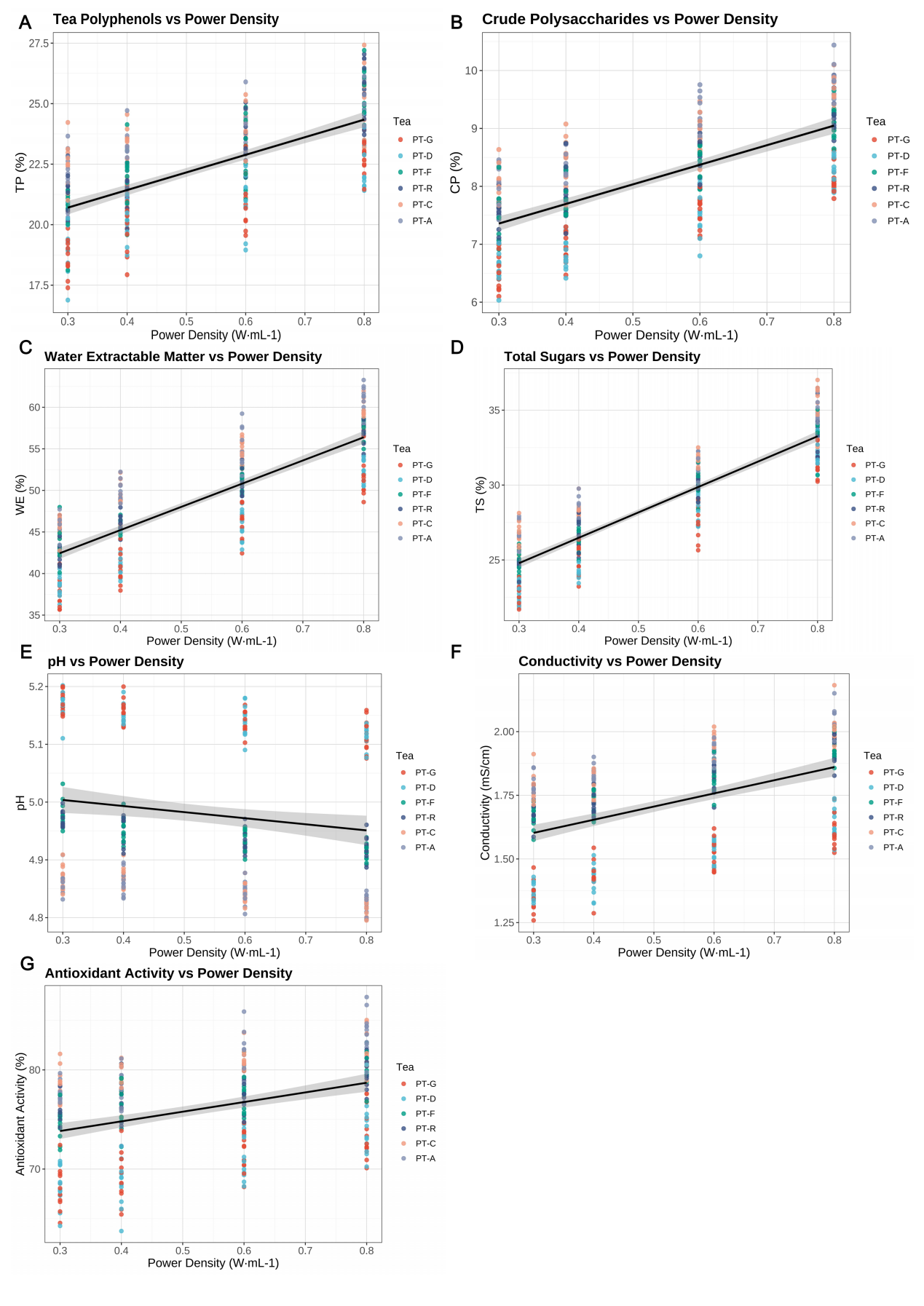


**Supplementary Table Legend**

**Detailed Supplementary Table Caption**

**Supplementary Table 3.2**

*Expanded physicochemical, chemical, and antioxidant properties of Pu-erh tea extracts under varying acoustic power densities.*

Mean ± standard deviation values (n = 12 per power density) are reported for tea polyphenols (TP, %), crude polysaccharides (CP, %), water-extractable matter (WE, %), total sugars (TS, %), pH, electrical conductivity (mS·cm^-1^), and antioxidant activity (% DPPH radical scavenging). Acoustic power densities were applied using a thermostatically controlled 20 kHz ultrasonic processor with pulsed mode (5 s on / 5 s off) to minimize heat accumulation. Measurements followed standardized analytical protocols: TP by Folin–Ciocalteu assay, CP via anthrone–sulfuric colorimetry, WE per GB/T 8305‑2013, TS by phenol–sulfuric acid method, pH by calibrated glass electrode, conductivity using a portable meter, and antioxidant activity via spectrophotometric DPPH assay. Statistical tests include one-way ANOVA with Tukey’s multiple comparison (α = 0.05).

| **Power** | **TP_mean** | **TP_sd** | **CP_mean** | **CP_sd** | **WE_mean** | **WE_sd** | **TS_mean** |
| --- | --- | --- | --- | --- | --- | --- | --- |
| 0.3 | 20.6733686761858 | 1.58658281915294 | 7.3435016148705 | 0.668417317434132 | 42.202540029889 | 3.4283849124975 | 24.8126620435233 |
| 0.4 | 21.5403431042104 | 1.49644724037766 | 7.71094953064117 | 0.70488071448398 | 45.5750142598509 | 3.86379164286669 | 26.4940838799287 |
| 0.6 | 22.7428580584697 | 1.59017455589197 | 8.37677056474996 | 0.709892050629149 | 50.70893456 | 4.11241540595961 | 29.7751765374722 |
| 0.8 | 24.4044702630002 | 1.57815246232529 | 9.04233603081809 | 0.707148943470977 | 56.3777616546215 | 3.69058938609282 | 33.3092218233077 |

Continuation of Table 3.2

| **TS_sd** | **pH_mean** | **pH_sd** | **Conductivity_mean** | **Conductivity_sd** | **AO_mean** | **AO_sd** |
| --- | --- | --- | --- | --- | --- | --- |
| 1.70438192180394 | 5.00455414950899 | 0.128903519693696 | 1.60269521857814 | 0.181988344417345 | 73.9234864176751 | 4.54813617066547 |
| 1.68756070049243 | 4.99184421757314 | 0.123122077289159 | 1.65330957362767 | 0.172187642318816 | 74.722989806375 | 4.6573871774228 |
| 1.65322304884374 | 4.97242622746188 | 0.126752999119351 | 1.75778246625293 | 0.181839217307103 | 76.7101562307379 | 4.36110485093928 |
| 1.70203524821308 | 4.95105404303017 | 0.124435824645039 | 1.86064835780058 | 0.181943051481721 | 78.7493027394377 | 4.35992842706741 |
