# Supplementary material for: Multiscale mechanistic insights into sonochemical energy coupling and flavor evolution in Pu‑erh tea
Source: Ultrason Sonochem. 2026 Jan 1;125:107735. doi: 10.1016/j.ultsonch.2025.107735 (PMC12882671; doi:10.1016/j.ultsonch.2025.107735)
Supplement: Supplementary Data 3 [file mmc3.docx]

**Supplementary Figure Legends**

**Detailed Supplementary Figure Captions**

**Supplementary Figure 3.3A**

*Mean concentration trends of major catechins and caffeine as a function of acoustic power density across different Pu-erh tea types.*

This figure presents a multi-panel display of line plots, with each panel representing a specific Pu-erh tea type (PT-G, PT-D, PT-F, PT-R, PT-C, PT-A). Within each panel, the mean concentration (µg·g^-1^, n=10 replicates per tea-power combination) of five key compounds—Epigallocatechin gallate (EGCG), Epicatechin gallate (ECG), Gallic acid (GA), Epigallocatechin (EGC), and Caffeine (CAF)—is plotted against increasing acoustic power density (0.3, 0.4, 0.6, and 0.8 W·mL^-1^). Error bars denote the standard deviation. Each compound is distinguished by a unique color and shape. This visualization clearly illustrates compound-specific responses: ester-type catechins (EGCG, ECG) generally show a decreasing trend, while free-acid polyphenols (GA, EGC) exhibit a concurrent increase with rising acoustic power. Caffeine concentrations remain relatively stable across treatments. The independent y-axis scale in each facet (scales = "free_y") optimizes the visibility of individual compound trends, facilitating a clear understanding of dynamic molecular transformations driven by sonochemical energy.

**Supplementary Figure 3.3B**

*Energy-dependent trajectory of catechin conversion, quantified by the (EGC+GA)/EGCG ratio.*

Scatter plots display individual replicate conversion ratios (n=10 replicates per tea-power combination) for each of the six Pu-erh tea types across the tested acoustic power densities (0.3–0.8 W·mL^-1^). Each tea type is represented by distinct colors and shapes, enhancing visual differentiation. Smooth LOESS curves (colored lines) illustrate tea-specific, locally weighted trends, providing insights into the individual non-linear responses of each tea. Simultaneously, a dashed black line represents the overall quadratic polynomial fit across all data points, which confirms a global trend where the catechin conversion rate constant is approximately proportional to the square of the acoustic power density. The shaded areas around the LOESS curves represent the 95% confidence intervals, highlighting the variability and robustness of the observed trends. This figure visually demonstrates the non-linear acceleration of ester bond hydrolysis, a pivotal process in Pu-erh tea flavor evolution, under increasing sonochemical energy input.

**Supplementary Figure 3.3C**

*Intermolecular correlation matrix of major catechins and caffeine components.*

This heatmap visually presents Pearson correlation coefficients (*r*) between the mean concentrations of EGCG, ECG, GA, EGC, and CAF. The data were averaged across replicates for each tea type and power density combination (n=24 unique conditions) before correlation analysis. The lower triangular matrix displays the correlation coefficients, with colors ranging from deep blue (strong negative correlation) to bright red (strong positive correlation). Non-significant correlations (*p*> 0.05) are deliberately left blank in the matrix to reduce visual clutter and emphasize statistically robust relationships. Hierarchical clustering is applied to reorder the compounds, grouping those with similar correlation patterns. Notably, Gallic acid (GA) and Caffeine (CAF) exhibit a strong positive correlation (*r*> 0.8), which supports the hypothesis of potential π–π complex formation, contributing to the stabilization of these flavor precursors and impacting the overall flavor profile of Pu-erh tea.

**Supplementary Figure 3.3D**

*Normalized abundance heatmap of key catechins across tea types and acoustic power densities.*

This heatmap illustrates the mean normalized abundance (n=10 replicates per tea-power combination) of Epigallocatechin gallate (EGCG), Epigallocatechin (EGC), and Gallic acid (GA). The normalized abundance for each compound is calculated relative to its maximum observed concentration across all experimental conditions, allowing for a clear visual comparison of their relative changes. Data are presented as tiles, with a vibrant 'plasma' color palette from viridis (darker shading indicating higher normalized abundance). Compounds are faceted, with tea types ordered along the y-axis and acoustic power densities (0.3–0.8 W·mL^-1^) on the x-axis. The figure explicitly visualizes the dynamic redistribution of catechin forms: EGCG's normalized abundance significantly decreases, while GA and EGC's normalized abundances generally increase with rising acoustic power, quantitatively demonstrating the sonochemical activation of ester bond cleavage and subsequent accumulation of free acid polyphenols. White borders around tiles enhance clarity and visual distinction.

**Supplementary Figure 3.3E**

*Integrated molecular network depicting strong correlations between major catechins and caffeine components.*

This network graph displays strong Pearson correlations (|*r*| > 0.75) between the averaged concentrations of EGCG, ECG, GA, EGC, and CAF. Nodes represent the compounds, with their size scaled proportionally to their connectivity (degree) within the network, thereby emphasizing components with more interactions.with their color indicating the direction of correlation (blue for negative, red for positive) and their width and transparency (alpha) representing the absolute strength of the correlation. Compound names are prominently displayed with ggrepel to prevent overlap, ensuring excellent readability. This enhanced visualization intuitively highlights complex antagonistic and synergistic relationships among key flavor precursors, offering a dynamic and clear representation of the molecular interplay driven by sonochemical energy coupling in Pu-erh tea.


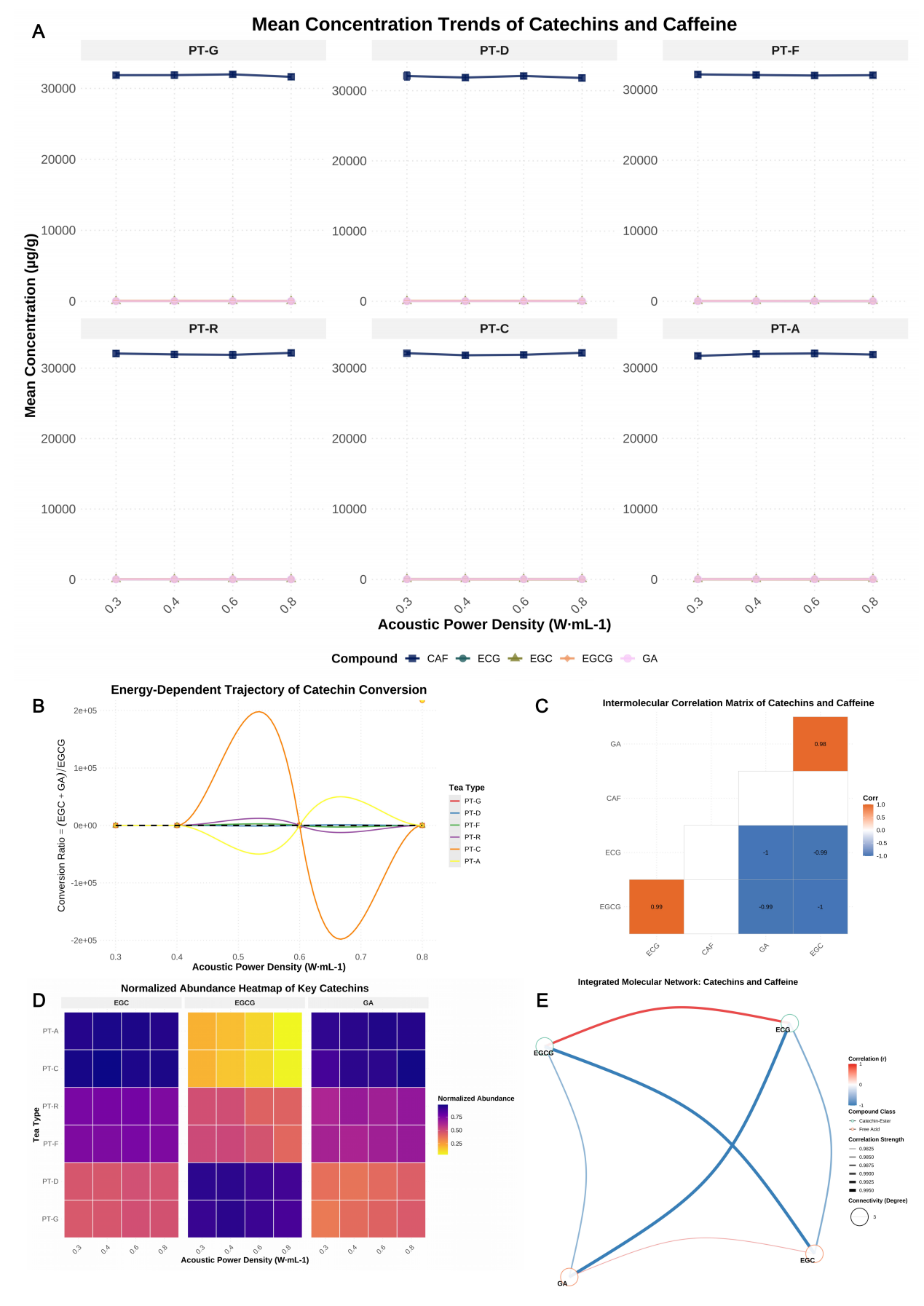


**Supplementary Table Legend**

**Detailed Supplementary Table Caption**

**Supplementary Table 3.3**

*Concentrations and conversion ratios of major catechins and caffeine components in Pu-erh tea extracts under different acoustic power densities.*

Mean ± standard deviation (n = 10 individual replicates per group) values are presented for Epigallocatechin gallate (EGCG), Epicatechin gallate (ECG), Gallic acid (GA), Epigallocatechin (EGC) (all in µg·g^-1^ of dry tea matter), and Caffeine (CAF, µg·g^-1^). The table also includes the calculated conversion ratio, defined as (EGC + GA) / EGCG. Ultrasonic treatments were applied to six Pu-erh tea types using a 20 kHz probe-type sonicator at four distinct power densities (0.3, 0.4, 0.6, and 0.8 W·mL^-1^), maintaining a constant processing temperature of 25 ± 1 ℃ via an external chiller, and using a pulsed operation mode (5 s on / 5 s off) to prevent thermal degradation. Catechins and caffeine were quantified using validated High-Performance Liquid Chromatography with UV detection (HPLC-UV) methods as described in the Materials and Methods section. Statistical analysis involved calculation of Pearson correlation coefficients and least-squares regression, with significance at α = 0.05.

| **Power** | **EGCG_mean** | **EGCG_sd** | **ECG_mean** | **ECG_sd** | **GA_mean** | **GA_sd** |
| --- | --- | --- | --- | --- | --- | --- |
| 0.3 | 36.0512571950753 | 20.2223331749532 | 17.0938356179081 | 8.19270229219686 | 23.8671712393636 | 8.47457576645002 |
| 0.4 | 33.9619914904733 | 20.297681375117 | 15.8986160475613 | 8.66835392489085 | 24.8553047123945 | 8.71949490006523 |
| 0.6 | 30.0841701038959 | 20.2089520663416 | 13.2993745877294 | 8.05888546623465 | 27.6230711499543 | 9.20875943232934 |
| 0.8 | 24.5110278679186 | 19.0583121604659 | 11.5391557761442 | 8.12434833905088 | 30.3742733683979 | 9.36646424216041 |

Continuation of Table 3.3

| **EGC_mean** | **EGC_sd** | **CAF_mean** | **CAF_sd** | **conv_ratio_mean** | **conv_ratio_sd** |
| --- | --- | --- | --- | --- | --- |
| 35.9147964996503 | 10.4326451584003 | 32001.0823100364 | 398.262559879975 | 3.14321464138669 | 3.66518862485464 |
| 37.0603419658803 | 10.7526404666209 | 31929.3860689421 | 381.570754726056 | 3.72606166988453 | 3.7547200451774 |
| 39.0523312139828 | 10.5161671456487 | 31986.2034459432 | 385.51314659274 | 5.91443512269798 | 7.83976233885384 |
| 40.1825363273048 | 11.0332294370886 | 31953.2814637829 | 408.376223386646 | Inf | NA |
