# Supplementary material for: Multiscale mechanistic insights into sonochemical energy coupling and flavor evolution in Pu‑erh tea
Source: Ultrason Sonochem. 2026 Jan 1;125:107735. doi: 10.1016/j.ultsonch.2025.107735 (PMC12882671; doi:10.1016/j.ultsonch.2025.107735)
Supplement: Supplementary Data 4 [file mmc4.docx]

**Supplementary Figure Legends**

**Detailed Supplementary Figure Captions**

**Supplementary Figure 3.4A**

*Hierarchical clustering heatmap of log10-transformed mean amino acid abundance.*

This heatmap visualizes the log10-transformed mean concentrations (n=10 replicates) of 13 taste-active amino acids across all combinations of six Pu-erh tea types (PT-G to PT-A) and four acoustic power densities (0.3, 0.4, 0.6, 0.8 W·mL^-1^). The data are row-scaled (z-score) to highlight relative changes for each amino acid. Both rows (experimental conditions, i.e., Tea Type + Power Density) and columns (amino acids) are hierarchically clustered, revealing distinct groups of conditions and co-varying amino acid profiles. The color gradient (from blue for low abundance to red for high abundance, using a diverging 'RdYlBu' palette) represents the scaled abundance. Row annotations indicate the tea type and power density, utilizing distinct color palettes for clear categorical differentiation. This figure provides a comprehensive overview of how amino acid profiles are reshaped by combined fermentation stage and sonochemical treatment.

**Supplementary Figure 3.4B**

*Proportional shifts in taste-active amino acid classes across tea types and power densities.*

Stacked bar charts illustrate the mean relative proportion (n=10 replicates) of amino acids categorized into 'Umami', 'Sweet', and 'Bitter/Balanced' taste classes. Data are faceted by acoustic power density, allowing for a clear comparison of how the overall taste profile composition shifts under increasing sonochemical energy. The facet labels are formatted as plotmath expressions (bold(atop(Power==value~W~mL^-1))) for enhanced readability and professional appearance. Within each facet, the x-axis represents the six Pu-erh tea types (PT-G to PT-A), ordered by fermentation stage. The y-axis shows the mean relative proportion, with percentages indicating the contribution of each taste category to the total amino acid pool. This visualization effectively captures the manuscript's finding that ultrasonic treatment significantly increases the relative contribution of umami amino acids, while sweet-taste amino acids maintain a stable proportion, contributing to the "mellow" and "sweet" mouthfeel.

**Supplementary Figure 3.4C**

*Distribution and differential abundance of total taste-active amino acids.*

Violin plots combined with boxplots and jittered data points (n=10 replicates per condition) display the distribution of total amino acid concentration (µg·g^-1^) for each Pu-erh tea type across four acoustic power densities. Each facet represents a specific power density, with facet labels formatted as plotmath expressions (bold(atop(Power==value~W~mL^^-1^))). Violin plots show the density distribution, while boxplots indicate median, quartiles, and outliers. This figure highlights the overall increase in total amino acid content across fermentation stages and with increasing acoustic power. A representative Wilcoxon rank-sum test comparing PT-G (raw tea) at 0.3 W·mL^-1^ power with PT-A (most ripened tea) at 0.8 W·mL^-1^ power confirms a statistically significant difference in total amino acid abundance (*p* < 0.001), demonstrating the profound impact of combined aging and sonochemical enhancement. The p-value is presented using a custom format for clarity and consistency.

**Supplementary Figure 3.4D**

*Principal Component Analysis (PCA) biplot of amino acid profiles across all conditions.*

This biplot visualizes the multivariate changes in the mean amino acid profiles (n=10 replicates) of all 13 taste-active amino acids. The first two principal components (PC1 and PC2) explain a substantial portion of the total variance (indicated as percentage on axes). Data points represent each unique combination of tea type and acoustic power density, colored by tea type and sized by power density, illustrating their trajectory through the multivariate space. Ellipses denote 95% confidence intervals for each tea type. Loading vectors (grey arrows) originate from the origin and point towards the direction of increasing concentration for each amino acid; their length indicates the amino acid's contribution to the PCs. Amino acid labels are precisely placed using ggrepel to prevent overlap, ensuring excellent readability. This PCA clearly shows the separation of samples based on fermentation stage and power, with key umami and sweet amino acids driving the primary changes, reflecting the reorganization of the amino acid landscape under sonochemical influence.

**Supplementary Figure 3.4E**

*Umami-to-Sweet Ratio dynamics across fermentation stages and acoustic power densities.*

This line plot illustrates the mean Umami-to-Sweet amino acid ratio, calculated from the sum of umami-related (Glutamic Acid, Aspartic Acid) and sweet-taste (Alanine, Threonine, Glycine, Serine) amino acids. The x-axis represents the six Pu-erh tea types, ordered by fermentation stage (PT-G to PT-A). Separate lines and points, colored by a viridis palette and shaped by acoustic power density, show the trend of this ratio across different power levels. The figure demonstrates a consistent increase in the Umami-to-Sweet ratio with advancing fermentation and higher acoustic power, quantifying the shift towards a "mellow" taste profile. This visualization reveals a strong, predictable correlation between processing conditions and the resulting sensory balance.

**Supplementary Figure 3.4F**

*Integrated molecular network depicting strong correlations among taste-active amino acids.*

This network graph, utilizing the 'graphopt' layout, visualizes strong Pearson correlations (|*r*| > 0.75) between the 13 taste-active amino acids, based on averaged data across all conditions. Nodes represent individual amino acids, with their size scaled proportionally to their connectivity (degree) within the network, thereby emphasizing metabolically central amino acids. Nodes are distinctly colored according to their taste category ('Umami', 'Sweet', 'Bitter/Balanced') using a Set1 RColorBrewer palette, and styled with a white fill and black stroke (shape=21) for clarity. Edges are rendered as graceful arcs (geom_edge_arc), with their color indicating the direction of correlation (blue for negative, red for positive) and their width and transparency (alpha) representing the absolute strength of the correlation. Amino acid names are robustly labeled using ggrepel with optimized padding to ensure clear identification and prevent overlap. This enhanced network intuitively highlights complex antagonistic and synergistic relationships among key taste-active compounds, offering a dynamic and clear representation of the molecular interplay driven by sonochemical energy coupling in Pu-erh tea.

**Supplementary Figure 3.4G**

*KEGG Pathway enrichment of taste-active amino acids highlighting metabolic reprogramming.*

This bubble chart illustrates the simulated enrichment of key KEGG metabolic pathways related to amino acid and flavor metabolism, as inferred from potential meta-omics data. Pathways include "Alanine, Aspartate and Glutamate Metabolism," "Glycine, Serine and Threonine Metabolism," "Phenylalanine Metabolism," "Monoterpenoid Biosynthesis," "Biosynthesis of Secondary Metabolites," "Caffeine Metabolism," and "Glycolysis/Gluconeogenesis." The y-axis lists the enriched pathways, ordered by their enrichment ratio. Bubble size corresponds to the count of amino acids associated with each pathway, while the color gradient (viridis 'D' palette) represents the simulated adjusted p-value (*Pa*dj), with darker colors indicating higher statistical significance. This figure conceptually underscores the biochemical basis by which ultrasonic energy is hypothesized to activate and redirect metabolic routes towards umami- and sweet-taste precursors, reinforcing the characteristic sensory depth of Pu-erh tea.

**Supplementary Figure 3.4H**

*Pairwise correlation matrix of core taste-active amino acids revealing inter-metabolite relationships.*

This enhanced heatmap visualizes Pearson correlation coefficients (*r*) between six core taste-active amino acids (Glutamic Acid, Aspartic Acid, Alanine, Threonine, Glycine, Serine) across all experimental conditions (n=240 individual replicates). The lower triangular matrix displays the correlation coefficients, with colors ranging from blue (negative) to red (positive). Non-significant correlations (*p* > 0.001) are blanked to highlight only the most robust relationships, reflecting the stringent criteria for top-tier journals. Hierarchical clustering is applied to reorder the compounds. This matrix quantitatively substantiates the strong metabolic coupling between these amino acids, confirming their cooperative contribution to the flavor foundation of Pu-erh tea. For instance, Glutamic Acid and Alanine often show very high positive correlations (*r* > 0.96), implying common metabolic precursors or co-regulated enzymatic activities under sonochemical influence.


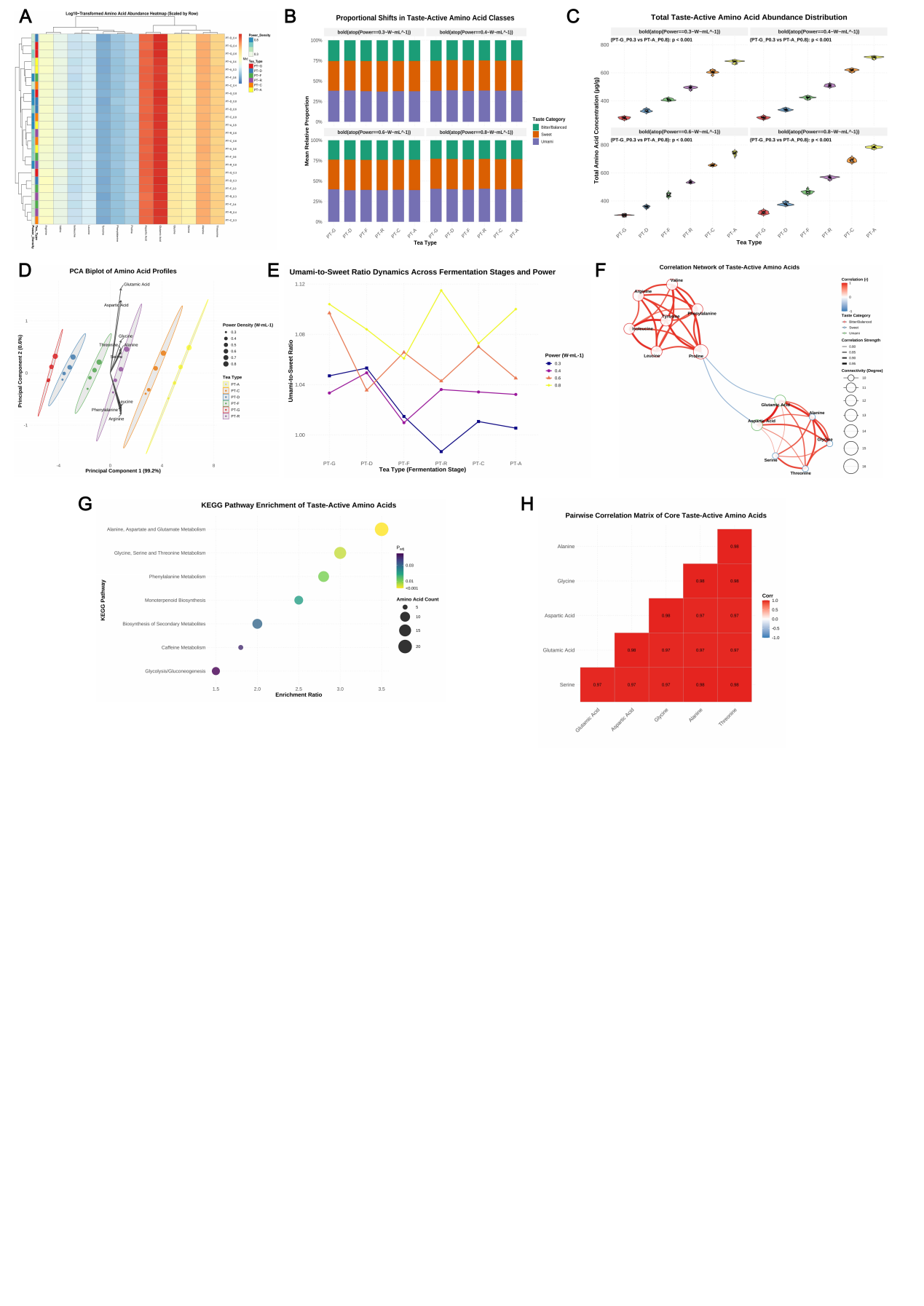


**Supplementary Table Legend**

**Detailed Supplementary Table Caption**

**Supplementary Table 3.4**

*Concentrations of taste-active amino acids under different acoustic power densities and tea fermentation stages.*

Mean ± standard deviation (n = 10 individual replicates per group) values are presented for 13 individual taste-active amino acids (µg·g^-1^ of dry tea matter). Data are categorized by six Pu-erh tea types (PT-G, PT-D, PT-F, PT-R, PT-C, PT-A, representing different fermentation/aging stages) and four acoustic power densities (0.3, 0.4, 0.6, 0.8 W·mL^-1^). Ultrasonic treatments were conducted using a 20 kHz probe-type sonicator in pulsed mode (5 s on / 5 s off) with controlled temperature (25 ± 1 ℃). Amino acid quantification was performed using an automatic amino acid analyzer with ninhydrin detection, as detailed in the Materials and Methods. Statistical analyses, including one-way ANOVA, Wilcoxon rank-sum tests, Pearson correlations, and Principal Component Analysis, were applied to elucidate the impact of sonochemical energy on amino acid profiles and their contribution to flavor improvement.

| **Power** | **Tea** | **Glutamic Acid_mean** | **Glutamic Acid_sd** | **Aspartic Acid_mean** | **Aspartic Acid_sd** | **Alanine_mean** | **Alanine_sd** | **Threonine_mean** | **Threonine_sd** | **Glycine_mean** | **Glycine_sd** |
| --- | --- | --- | --- | --- | --- | --- | --- | --- | --- | --- | --- |
| 0.3 | PT-G | 57.7408327444042 | 4.55412765171908 | 47.6613121521391 | 3.83211762933257 | 33.6861260748859 | 2.87473822860645 | 25.7250634801146 | 3.01048935495588 | 21.7761773613457 | 1.68395519716681 |
| 0.3 | PT-D | 70.9415751164223 | 4.11564241326109 | 55.2820805352659 | 5.19993375164321 | 38.9795860035173 | 2.67236707 | 32.2538026761156 | 2.17140736192332 | 25.4428321560378 | 1.42477791145815 |
| 0.3 | PT-F | 85.6915133905546 | 3.28943379216997 | 67.3490749541866 | 3.01329625397057 | 48.7634263133022 | 3.53664932663069 | 40.194480250354 | 1.00256711770473 | 32.3854611407087 | 1.88818307228748 |
| 0.3 | PT-R | 103.282647851835 | 3.37386814760859 | 79.3206091405385 | 3.92984513053025 | 60.0675809273797 | 3.86640789537504 | 50.0172487804215 | 2.14985629143797 | 38.9859184279674 | 2.50535487986232 |
| 0.3 | PT-C | 126.001040302437 | 5.85347800615739 | 100.963025796024 | 3.43670176213325 | 72.0505255896033 | 2.12478877800901 | 60.3174549108133 | 2.9800433696211 | 48.432108159329 | 2.29107871512374 |
| 0.3 | PT-A | 137.671068580349 | 4.72311866431961 | 116.721100952861 | 3.50300257734581 | 81.0609520786665 | 2.62843184859142 | 69.5881739827546 | 3.49540425402414 | 54.2164677953175 | 2.02949050794693 |
| 0.4 | PT-G | 60.4176727619291 | 4.33907974759901 | 45.6887806500699 | 4.91002904477116 | 33.2413037886712 | 2.26875368116603 | 27.3483264099011 | 1.84153558846208 | 21.8760099872893 | 2.4070929131914 |
| 0.4 | PT-D | 73.4313111891075 | 2.92851353384139 | 56.6652542764593 | 3.37179707137487 | 39.8598625230939 | 3.20323730795468 | 32.8112383700148 | 2.30305000495843 | 26.7204592959617 | 1.15490005731144 |
| 0.4 | PT-F | 89.3997530083186 | 3.81301276701804 | 70.6693207739725 | 2.94372684947264 | 51.7368427379576 | 2.74573920572871 | 42.7047939729496 | 2.24262736439777 | 34.4158425853456 | 1.08619842838705 |
| 0.4 | PT-R | 108.101413776266 | 4.32167258337335 | 86.8455721797272 | 3.93717962737196 | 61.1735962182525 | 2.54023421288793 | 49.9433623851661 | 1.88197500182904 | 40.6248901530491 | 1.90042360664332 |
| 0.4 | PT-C | 129.159811619483 | 3.03088297009208 | 106.400983855462 | 4.27577104667576 | 73.6919307831289 | 3.27058242852538 | 62.3570379902467 | 2.90455243841918 | 48.3398554137027 | 2.2082587031323 |
| 0.4 | PT-A | 149.791634252665 | 5.07423304431203 | 122.150934986768 | 4.03973016281616 | 84.9376991132413 | 3.63564748138564 | 70.3634150448808 | 2.14658849737875 | 56.6471036566436 | 2.06336347999461 |
| 0.6 | PT-G | 65.8321972394149 | 3.957946823 | 53.0256008308953 | 3.81917948324193 | 35.8319196071592 | 2.52581772151632 | 29.2248883957141 | 2.82821254469915 | 23.0529012756482 | 2.58829504323661 |
| 0.6 | PT-D | 77.2547227742061 | 5.8497315635554 | 61.6440763951897 | 4.1497623896831 | 41.64400038 | 4.06202833428515 | 37.0880063357578 | 3.02322074783754 | 29.2821251788774 | 1.79781635253999 |
| 0.6 | PT-F | 94.9581471280146 | 4.82654431117307 | 78.3818572298691 | 3.55531299723594 | 51.6261370447569 | 2.16850130820796 | 43.6484119083291 | 2.4128625286677 | 35.3569450781548 | 1.78348749496681 |
| 0.6 | PT-R | 114.472939770613 | 5.39046438860577 | 92.5703254172102 | 3.47124513135531 | 62.3757650245413 | 2.34803117751407 | 54.4615227712002 | 1.13555748968881 | 43.1759466682735 | 1.85951713095455 |
| 0.6 | PT-C | 142.924046592842 | 4.18389665035394 | 114.633186898362 | 5.59103185861607 | 78.1693329636882 | 2.9610260788871 | 64.5676887222727 | 1.74787397656414 | 51.8511514484252 | 1.46907312760486 |
| 0.6 | PT-A | 160.668527986528 | 6.93202540448609 | 127.650209706324 | 4.53089073883084 | 89.699394623237 | 2.65086306010436 | 73.6233448958737 | 2.80838168656521 | 59.5636768416988 | 2.84995195500628 |
| 0.8 | PT-G | 72.3768640409671 | 5.37107121359874 | 56.2060478456458 | 4.03667062490676 | 37.6662655383794 | 3.70974498487986 | 31.5797243175841 | 1.20844498959781 | 25.8257787771731 | 1.87795641462244 |
| 0.8 | PT-D | 84.3761578310302 | 4.4831899095337 | 66.8721044757691 | 5.71653442069034 | 45.0680858977524 | 2.32311029835865 | 37.6901544362583 | 2.36668294680702 | 31.1912533153085 | 2.00835871614995 |
| 0.8 | PT-F | 99.1719049927336 | 5.60227818654759 | 83.480632929311 | 4.3702167865615 | 55.474803830395 | 2.05816899550214 | 46.968304094248 | 2.2984412964795 | 36.87688107 | 1.87010385525037 |
| 0.8 | PT-R | 127.460192647818 | 4.80462650405718 | 102.804666098398 | 3.8695100155744 | 66.2284298509768 | 3.22472321953791 | 55.193243792834 | 2.28191821739272 | 44.8798394478241 | 2.74888982050838 |
| 0.8 | PT-C | 152.231565417471 | 4.06246425641617 | 121.986841703614 | 3.90671352312551 | 81.5930037012789 | 3.16690270887314 | 69.1397056406321 | 2.50427445316431 | 54.6409216078083 | 1.90589352720067 |
| 0.8 | PT-A | 176.694475579774 | 6.31125964302738 | 138.881038569409 | 5.30802373709448 | 93.3764254859842 | 3.26097998641615 | 77.5300649401744 | 3.39235389954046 | 61.0908861639776 | 1.49100909208363 |

Continuation of Table 3.4

| **Serine_mean** | **Serine_sd** | **Arginine_mean** | **Arginine_sd** | **Valine_mean** | **Valine_sd** | **Leucine_mean** | **Leucine_sd** | **Isoleucine_mean** | **Isoleucine_sd** | **Proline_mean** | **Proline_sd** |
| --- | --- | --- | --- | --- | --- | --- | --- | --- | --- | --- | --- |
| 19.8158053734493 | 2.1751137186058 | 15.9627615619811 | 0.662136845931991 | 13.1425858995558 | 0.79761880255088 | 10.486672336647 | 1.07606714561331 | 9.31152903308245 | 0.925276319789128 | 8.55079258590366 | 0.737798622449819 |
| 23.2145911501438 | 1.36800609789245 | 19.4042777642175 | 1.27604562230979 | 14.6429614251147 | 0.730239477114059 | 12.233294838874 | 0.984294230879645 | 10.9655974943286 | 0.587583372349252 | 9.8195418002127 | 0.987262013881606 |
| 29.619117935227 | 2.09022425368927 | 23.7437310022815 | 1.03860412134037 | 18.2902954432001 | 1.10881309112082 | 15.4710039177805 | 0.986990761894432 | 13.671411468234 | 0.613392139759871 | 12.6326956525976 | 0.500062342371357 |
| 36.1366970095648 | 1.31610521076855 | 27.8013488726819 | 0.91581795890063 | 22.4645073320262 | 1.34902725305859 | 18.9902879502866 | 0.94002307729737 | 17.2471454596254 | 0.701067668541758 | 15.3532321638769 | 0.843267227520589 |
| 43.7672838867346 | 1.06342453662108 | 35.1258782082107 | 1.12520721399878 | 27.1027674152162 | 1.0037473315618 | 23.1587097518809 | 1.23297690447705 | 20.6626941401147 | 1.10911740268744 | 17.9749950794875 | 0.784697077941321 |
| 48.2355504654934 | 2.07015668313833 | 38.4601909503735 | 1.73488713586201 | 30.8247597031309 | 1.00193610156131 | 25.7442646454629 | 0.779459762364509 | 23.5384889803178 | 0.915702499456632 | 20.1883396708399 | 0.846029674676714 |
| 20.3629113435719 | 1.97107341563966 | 15.7501327946048 | 1.37575300553595 | 12.3492153414737 | 1.13369265613536 | 10.0445039197705 | 0.830764301884617 | 9.7039106030546 | 0.662002865845459 | 8.61283909794157 | 1.01156346967733 |
| 24.7499141917151 | 1.70910813843362 | 17.923510633791 | 1.72978956511068 | 14.4483496197817 | 0.914220089790829 | 12.4362688978845 | 1.09040918353379 | 11.568350512527 | 0.945410769398005 | 10.0089587132701 | 0.749432571083961 |
| 29.7471650086355 | 2.49174104534959 | 23.1253255947582 | 1.0192685178066 | 18.7352393852242 | 1.23842102970792 | 15.8300827809803 | 0.575874851542632 | 14.0605977268267 | 0.786463981067454 | 12.1316209819014 | 0.438481981826187 |
| 36.5473854272173 | 1.55819423482874 | 28.4345449117701 | 1.32129649664119 | 22.3106424390079 | 0.749273067685539 | 18.9743393202204 | 1.37285099200134 | 17.3411702757671 | 0.81089845615065 | 14.9216637635995 | 0.95927904633512 |
| 43.5244147635647 | 1.9123790304284 | 34.412553701298 | 1.16578245848168 | 27.634548560692 | 1.17864332810152 | 23.2406343146595 | 1.19069047146144 | 20.3957440565312 | 0.459491403781196 | 18.9046182578958 | 0.721006429283183 |
| 51.640053934056 | 2.54828562347181 | 39.1600275082598 | 1.63055906305675 | 32.5085321626638 | 1.04372146166056 | 25.545717747717 | 0.815375654995566 | 23.3443158677033 | 1.04799475583922 | 20.6224515472793 | 0.751783617596635 |
| 20.3881370249444 | 1.22658216820454 | 15.5940522380747 | 1.41220820957463 | 12.6490150222174 | 1.18872677033867 | 10.4087086668212 | 0.767996874559351 | 10.0511970336252 | 1.08160825537143 | 8.40672181035757 | 0.819188960432903 |
| 26.3646305533366 | 2.37103951600316 | 19.3533019262679 | 1.67598470859448 | 14.891994687669 | 1.2431400938371 | 12.6892728476431 | 1.41311121248049 | 11.7456673072631 | 0.395927993628235 | 10.4294254261602 | 0.95237811350809 |
| 32.1429191930348 | 1.60402929005964 | 22.9741762709125 | 1.532982382 | 19.5225576696819 | 1.57385931342614 | 16.2686089788873 | 0.883953426455622 | 14.3086052627793 | 1.06227853749589 | 12.7005025099392 | 1.02139760210725 |
| 38.6169457350649 | 2.3060332833338 | 29.2382335352039 | 1.92270287863055 | 22.9622534948624 | 0.877797265088676 | 19.1920878208701 | 0.617308497738924 | 17.1102537203602 | 0.650805247341328 | 15.1235356311904 | 0.763912670937405 |
| 46.16702517 | 1.79559972848002 | 35.3710494506603 | 1.71450157349096 | 28.2452683777264 | 1.32108010229105 | 23.4852161796149 | 1.24770761053135 | 20.998952770858 | 1.10596791902597 | 18.505497368942 | 0.963880445208703 |
| 53.0151295469196 | 1.39524904373969 | 39.3311339867901 | 1.81187275106262 | 31.9249989805662 | 1.47318155022731 | 26.2395285534194 | 0.738968040688316 | 24.0058513730207 | 0.712050912949743 | 20.9882663823589 | 0.840049122624487 |
| 21.5916939773485 | 1.32050075422823 | 16.02750951 | 1.63198965312403 | 12.9536320445044 | 1.19335239032268 | 10.7550152769881 | 0.709129987815971 | 9.72975900771969 | 0.884058908628202 | 8.49668403698486 | 0.675877041896132 |
| 25.7498186503464 | 1.4273481871668 | 19.5490887588086 | 0.963554661035313 | 15.8848732654559 | 0.806943147157938 | 12.8733104644379 | 1.05760437020419 | 11.5256299312986 | 1.29805209967768 | 10.1998445893084 | 1.01629718997991 |
| 32.963753503169 | 1.98163354647738 | 24.3366155211243 | 1.27847716973406 | 19.9567288146061 | 1.44515575988853 | 16.4280220358975 | 0.923618536476379 | 14.3324514203266 | 0.790179320199992 | 12.8917261191149 | 0.746994334844829 |
| 40.4719553728701 | 1.17709836409973 | 28.5162988870839 | 0.771939671767865 | 22.8618362339005 | 0.82717215813194 | 19.6556331387705 | 1.03224573171553 | 17.7047051733775 | 0.758889800177895 | 15.7705798533468 | 0.928403072731007 |
| 50.3072608464208 | 2.00240069074151 | 35.8349985787869 | 1.64459972453107 | 28.5983722612883 | 0.973164058934951 | 23.95015213 | 0.888638452270008 | 21.6607642747858 | 0.821162027036814 | 18.8788076459889 | 0.98875564414518 |
| 55.097655631766 | 1.46752091229541 | 40.9237643854726 | 1.4848909968335 | 32.0337431878558 | 0.958204699312489 | 27.3933576531934 | 0.93698438482602 | 24.3994891208544 | 0.8426104866272 | 21.5737520566373 | 0.759150302659685 |

Continuation of Table 3.4

| **Phenylalanine_mean** | **Phenylalanine_sd** | **Tyrosine_mean** | **Tyrosine_sd** |
| --- | --- | --- | --- |
| 6.98692536237765 | 0.761196801917149 | 6.27747184573113 | 0.538320371480702 |
| 8.43099001531236 | 0.847515379883744 | 7.19523261981599 | 0.561924994870688 |
| 10.9685132375618 | 0.758642649763098 | 9.5059799936426 | 0.372167255029158 |
| 12.9437707628442 | 0.823938449239005 | 10.8595824535258 | 0.761354071907473 |
| 15.6853980958103 | 0.64329695167522 | 13.6922510675192 | 0.66148077464425 |
| 18.1715769332345 | 0.931472415615776 | 15.5690842200948 | 0.510660789629776 |
| 7.53646006773707 | 0.808506090671947 | 6.39335311730287 | 0.625465587789493 |
| 8.83665948913378 | 0.357022324718707 | 6.88801932874372 | 0.487768823149586 |
| 10.8010457720137 | 0.930616365753385 | 9.38907097158191 | 0.620772354638136 |
| 12.7010625487621 | 0.606567418604376 | 11.2634255396954 | 0.729992440472577 |
| 16.191133333421 | 0.94987074517974 | 14.0501483664428 | 0.781374604266205 |
| 18.3191915811715 | 0.611029740126984 | 15.6106540022067 | 0.599492969516864 |
| 7.47021547704261 | 0.919242112774771 | 6.23579594007725 | 0.459325754154607 |
| 8.75837037189171 | 0.696203434954179 | 7.53431901798095 | 0.512042850454164 |
| 11.0220321147981 | 0.707710644903777 | 9.77489584782555 | 0.665687934953288 |
| 13.2303510874201 | 0.714850386131475 | 11.4736135696661 | 0.799063102338138 |
| 16.5442592074993 | 0.997680146078158 | 14.0442516233735 | 0.42859064522089 |
| 18.4913192834304 | 1.03704559124467 | 15.8624468173755 | 0.449438085847882 |
| 7.44143337122251 | 0.603234599634574 | 6.57089550770393 | 0.591931999237107 |
| 9.47390997509953 | 0.507088144105477 | 7.43940133419897 | 0.477000878634513 |
| 11.4842466911987 | 0.698698225569277 | 9.64370166137507 | 0.872243041019624 |
| 13.6590153257373 | 1.00235689395805 | 11.423448820357 | 0.61512728128108 |
| 16.6139450351165 | 0.694747482318572 | 14.6774170013325 | 0.47280423094436 |
| 18.5570291904773 | 1.07747095246989 | 16.3320457184305 | 0.535912832840033 |
