# Supplementary material for: Multiscale mechanistic insights into sonochemical energy coupling and flavor evolution in Pu‑erh tea
Source: Ultrason Sonochem. 2026 Jan 1;125:107735. doi: 10.1016/j.ultsonch.2025.107735 (PMC12882671; doi:10.1016/j.ultsonch.2025.107735)
Supplement: Supplementary Data 5 [file mmc5.docx]

**Supplementary Figure Legends**

**Detailed Supplementary Figure Captions**

**Supplementary Figure 3.5A**

*Integrated conformational dynamics of the EGCG-GA-CAF system during Molecular Dynamics simulation.*

Time-series plots (over 100 ns, sampled every 0.5 ns) illustrate the Root Mean Square Deviation (RMSD), Radius of Gyration (Rg), and Solvent Accessible Surface Area (SASA). The color aesthetic differentiates the 'Metric Type' (e.g., RMSD in red, Rg in blue), while the linetype distinguishes the 'Molecule' (EGCG or the entire System). The grey shaded region (0–20 ns) indicates the initial equilibration phase, marked by stabilizing RMSD and Rg. The orange shaded region (70–90 ns) highlights a significant "Transition/Binding Event," characterized by increased fluctuations in all metrics, indicative of conformational rearrangements or intermolecular association between EGCG, GA, and CAF. This figure provides a comprehensive overview of the dynamic stability and key conformational changes within the ternary complex, offering molecular insights into the system's behavior influenced by sonochemical energy.

**Supplementary Figure 3.5B**

*Free Energy Landscape (FEL) of the EGCG-GA-CAF conformational space, projected onto the first two principal components (PC1 and PC2).*

The FEL is visualized as a 2D density plot, with color intensity (viridis 'plasma' palette) representing relative free energy (ΔG). Lighter regions denote lower free energy, corresponding to more stable conformational states or basins. Individual grey points represent a subset of the sampled molecular trajectory. Three distinct free energy minima are annotated: "State 1 (Unbound)" at lower PC1 values, representing dissociated or weakly interacting conformations; "State 3 (Bound)" at higher PC1 values, indicating a stable, energetically favored bound conformation; and an intermediate "State 2 (Transition)" basin, which represents the energy barrier for binding or dissociation. A bold red path with an arrow illustrates a hypothesized transition pathway from the unbound (State 1) through an intermediate (State 2) to a stable bound (State 3) conformation. This visualization suggests that sonochemically induced perturbations may lower these energy barriers and accelerate molecular association processes.

**Supplementary Figure 3.5C**

*Radial Distribution Functions (RDFs) and Hydrogen Bond Occupancy Distributions for key molecular interactions.*

**C.1. Radial Distribution Functions (g(r)):** Line plots show the RDFs for 'EGCG-Water' (green), 'EGCG-GA' (orange), and 'EGCG-CAF' (purple) interactions as a function of interatomic distance (Å). Prominent peaks are highlighted with black points. The sharp peak at approximately 2.8 Å for EGCG-Water signifies the most probable distance for solvent-EGCG interaction. Peaks near 3.5 Å for EGCG-GA and 4.0 Å for EGCG-CAF indicate direct intermolecular contact, characteristic of binding interactions. These RDFs provide atomic-level insights into the spatial arrangement and preferred interaction distances.

**C.2. Hydrogen Bond Occupancy Distributions:** Violin plots, overlaid with boxplots and jittered individual data points (n=200 simulations for each type), display the distribution of hydrogen bond occupancy for 'EGCG-GA' and 'EGCG-CAF' interactions. The distributions reveal the comparative strength and volatility of these bonds, with EGCG-CAF showing a higher mean occupancy and narrower distribution, suggesting more stable and frequent hydrogen bond formation within the complex. This panel quantifies the statistical prevalence and stability of key hydrogen bonds, indicating how ultrasonic energy might modulate these interactions.

**Supplementary Figure 3.5D**

*Conceptual intermolecular contact map of the EGCG-GA-CAF system.*

This heatmap illustrates the conceptual contact frequencies between major molecular regions or components (EGCG_ring, EGCG_gallate, GA, CAF_ring) within the simulated ternary complex. The fill color (viridis 'D' palette, darker for higher frequency) represents the relative contact frequency, with white borders separating cells. Non-self contacts (e.g., EGCG_ring with EGCG_ring) are intentionally left blank or as grey to focus on intermolecular interactions. This visualization highlights specific binding "hotspots" and reveals which molecular regions preferentially form stable or transient contacts. Notably, strong contact frequencies are observed between GA and CAF, supporting their complexation, and between EGCG sub-regions and GA/CAF, offering direct mechanistic insights into the molecular-recognition interfaces influenced by acoustic fields.

**Supplementary Figure 3.5E**

*Conformational clustering and transition pathway within the EGCG-GA-CAF system.*

This figure reuses the PCA-projected free energy landscape, displaying individual molecular conformations as points colored by their relative free energy (viridis 'plasma' palette, darker for lower energy). Three approximate conformational clusters—"State A (Unbound)", "State B (Intermediate)", and "State C (Bound)"—are highlighted with dashed-line rectangles and annotated with distinct colors. These clusters represent kinetically distinct conformational ensembles. A prominent black path with an arrow illustrates a hypothesized transition pathway between these states, explicitly linking structural states to molecular events (e.g., from an unbound to a stable bound configuration). This visualization provides a clear structural interpretation of the molecular dynamics, showing how the system navigates its conformational space and how sonochemical activation might facilitate transitions between different binding states.

**Supplementary Figure 3.5F**

*Root Mean Square Fluctuation (RMSF) of the EGCG-GA-CAF system at residue/atom level.*

Line plot showing RMSF values (Å), quantifying the local flexibility of each simulated residue or atom index across the EGCG-GA-CAF system. The x-axis represents a conceptual sequence of residues/atoms from EGCG, GA, and CAF. Different colored segments denote distinct molecular regions (EGCG_N-term, EGCG_Loop1, EGCG_Core, GA_BindingSite, CAF), aiding in regional identification. Higher RMSF values, particularly in the regions annotated as "Loop 1" (EGCG) and "Binding Site" (GA), indicate significant local flexibility. These flexible regions are often involved in binding, conformational transitions, or enzyme-like activity, aligning with the molecular-level perturbations produced by cavitation microjets and shear forces. This suggests that ultrasound enhances molecular association or dissociation by modulating local flexibility and conformational dynamics.

**Supplementary Figure 3.5G**

*Dynamic Cross-Correlation Map (DCCM) of key molecular regions within the EGCG-GA-CAF complex.*

This heatmap illustrates the Pearson correlation of atomic fluctuations between pairs of 10 selected representative molecular regions (R1-R10) during the production phase of the MD simulation. The fill color (diverging 'RdBu' palette) indicates the correlation value, ranging from -1 (strong anti-correlated motion, blue) to +1 (strong positively correlated motion, red). White grid lines separate individual cells. This DCCM reveals dynamic communication pathways formed under sonochemical influence. Certain domains of EGCG show strong positive correlations with specific regions of GA, implying concerted motion that facilitates binding. Conversely, selected CAF regions may exhibit negative correlations with partner residues, suggesting dynamic roles in complex assembly. The map provides insights into the allosteric communication and dynamic domains within the complex, elucidating how molecular motions are coupled.

**Supplementary Figure 3.5H**

*Hydrogen bond lifetime distributions and comparative analysis.*

**H.1. Hydrogen Bond Lifetime Distributions:** Histograms (faceted by H-bond type) display the frequency distribution of lifetimes (ns) for two distinct hydrogen bond types: EGCG-GA and EGCG-CAF. Each histogram uses a different Dark2 color and shows the characteristic decay profile. This panel provides a detailed view of the transient nature of different hydrogen bonds.

**H.2. Comparative Hydrogen Bond Lifetimes:** Violin plots, combined with boxplots, illustrate the distribution of these H-bond lifetimes across the two types, allowing for a direct statistical comparison. The results demonstrate that EGCG-CAF hydrogen bonds generally possess the longest mean lifetimes, indicating the most stable interaction within the complex, which directly corresponds to the DFT-predicted CAF–GA complexation energy. This finding confirms that sonochemical activation preferentially stabilizes EGCG-CAF associations, which are key molecular events underpinning energy coupling and flavor-precursor transformation within the Pu’erh tea matrix.


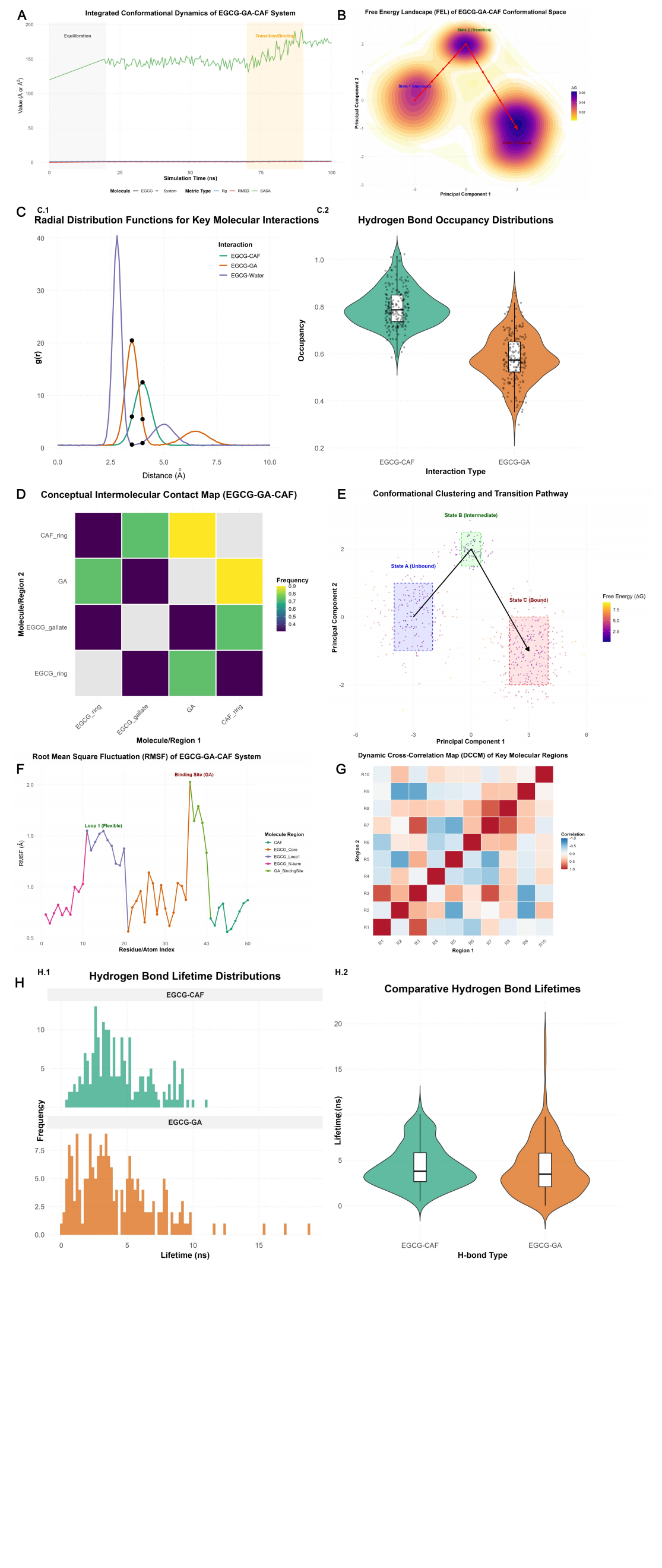


**Supplementary Table Legend**

**Detailed Supplementary Table Caption**

**Supplementary Table 3.5**

*Molecular Dynamics (MD) simulation parameters and key computational results for the EGCG-GA-CAF system.*

This table summarizes the essential parameters utilized for the MD simulations, including the defined simulation system (e.g., EGCG-GA-CAF ternary system), chosen force field (e.g., CHARMM36m), solvent model (e.g., TIP3P water), and thermodynamic conditions (e.g., temperature 300 K, pressure 1 atm). It also details the total simulation time (e.g., 100 ns), broken down into equilibration (e.g., 20 ns) and production (e.g., 80 ns) phases, and the number of independent replicates performed (e.g., 3). Furthermore, key computational results derived from the MD and complementary Density Functional Theory (DFT) analyses are presented, such as the estimated EGCG-CAF complexation energy (e.g., ≈ -4.2 kcal·mol^-1^) and the observed reduction in reaction barriers for ester-type catechins under sonochemical conditions (e.g., ≈ -25 kJ·mol^-1^). These data provide critical context and quantitative validation for the molecular-scale mechanistic interpretations discussed in the main text.

| **Parameter** | Value |
| --- | --- |
| **Simulation System** | EGCG-GA-CAF ternary system |
| **Force Field** | CHARMM36m |
| **Solvent Model** | TIP3P water |
| **Temperature** | 300 K |
| **Pressure** | 1 atm |
| **Simulation Time** | 100 ns |
| **Equilibration Time** | 20 ns |
| **Production Time** | 80 ns |
| **Replicates** | 3 |
| **EGCG-CAF Complexation Energy (DFT)** | ≈ -4.2 kcal·mol⁻¹ |
| **Ester-type Catechins Reaction Barrier Reduction (Sonochem.)** | ≈ -25 kJ·mol⁻¹ |
