# Supplementary material for: Multiscale mechanistic insights into sonochemical energy coupling and flavor evolution in Pu‑erh tea
Source: Ultrason Sonochem. 2026 Jan 1;125:107735. doi: 10.1016/j.ultsonch.2025.107735 (PMC12882671; doi:10.1016/j.ultsonch.2025.107735)

**Supplementary Figure Legends**

**Detailed Supplementary Figure Captions**

**Supplementary Figure 3.7A**

*Manhattan plots illustrating differential abundance of bacterial and archaeal taxa (16S rRNA gene sequencing) across Pu-erh tea types and ultrasonic power levels.*

Each panel displays the results of a specific pairwise comparison or contrast between experimental conditions, identified by the panel titles (e.g., "PT-A_P1_S1" likely denotes a comparison scenario for PT-A tea, Power level 1, and Sample/Comparison Set 1). The x-axis represents individual bacterial and archaeal taxa, organized into broader taxonomic groups (e.g., 's_bacterium', 's_archaea', 's_chloroplast'). The y-axis shows the negative base-10 logarithm of the adjusted *p*-value (-log10(Adjusted *P*-value)), where higher data points indicate stronger statistical significance. The horizontal dashed red line marks the significance threshold (e.g., adjusted *p*-value < 0.05 or False Discovery Rate (FDR) < 0.05). Points are colored and shaped according to the specific experimental conditions being compared, as detailed in the legend (e.g., "PT-A_0.3 vs 0.4", representing a comparison between 0.3 and 0.4 W·mL^-1^ ultrasonic power for PT-A tea). These plots supplement the overall microbial community structure analysis by pinpointing specific prokaryotic taxa that exhibit significant shifts in abundance. A diverse range of bacterial and archaeal taxa, including common 's_bacterium' and 's_archaea' groups, show dynamic responses to varying ultrasonic power and tea type, indicating a broad and active restructuring of the prokaryotic community. The presence of 's_chloroplast' sequences indicates carryover from the tea plant material itself.


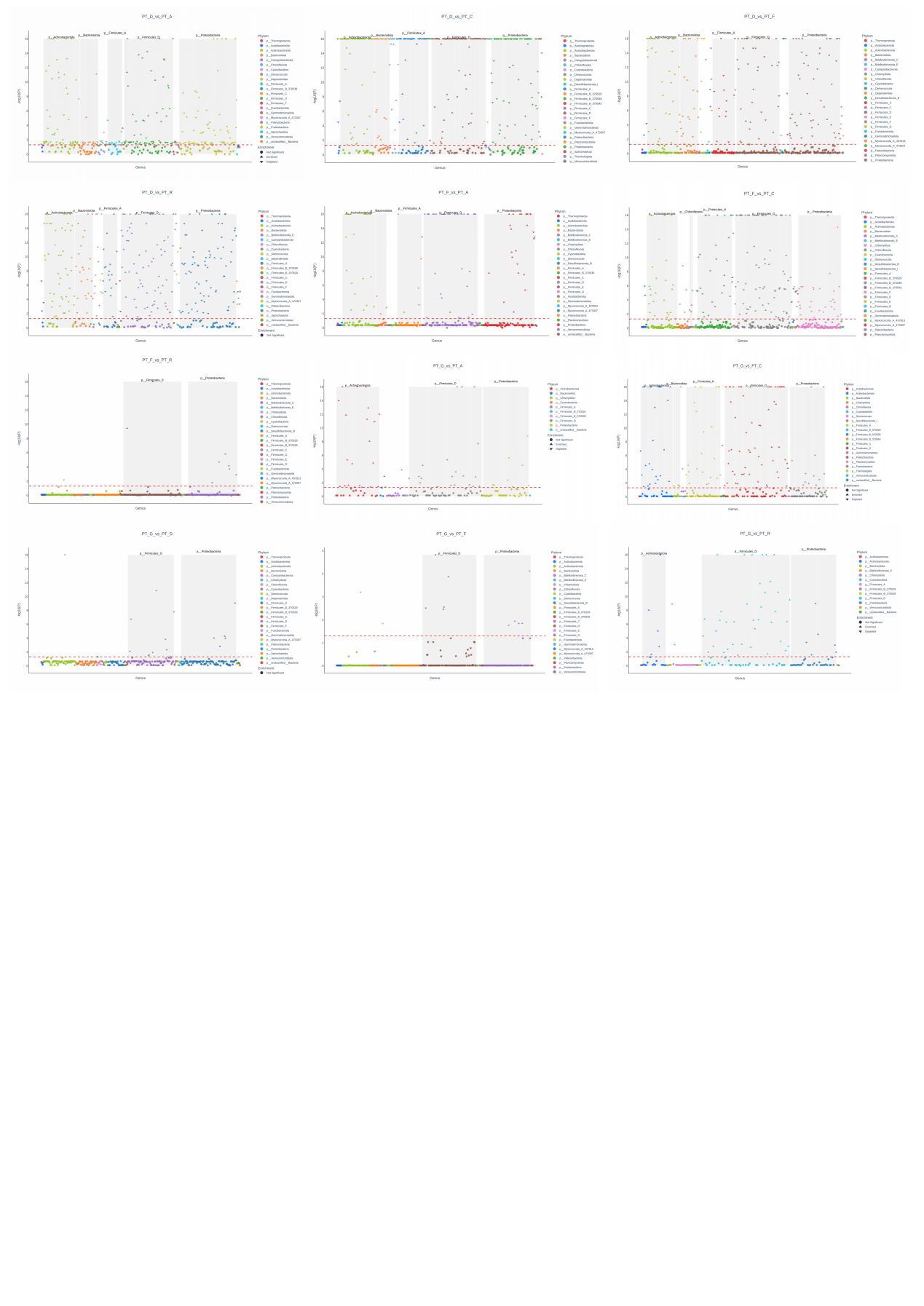


**Supplementary Figure 3.7B**

*Manhattan plots illustrating differential abundance of fungal taxa (ITS region sequencing) across Pu-erh tea types and ultrasonic power levels.*

Each panel displays the results of a specific pairwise comparison or contrast between experimental conditions, consistent with the format in Supplementary Figure 3.7A. The x-axis represents individual fungal taxa, organized into broader taxonomic groups (e.g., 's_fungi', 's_yeast'). The y-axis shows the negative base-10 logarithm of the adjusted *p*-value (-log10(Adjusted *P*-value)), where higher data points indicate stronger statistical significance. The horizontal dashed red line marks the significance threshold (e.g., adjusted *p*-value < 0.05 or False Discovery Rate (FDR) < 0.05). Points are colored and shaped according to the specific experimental conditions being compared, as detailed in the legend. These plots offer a parallel view of the fungal community's response to ultrasonic treatment. Compared to the 16S rRNA gene sequencing data (Supplementary Figure 3.7A), ITS sequencing generally reveals a lower diversity and fewer significantly differentially abundant fungal taxa. While some shifts are evident in 's_fungi' and 's_yeast' groups, the overall impact of ultrasonic power and tea type on the fungal community appears less pronounced than on the bacterial/archaeal communities. This suggests potential differences in the resilience, abundance, or metabolic roles of fungal populations to the applied sonochemical energy and fermentation stages.


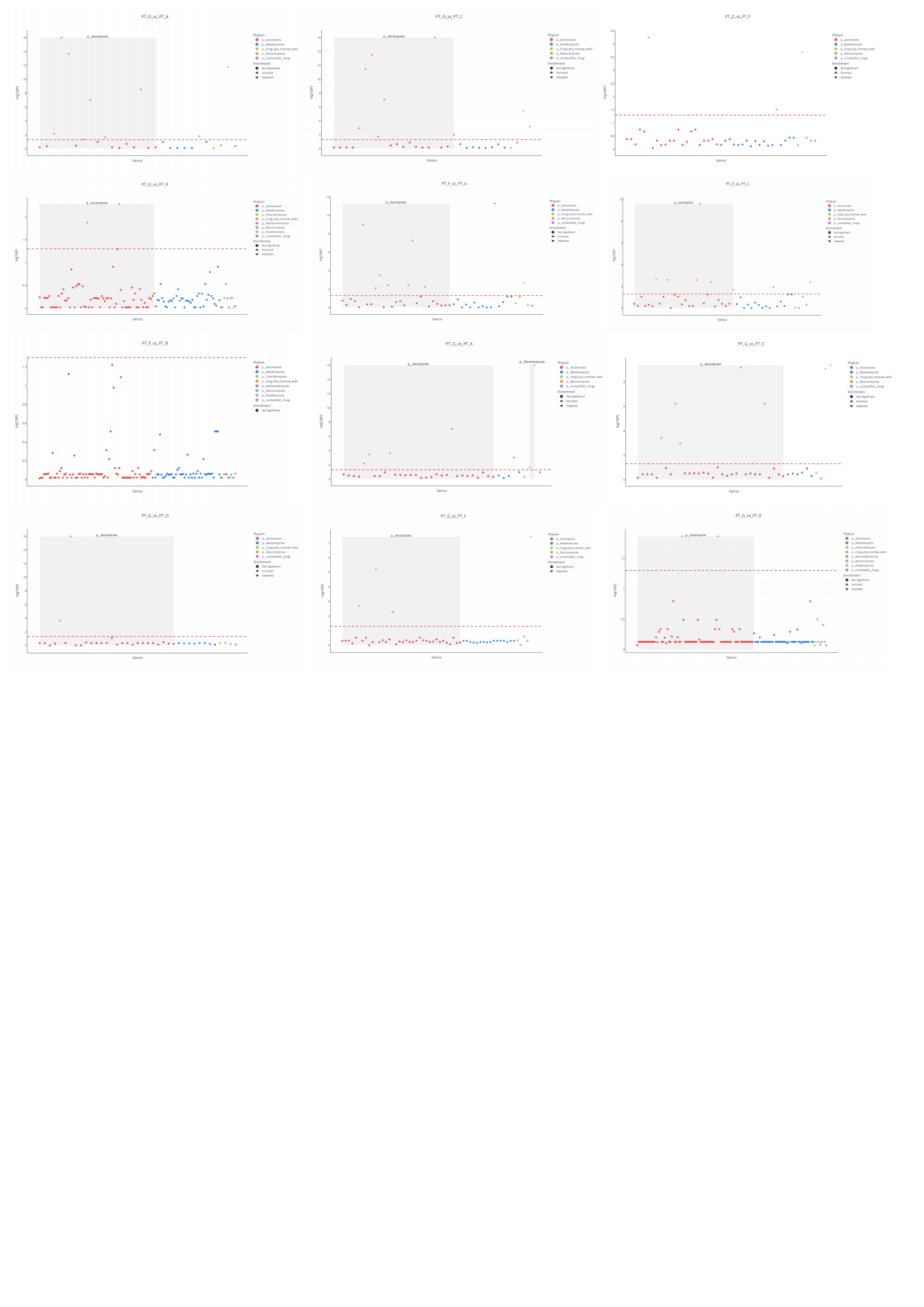

Supplement: Supplementary Data 7 [file mmc7.docx]
