# Supplementary material for: Multiscale mechanistic insights into sonochemical energy coupling and flavor evolution in Pu‑erh tea
Source: Ultrason Sonochem. 2026 Jan 1;125:107735. doi: 10.1016/j.ultsonch.2025.107735 (PMC12882671; doi:10.1016/j.ultsonch.2025.107735)
Supplement: Supplementary Data 8 [file mmc8.docx]

**Supplementary Figure Legends**

**Detailed Supplementary Figure Captions**

**Supplementary Figure 3.8A**

*Alpha diversity metrics of microbial communities across different acoustic power densities and tea fermentation stages.*

Boxplots (n=10 biological replicates per condition) illustrate the distribution of four alpha diversity indices: Shannon (diversity), Simpson (diversity), Chao1 (richness), and Observed ASVs (number of unique Amplicon Sequence Variants). Data are faceted by metric type (rows) and acoustic power density (columns, with labels formatted as bold(Power==value~W~mL^-1) for clarity and scientific notation), allowing for a comprehensive comparison. The x-axis represents the six Pu-erh tea types (PT-G to PT-A), colored by fermentation stage. This figure highlights significant variations in microbial richness and diversity in response to both fermentation time and sonochemical treatment. For example, lower diversity might be observed in initial tea types (PT-G), potentially increasing with ultrasonic power, while highly fermented teas (PT-A) might exhibit a different response pattern, indicating shifts in microbial ecosystem stability and functional capacity.

**Supplementary Figure 3.8B**

*Microbial community beta diversity visualized through Principal Coordinates Analysis (PCoA) based on Bray-Curtis dissimilarity.*

This 2D PCoA plot displays the clustering of microbial communities (represented by individual points, n=10 replicates per condition) from all samples. Each point is colored by tea type and shaped by acoustic power density, clearly illustrating separation in the microbial community structure. The axes (PCo1 and PCo2) display the percentage of total variance explained by the first two principal coordinates. Ellipses represent 95% confidence intervals around each tea type group. This figure demonstrates a clear separation of microbial communities primarily driven by tea fermentation stage (color), with secondary effects from acoustic power density (shape) indicating a systematic shift in community structure. This suggests that both intrinsic tea characteristics and external ultrasonic stimulation profoundly influence the overall microbial ecosystem composition.

**Supplementary Figure 3.8C**

*Relative abundance of key microbial genera across different acoustic power densities and tea fermentation stages.*

Stacked bar charts illustrate the mean relative abundance of seven dominant microbial genera (*Lactobacillus, Acetobacter, Bacillus, Pseudomonas, Streptococcus, Aspergillus, Candida*). Data are faceted by tea type (PT-G to PT-A), with each bar representing a specific acoustic power density (0.3–0.8 W·mL^-1^). Colors distinguish individual genera within the stack. This figure reveals dynamic changes in the microbial community structure, with some genera (e.g., *Lactobacillus, Acetobacter*) showing increased dominance under higher power and/or advanced fermentation stages, while others (e.g., *Pseudomonas*) might decrease in relative abundance. These shifts in relative abundance are crucial for understanding the microbial succession and their potential contribution to flavor development.

**Supplementary Figure 3.8D**

*Mean relative abundance trends of selected microbial genera across different acoustic power densities and tea fermentation stages.*

Line plots with error bars (representing standard deviation, n=10 replicates) depict the mean relative abundance of three selected key genera (*Lactobacillus, Acetobacter, Aspergillus*). Data are faceted by tea type (PT-G to PT-A), allowing for a detailed examination of how each genus's abundance changes with increasing acoustic power density. Different colors, linetypes, and shapes distinguish the genera. This figure clearly illustrates distinct response patterns: *Lactobacillus* and *Acetobacte*r, known for their roles in fermentation, generally increase with higher power, indicating their proliferation or enhanced activity. *Aspergillus*, a fungal genus, might show varied responses. These specific trends provide insights into the microbial taxa directly influenced by ultrasonic processing, which could drive specific metabolic pathways impacting flavor.

**Supplementary Figure 3.8E**

*Redundancy Analysis (RDA) biplot illustrating the influence of environmental factors and flavor metabolites on microbial community structure.*

This RDA biplot displays microbial communities (sites, as points colored by tea type and shaped by acoustic power density) constrained by acoustic power, tea type (treated as a numerical factor for RDA), and key flavor metabolites (biplot arrows). The x-axis (RDA1) and y-axis (RDA2) represent the constrained axes, with the percentage of explained variance indicated. Microbes (taxa, as green points) are also projected onto the space, showing their correlation with the environmental variables. The arrows represent the environmental (Power, Tea_Type) and metabolite variables, with their length indicating the strength of their correlation with the RDA axes, and their direction indicating the gradient. The plot shows how microbial community composition (clusters of points) is driven by specific metabolites (e.g., GA, Total_AA) and ultrasonic power, revealing critical ecological and biochemical links. The adjusted R-squared value, reported in the top-left, quantifies the explanatory power of the tested variables on microbial community variation.

**Supplementary Figure 3.8F**

*Spearman correlation heatmap between major microbial genera and key flavor metabolites.*

This heatmap visualizes Spearman correlation coefficients between the relative abundances of seven dominant microbial genera (y-axis) and seven key flavor metabolites (x-axis). The color gradient (diverging blue-red 'RdBu' palette) represents the correlation strength and direction, from -1 (strong negative correlation) to +1 (strong positive correlation). White grid lines separate cells, enhancing clarity. This figure systematically identifies potential synergistic or antagonistic relationships. For example, *Lactobacillus* might positively correlate with Umami amino acids (*Glutamic Acid, Alanine*), while EGCG (a decreasing catechin) might show negative correlations with genera thriving in fermented conditions. This provides direct evidence for the microbial involvement in shaping the flavor metabolite profile of Pu-erh tea.

**Supplementary Figure 3.8G**

*Co-occurrence network depicting strong correlations between microbial genera and flavor metabolites.*

This network graph (generated using a Fruchterman-Reingold layout) displays strong Spearman correlations (|*r*| > 0.6) between selected microbial genera and key flavor metabolites. Nodes represent either microbial genera or flavor metabolites, colored by their component type (green for microbial, orange for metabolite). Node size is scaled by its connectivity (degree), highlighting more influential components within the network. Edges are rendered as graceful arcs, with their color indicating the direction of correlation (blue for negative, red for positive) and their width and transparency (alpha) representing the absolute strength. This visualization reveals complex interdependencies, such as specific microbial genera forming strong positive correlations with increasing umami amino acids or decreasing ester-type catechins, directly linking microbial activity to the observed flavor transformations.


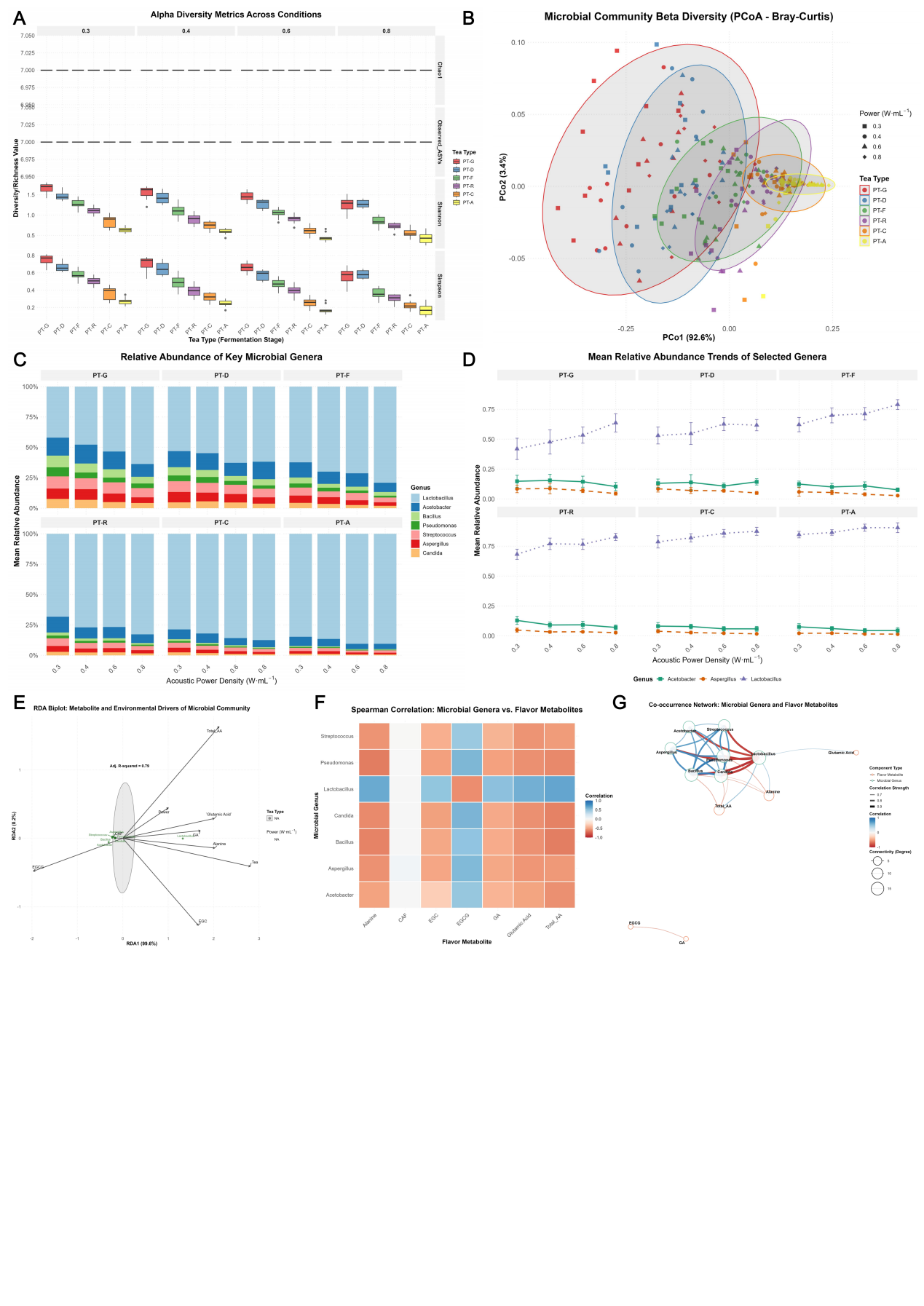


**Supplementary Table Legend**

**Detailed Supplementary Table Caption**

**Supplementary Table 3.8.1**

*Alpha diversity indices of microbial communities across different acoustic power densities and tea fermentation stages.*

Mean ± standard deviation (n = 10 individual biological replicates per group) values are presented for four key alpha diversity metrics: Shannon index, Simpson index, Chao1 index (a measure of species richness based on observed counts), and Observed ASVs (number of unique Amplicon Sequence Variants). Data are categorized by six Pu-erh tea types (PT-G, PT-D, PT-F, PT-R, PT-C, PT-A) and four acoustic power densities (0.3, 0.4, 0.6, 0.8 W·mL^-1^). These indices reflect the within-sample microbial richness and evenness, providing quantitative insights into how different processing conditions (fermentation and ultrasonication) influence the diversity of the tea's microbial ecosystem. Raw microbial read counts were used for the calculation of Chao1 and Observed ASVs, while Shannon and Simpson indices were computed from relative abundances.

| **Power** | **Tea** | **Shannon_mean** | **Shannon_sd** | **Simpson_mean** | **Simpson_sd** | **Chao1_mean** | **Chao1_sd** | **Observed_ASVs_mean** | **Observed_ASVs_sd** |
| --- | --- | --- | --- | --- | --- | --- | --- | --- | --- |
| 0.3 | PT-G | 1.66851401805507 | 0.121954740808713 | 0.751565857022762 | 0.0571718020885079 | 7 | 0 | 7 | 0 |
| 0.3 | PT-D | 1.47751531118762 | 0.119380010846408 | 0.66833845722175 | 0.0576676257828436 | 7 | 0 | 7 | 0 |
| 0.3 | PT-F | 1.27887098628792 | 0.123557867233226 | 0.57729835806212 | 0.0634708174648422 | 7 | 0 | 7 | 0 |
| 0.3 | PT-R | 1.11422806217071 | 0.0941919071729479 | 0.505850366196735 | 0.0501588600930349 | 7 | 0 | 7 | 0 |
| 0.3 | PT-C | 0.843012147727327 | 0.150655854887606 | 0.366399806325179 | 0.0768695942437731 | 7 | 0 | 7 | 0 |
| 0.3 | PT-A | 0.641186185983901 | 0.0703953186243041 | 0.273567074366531 | 0.0417461491592664 | 7 | 0 | 7 | 0 |
| 0.4 | PT-G | 1.55101617902878 | 0.163377544449183 | 0.704277927083745 | 0.0809579027853214 | 7 | 0 | 7 | 0 |
| 0.4 | PT-D | 1.43272007018004 | 0.151322373688803 | 0.647714255598003 | 0.0757369506346943 | 7 | 0 | 7 | 0 |
| 0.4 | PT-F | 1.10242987841257 | 0.15533405778091 | 0.486656539081368 | 0.0791729527363366 | 7 | 0 | 7 | 0 |
| 0.4 | PT-R | 0.896509697294051 | 0.127722615928809 | 0.391166123993538 | 0.066849507830965 | 7 | 0 | 7 | 0 |
| 0.4 | PT-C | 0.739375056014187 | 0.110902906656346 | 0.317079386741299 | 0.0539844189077266 | 7 | 0 | 7 | 0 |
| 0.4 | PT-A | 0.591311255901038 | 0.0744395125388214 | 0.24589049065998 | 0.0381838509124366 | 7 | 0 | 7 | 0 |
| 0.6 | PT-G | 1.45956051029874 | 0.113556093454667 | 0.663477771766104 | 0.0563439768629715 | 7 | 0 | 7 | 0 |
| 0.6 | PT-D | 1.2771932703263 | 0.116264224749271 | 0.574481249907691 | 0.0601999050842299 | 7 | 0 | 7 | 0 |
| 0.6 | PT-F | 1.04781185586844 | 0.124312430768669 | 0.467807101688107 | 0.0660044684287714 | 7 | 0 | 7 | 0 |
| 0.6 | PT-R | 0.906217582310369 | 0.111078699036718 | 0.396519546307969 | 0.06070271203942 | 7 | 0 | 7 | 0 |
| 0.6 | PT-C | 0.618785809779665 | 0.110850368283373 | 0.256908895782442 | 0.0527593582028926 | 7 | 0 | 7 | 0 |
| 0.6 | PT-A | 0.448057008363764 | 0.10289355533575 | 0.176773316854325 | 0.0503206661550548 | 7 | 0 | 7 | 0 |
| 0.8 | PT-G | 1.2579298890312 | 0.175221930586629 | 0.561401400721367 | 0.0879029237254444 | 7 | 0 | 7 | 0 |
| 0.8 | PT-D | 1.27843581967783 | 0.0922924279350794 | 0.581571207619559 | 0.0480316529473158 | 7 | 0 | 7 | 0 |
| 0.8 | PT-F | 0.847551001529707 | 0.125551695929709 | 0.363242324600949 | 0.0616730289485983 | 7 | 0 | 7 | 0 |
| 0.8 | PT-R | 0.720692329265538 | 0.0925086966182319 | 0.305725389158571 | 0.0472664694058746 | 7 | 0 | 7 | 0 |
| 0.8 | PT-C | 0.55512471486493 | 0.107755742921283 | 0.22893066430037 | 0.0546845987472295 | 7 | 0 | 7 | 0 |
| 0.8 | PT-A | 0.441791793030825 | 0.147161052087626 | 0.176499130120048 | 0.0708000438289404 | 7 | 0 | 7 | 0 |

**Supplementary Table 3.8.2**

*Spearman correlation matrix between major microbial genera and key flavor metabolites.*

This table presents the pairwise Spearman correlation coefficients between the relative abundances of seven dominant microbial genera (*Lactobacillus, Acetobacter, Bacillus, Pseudomonas, Streptococcus, Aspergillus, Candida*) and seven key flavor metabolites (*EGCG, GA, EGC, CAF, Glutamic Acid, Alanine, Total_AA*). The correlations were calculated using data from all samples (n=240). High absolute correlation values (e.g., > 0.6) indicate strong relationships, which can be positive (synergistic) or negative (antagonistic). This matrix quantifies the statistical associations, providing a critical basis for inferring mechanistic links between specific microbial taxa and the accumulation or transformation of flavor-active compounds during Pu-erh tea processing.

| **Microbial Genus** | EGCG | GA | EGC | CAF | Glutamic Acid | Alanine | Total_AA |
| --- | --- | --- | --- | --- | --- | --- | --- |
| **Lactobacillus** | -0.584838839 | 0.522051711412474 | 0.498267439162896 | -0.006510097 | 0.60925202895278 | 0.634132240605807 | 0.660249887205064 |
| **Acetobacter** | 0.482683472551599 | -0.429761432 | -0.307857141 | -0.005578234 | -0.505242507 | -0.508380589 | -0.576864565 |
| **Bacillus** | 0.503368125727876 | -0.470383064 | -0.486715869 | 0.00786474718228038 | -0.575619197 | -0.625009983 | -0.643764714 |
| **Pseudomonas** | 0.569573742465622 | -0.513865442 | -0.517069074 | 0.00242713863926253 | -0.577836084 | -0.646937484 | -0.604476882 |
| **Streptococcus** | 0.466833307799666 | -0.430974503 | -0.451764858 | -0.003761361 | -0.562168152 | -0.563912979 | -0.534455781 |
| **Aspergillus** | 0.558942666430018 | -0.488080778 | -0.488936267 | 0.0461212966541646 | -0.555649621 | -0.56996202 | -0.542562946 |
| **Candida** | 0.580234002325612 | -0.479352538 | -0.516001313 | 0.00943075407626761 | -0.578046022 | -0.601240521 | -0.629208992 |
