# Supplementary material for: Multiscale mechanistic insights into sonochemical energy coupling and flavor evolution in Pu‑erh tea
Source: Ultrason Sonochem. 2026 Jan 1;125:107735. doi: 10.1016/j.ultsonch.2025.107735 (PMC12882671; doi:10.1016/j.ultsonch.2025.107735)
Supplement: Supplementary Data 9 [file mmc9.docx]

**Supplementary Figure Legends**

**Detailed Supplementary Figure Captions**

**Supplementary Figure 3.9A**

*Sonochemical Parameters vs. Key Physicochemical Traits: Total Catechins and Total Free Amino Acids.*

This figure displays scatter plots illustrating the relationship between acoustic power density (x-axis) and the mean abundance of key physicochemical traits (y-axis). The plots are faceted by the specific trait being analyzed ('Total_Catechins' and 'Total_Amino_Acids'). Each point represents a unique sample condition (Tea Type x Power), with tea types distinguished by different shapes. Linear regression lines (solid colored lines) are fitted for each trait, illustrating the general trends. 'Total Catechins' generally show a decreasing trend with increasing power, influenced by tea type. Conversely, 'Total Amino Acids' exhibit an increasing trend with acoustic power. These plots provide a quantitative overview of how sonochemical treatment influences critical quality-defining compounds, highlighting their distinct responses.

**Supplementary Figure 3.9B**

*Sonochemical Parameters vs. Key Sensory Evaluation Scores.*

These panels present line and point plots examining the relationship between acoustic power density (x-axis) and five key sensory attributes (y-axis: Umami-Sweet Ratio, Mellow Smoothness, Astringency, Aftertaste Sweetness, Overall Acceptance). The data are faceted by Pu-erh tea type (PT-G to PT-A). Each line represents the trend of a specific sensory trait across increasing power densities for a given tea type. 'Umami-Sweet Ratio' and 'Aftertaste Sweetness' generally show an increasing trend with higher power, indicating improved taste profiles. 'Mellow Smoothness' tends to increase with later fermentation stages, while 'Astringency' generally decreases with increasing power. 'Overall Acceptance' scores, integrated from various factors, reflect the holistic sensory impact of sonication. These plots highlight the direct and nuanced impact of sonochemistry on sensory perception across different tea types.

**Supplementary Figure 3.9C**

*Correlation between Key Physicochemical Traits and Overall Sensory Acceptance.*

Scatter plots demonstrate the relationship between two prominent physicochemical traits ('Total Amino Acids' and 'Umami-Sweet Ratio') and the 'Overall Sensory Acceptance' score (on a 1-5 scale). Each point represents a single experimental sample. Data are faceted by the predicting trait, allowing for a focused analysis. Linear regression lines (solid colored lines) with 95% confidence intervals are fitted to quantify these relationships. A clear positive correlation is observed between higher total amino acid content and increased overall acceptance, as well as a positive correlation between the umami-to-sweet ratio and overall acceptance. These plots robustly quantify the direct link between specific chemical compositions and consumer preference, emphasizing how sonochemistry can optimize tea flavor by modulating these traits.

**Supplementary Figure 3.9D**

*Principal Component Analysis (PCA) biplot integrating sonochemical parameters, physicochemical traits, and sensory evaluation scores.*

This PCA biplot visualizes the multivariate relationships between experimental conditions (represented by sample points), and their corresponding sonochemical parameters, physicochemical traits, and sensory evaluation scores (represented by loading vectors or arrows). Sample points are colored by tea type and shaped by acoustic power density, revealing distinct groupings. The first two principal components (PC1 and PC2) explain the majority of the total variance, as indicated on the axes. Loading vectors (grey arrows) indicate the direction and strength of each variable's influence on the sample distribution. This integrated analysis reveals that samples treated with higher acoustic power and from later fermentation stages tend to cluster together, correlating strongly with increased amino acid content, higher umami-sweet ratios, and improved overall acceptance, as depicted by the alignment of these variable vectors. This figure comprehensively links processing conditions to chemical changes and sensory outcomes.


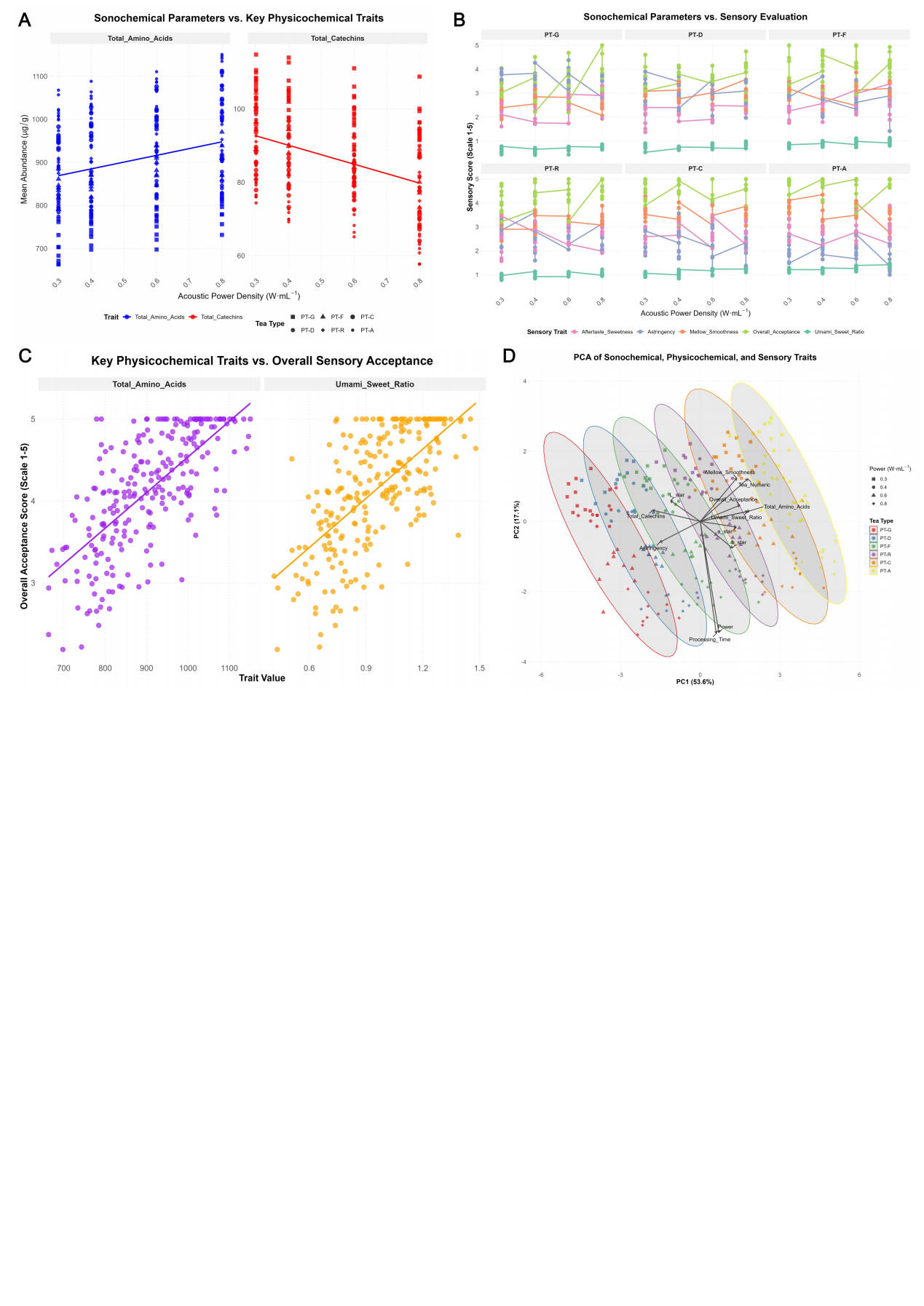


**Supplementary Table Legend**

**Detailed Supplementary Table Caption**

**Supplementary Table 3.9.1**

*Summary of physicochemical traits and sensory evaluation scores under different acoustic power densities and tea fermentation stages.*

Mean ± standard deviation (n = 10 individual replicates per group) values are presented for selected key sonochemical parameters, physicochemical traits, and sensory evaluation scores. Sonochemical parameters include Processing Time (minutes), Amplitude (%), and Frequency (kHz). Physicochemical traits encompass Total Catechins (µg/g), Total Free Amino Acids (µg/g), and CIELAB color values (L* a* b*). Sensory scores, provided on a hedonic scale of 1 to 5, include Umami-Sweet Ratio, Mellow Smoothness, Astringency, Aftertaste Sweetness, and Overall Acceptance. Data are systematically organized by acoustic power density (0.3, 0.4, 0.6, 0.8 W·mL^-1^) and Pu-erh tea type (PT-G to PT-A). This comprehensive table illustrates the quantitative impact of various processing conditions on both objective chemical attributes and subjective sensory perceptions of tea quality.

| **Power** | **Tea** | **Processing_Time_mean** | **Processing_Time_sd** | **Amplitude_mean** | **Amplitude_sd** | **Frequency_mean** | **Frequency_sd** | **Total_Catechins_mean** | **Total_Catechins_sd** | **Total_Amino_Acids_mean** | **Total_Amino_Acids_sd** |
| --- | --- | --- | --- | --- | --- | --- | --- | --- | --- | --- | --- |
| 0.3 | PT-G | 10.0180932124556 | 1.3121559178526 | 100 | 0 | 20 | 0 | 106.763160919858 | 4.51566920395058 | 714.334364667476 | 46.8965696061172 |
| 0.3 | PT-D | 10.3726170518034 | 0.922251744340926 | 100 | 0 | 20 | 0 | 101.686337599704 | 4.46031929206356 | 804.741251234602 | 19.4843449055344 |
| 0.3 | PT-F | 10.2856624052144 | 0.953914744312758 | 100 | 0 | 20 | 0 | 96.6697016538972 | 5.01365185970796 | 828.772179170823 | 31.9028721940596 |
| 0.3 | PT-R | 9.70365486323599 | 0.4776842998027 | 100 | 0 | 20 | 0 | 89.1617193725054 | 2.88085794018915 | 892.281804393814 | 10.5278919569087 |
| 0.3 | PT-C | 10.1650794051796 | 1.06128996446722 | 100 | 0 | 20 | 0 | 84.8229269801931 | 4.66536874007123 | 951.741607698359 | 15.1193752978498 |
| 0.3 | PT-A | 9.62239806864303 | 1.04950160689501 | 100 | 0 | 20 | 0 | 78.3652909890868 | 3.06795831888191 | 1017.15035084277 | 27.6151725339089 |
| 0.4 | PT-G | 11.5977545868501 | 0.87269014795991 | 100 | 0 | 20 | 0 | 105.395283568533 | 5.09108104924102 | 745.243355011409 | 29.0393046011541 |
| 0.4 | PT-D | 12.3851475619148 | 0.615400962334176 | 100 | 0 | 20 | 0 | 97.8956194022281 | 4.77673907488553 | 792.134819963351 | 17.1349062668225 |
| 0.4 | PT-F | 12.2160315094533 | 0.511123000323881 | 100 | 0 | 20 | 0 | 92.1586010388853 | 5.82537444880452 | 853.653070221153 | 18.1275400738062 |
| 0.4 | PT-R | 11.9753953241617 | 0.702949490104476 | 100 | 0 | 20 | 0 | 85.1480670099727 | 4.57726472236969 | 903.856372148999 | 27.4261285856108 |
| 0.4 | PT-C | 12.2684428119545 | 0.845957901676699 | 100 | 0 | 20 | 0 | 82.1297363596144 | 5.90247204720344 | 983.153217202867 | 24.158596139463 |
| 0.4 | PT-A | 11.8820371538745 | 0.73701743400918 | 100 | 0 | 20 | 0 | 74.5141612549325 | 5.05077709377049 | 1039.30642830908 | 25.0785217654271 |
| 0.6 | PT-G | 15.0581843224128 | 1.13218562195162 | 100 | 0 | 20 | 0 | 100.551265042026 | 5.82651084750913 | 761.815016921296 | 37.4208479066854 |
| 0.6 | PT-D | 14.6494990005067 | 0.936625658206667 | 100 | 0 | 20 | 0 | 93.1973801833533 | 4.79751146040507 | 816.152915386981 | 29.6971105753973 |
| 0.6 | PT-F | 15.8023279249795 | 1.10140895097615 | 100 | 0 | 20 | 0 | 84.0831727247046 | 4.35558218118227 | 900.082339950668 | 31.9960074971063 |
| 0.6 | PT-R | 15.3710810470212 | 0.993316306886781 | 100 | 0 | 20 | 0 | 83.0960066174303 | 2.82219121654595 | 943.730509064672 | 30.5999895057758 |
| 0.6 | PT-C | 14.952280015663 | 1.00021624787757 | 100 | 0 | 20 | 0 | 79.1208191678128 | 3.85311022459183 | 1014.4731104798 | 33.9803946242238 |
| 0.6 | PT-A | 14.4458933520445 | 0.767911665047552 | 100 | 0 | 20 | 0 | 72.3779841125438 | 4.81908366200672 | 1063.63480878875 | 34.5558500802579 |
| 0.8 | PT-G | 18.1911366319814 | 1.03532392453637 | 100 | 0 | 20 | 0 | 96.3527600183256 | 5.24218178726476 | 792.456941234601 | 30.5490633856149 |
| 0.8 | PT-D | 18.1177472108028 | 0.960715979419895 | 100 | 0 | 20 | 0 | 88.6277751523658 | 5.4403386514536 | 856.619312800412 | 31.9882703555662 |
| 0.8 | PT-F | 17.6065173445979 | 1.40501062065128 | 100 | 0 | 20 | 0 | 82.5886589597045 | 7.63575749575823 | 911.787519346175 | 31.1364099330885 |
| 0.8 | PT-R | 17.7683321962971 | 1.07130505325016 | 100 | 0 | 20 | 0 | 76.1084907229593 | 7.71674682120202 | 971.341194013937 | 37.4893463953843 |
| 0.8 | PT-C | 17.877307696446 | 1.33994125016681 | 100 | 0 | 20 | 0 | 69.6147690188781 | 3.56114128403629 | 1052.05840496628 | 39.3230253315483 |
| 0.8 | PT-A | 17.4995524315121 | 1.15212284881377 | 100 | 0 | 20 | 0 | 64.3570293304027 | 4.58421182990541 | 1100.16093513901 | 45.9189527298162 |

Continuation of Table 3.9.1

| **L_star_mean** | **L_star_sd** | **a_star_mean** | **a_star_sd** | **b_star_mean** | **b_star_sd** | **Umami_Sweet_Ratio_mean** | **Umami_Sweet_Ratio_sd** | **Mellow_Smoothness_mean** | **Mellow_Smoothness_sd** | **Astringency_mean** | **Astringency_sd** |
| --- | --- | --- | --- | --- | --- | --- | --- | --- | --- | --- | --- |
| 37.7581868311503 | 1.09355733163324 | 5.52274160677983 | 0.362970089373388 | 15.8333645279168 | 0.775392815691555 | 0.590506506341027 | 0.0993232439060879 | 2.5790385737167 | 0.390311516595192 | 3.58437003569818 | 0.337902040374017 |
| 38.1695098897863 | 0.871839932247492 | 5.55794328008016 | 0.373794535032804 | 15.801430747656 | 0.915175633769402 | 0.732523419861528 | 0.123996328138083 | 2.9130796880277 | 0.147101509976549 | 3.32220989179302 | 0.456170698746518 |
| 37.2343479726691 | 0.818970119701117 | 5.79985399004627 | 0.519279077509053 | 16.5364132459389 | 0.738844220426273 | 0.817168833581683 | 0.0768382220026542 | 3.05037184215539 | 0.259738778521106 | 2.86544075284705 | 0.286075069158206 |
| 36.7710924273287 | 1.285295166 | 6.24436075340041 | 0.415263457838167 | 17.2287017592808 | 1.01889791617822 | 0.90332173699141 | 0.0841233999596808 | 3.13313659744243 | 0.327454335522291 | 2.73538032751362 | 0.398536920531928 |
| 36.1719650579368 | 1.30540016572931 | 6.38709561533873 | 0.455682810393188 | 17.541822722349 | 1.12013591157061 | 1.04162472305912 | 0.0766494240963779 | 3.63559455469655 | 0.282830740694906 | 2.43306605343674 | 0.43436820847483 |
| 36.0998668470396 | 1.7777837646538 | 6.64646930491178 | 0.418394465106285 | 17.5915422323504 | 1.35260808922968 | 1.18580134710508 | 0.0702089895841427 | 3.7790544742257 | 0.263939089881431 | 2.00012923170327 | 0.3892821393224 |
| 37.7974882941835 | 1.62998222566409 | 5.61911360989497 | 0.297682225758178 | 16.5185219915967 | 0.466201612831554 | 0.604664132294106 | 0.0909360556595571 | 2.87516667661677 | 0.229500086419607 | 3.5360346829235 | 0.545863026615645 |
| 37.7008388524874 | 1.27396501786214 | 5.81112813025335 | 0.24442535825493 | 16.0039640337215 | 0.892008804514542 | 0.713838472592202 | 0.0515921830182504 | 2.91144983642737 | 0.305999657068493 | 3.14215534432465 | 0.566602731150551 |
| 36.8962454899404 | 1.66574989447713 | 5.78607093422277 | 0.673231932159265 | 16.459458567862 | 1.08673612145485 | 0.88722973216655 | 0.0895351354943329 | 2.92758351080496 | 0.292651708672373 | 2.72497860350945 | 0.544685412739021 |
| 37.0706792170299 | 1.45429737593058 | 6.27946013725745 | 0.464586574640009 | 16.348241357762 | 1.03815986110917 | 1.00387651585463 | 0.110678649538556 | 3.22458017552286 | 0.326684625160612 | 2.50234368537851 | 0.610788713910195 |
| 36.184433852219 | 1.18567117754684 | 6.22676408160029 | 0.540589824709967 | 17.6082264223384 | 0.893006297543116 | 1.03971007927414 | 0.115200737779192 | 3.42751918739264 | 0.274755539634105 | 2.440963864 | 0.609919336864539 |
| 35.8558333051159 | 1.32236081884588 | 6.94983760607035 | 0.27367217009095 | 17.9481187149551 | 1.03255961586085 | 1.16538743311248 | 0.0922910685542541 | 3.45022059328283 | 0.442809184517357 | 2.14640011986783 | 0.399977148026012 |
| 37.3313382643014 | 1.59400020776501 | 5.88258020785685 | 0.532209021188413 | 16.6558263186457 | 1.22217090862243 | 0.661915945419201 | 0.0994427342325818 | 2.58645994706501 | 0.209061266363747 | 3.4896092942048 | 0.530672705743118 |
| 37.2222324296131 | 0.886906679466114 | 5.89426389279606 | 0.654063063859931 | 17.1206299942943 | 0.809262752915021 | 0.777054445849976 | 0.0911104147879867 | 2.85032692470232 | 0.291785914916055 | 3.16024353015235 | 0.506884123077107 |
| 37.0076419030349 | 1.20591664478436 | 6.25099202787258 | 0.409093321685019 | 17.0751498382366 | 0.502093410222889 | 0.92104476421888 | 0.119328562455975 | 3.08344032706252 | 0.439359077220187 | 2.83719883971444 | 0.426059890554357 |
| 36.1472536798559 | 1.3284742141032 | 6.41338970964895 | 0.520334002009258 | 17.3811155018318 | 1.13199001118934 | 1.04113839380957 | 0.0637194445317397 | 3.26635789041152 | 0.120344696892104 | 2.64778546564983 | 0.370946533505664 |
| 35.2701597489993 | 2.32836699077225 | 6.66570185271154 | 0.784290953653764 | 18.3365008080906 | 1.17024421473915 | 1.17462714792965 | 0.100991976275424 | 3.43344926050026 | 0.290886556600151 | 2.14967593034247 | 0.700427721713673 |
| 35.5035176667564 | 0.774507788505781 | 6.85230251278198 | 0.540499541877142 | 18.2245055021515 | 1.09426120574107 | 1.24549840961559 | 0.079292408643941 | 3.73993781750304 | 0.251852894420733 | 1.99711811601582 | 0.39544921990179 |
| 36.5406513417181 | 1.40868165928913 | 6.02900909958547 | 0.572797327189195 | 17.1211781884068 | 1.24457994417053 | 0.747949446937227 | 0.0615932038882898 | 2.74501482425638 | 0.371024036197749 | 3.31273142424866 | 0.371084980337273 |
| 36.7286419872012 | 1.30708177086961 | 6.10367231827426 | 0.46482064107412 | 16.7588045892659 | 0.580471374157143 | 0.838094303895708 | 0.0893241326349171 | 2.74327154971308 | 0.404753563735986 | 2.76238708163498 | 0.499336789853715 |
| 35.9690989859161 | 1.78073176011011 | 6.26083046713479 | 0.448286836548791 | 17.9969767496304 | 1.10746015214175 | 0.970313049237376 | 0.108945269940613 | 3.12240237110825 | 0.237065824058328 | 2.79044773961236 | 0.607011878345112 |
| 36.2411558352987 | 1.46357895269139 | 6.48782353794434 | 0.370096704190957 | 17.7411310924688 | 1.19599284326256 | 1.02914304464022 | 0.0859948833032969 | 3.28651283786207 | 0.259059496960288 | 2.42965495781675 | 0.393212314754859 |
| 34.6161842790036 | 0.925942391151532 | 6.73136922525594 | 0.684328520538305 | 18.7960778651969 | 1.04524446525539 | 1.24274114218526 | 0.081119391084667 | 3.47226549023356 | 0.276562733055901 | 2.23105460141092 | 0.588917894158612 |
| 35.4240709834816 | 1.47906054554794 | 6.89977615808514 | 0.368658787610739 | 18.6406469759457 | 1.0559689048274 | 1.34817305242997 | 0.0953905381449975 | 3.45550488523039 | 0.342484818347248 | 1.62946514742945 | 0.567289956323261 |

Continuation of Table 3.9.1

| **Aftertaste_Sweetness_mean** | **Aftertaste_Sweetness_sd** | **Overall_Acceptance_mean** | **Overall_Acceptance_sd** |
| --- | --- | --- | --- |
| 2.37066111412782 | 0.346030311931646 | 3.17972630306856 | 0.466040802386532 |
| 2.15761938442809 | 0.507832059656712 | 3.55509676697498 | 0.648272436266246 |
| 2.35246047352261 | 0.354343516841456 | 3.67553278678899 | 0.717491597513223 |
| 2.49600884176396 | 0.586050718109583 | 4.01778432438126 | 0.550727337748748 |
| 2.55517448057066 | 0.325651484118415 | 4.52435676407766 | 0.383686688327355 |
| 2.78801561587027 | 0.348741683952062 | 4.68714029773146 | 0.408634308399634 |
| 2.0812379788652 | 0.344533197111372 | 3.24450449333542 | 0.660451028882645 |
| 2.36172555662006 | 0.334185076042389 | 3.5942421813729 | 0.512405022019269 |
| 2.47863037869846 | 0.371276881795087 | 4.18447156928882 | 0.552718945157196 |
| 2.66868454738626 | 0.47010923983636 | 4.41896900133724 | 0.435077914691106 |
| 2.8660120818976 | 0.2224943121837 | 4.56176544980516 | 0.57939789786934 |
| 2.77258667465422 | 0.342615162535823 | 4.72379862796302 | 0.35317428329079 |
| 2.49131959643761 | 0.354475227216191 | 3.40232114283732 | 0.701413558811657 |
| 2.51817869555018 | 0.533567135139127 | 3.68179098185404 | 0.207643385203632 |
| 2.60282116115841 | 0.378785164786707 | 4.12405301225821 | 0.611923665140695 |
| 2.71795161319633 | 0.396732784922338 | 4.27351980058343 | 0.48914939800903 |
| 2.65297126097248 | 0.415657625386783 | 4.39149995588396 | 0.427275295090461 |
| 2.90228074622385 | 0.520087910213958 | 4.65809295660622 | 0.524395350752711 |
| 2.68529034117435 | 0.397384277879373 | 3.71920739264841 | 0.732859617948168 |
| 2.43228685170849 | 0.313014544469196 | 3.89299291453768 | 0.607239584867464 |
| 2.68236593262472 | 0.450439807854712 | 4.2394025979269 | 0.379936234976647 |
| 2.57504901966406 | 0.427233558541336 | 4.63121093326427 | 0.293761168643851 |
| 2.62329749819114 | 0.439120976849756 | 4.61477978735305 | 0.455248156746685 |
| 2.83671687487843 | 0.484625339250705 | 4.94002473596766 | 0.124134285315485 |

**Supplementary Table 3.9.2**

*Pearson correlation matrix between key sonochemical parameters, physicochemical traits, and sensory evaluation scores.*

This table presents the pairwise Spearman correlation coefficients among selected key variables from the integrated dataset. The matrix includes sonochemical parameters (Power Density, Processing Time), crucial physicochemical traits (Total Catechins, Total Amino Acids, L* a* b* values), and all measured sensory scores (Umami-Sweet Ratio, Mellow Smoothness, Astringency, Aftertaste Sweetness, Overall Acceptance). Correlations were calculated using data from all samples (n=240). High absolute correlation values (e.g., |*r*| > 0.5) indicate strong monotonic relationships. This analysis quantifies the interrelationships, revealing significant positive correlations between acoustic power density and amino acid content, umami-sweet ratio, and overall acceptance, while inverse relationships are typically observed with astringency and catechins. It serves to statistically link processing variables to molecular changes and ultimate sensory quality.

| **Variable** | **Power** | **Processing_Time** | **Total_Catechins** | **Total_Amino_Acids** | **L_star** | **a_star** | **b_star** | **Umami_Sweet_Ratio** | **Mellow_Smoothness** | **Astringency** | **Aftertaste_Sweetness** | **Overall_Acceptance** |
| --- | --- | --- | --- | --- | --- | --- | --- | --- | --- | --- | --- | --- |
| Power | 1 | 0.949158116253157 | -0.38428935 | 0.248841340084279 | -0.270734644 | 0.240826346640146 | 0.343677955543058 | 0.231520347540583 | -0.010973548 | -0.123990362 | 0.146959477109868 | 0.183715275699983 |
| Processing_Time | 0.949158116253157 | 1 | -0.330598622 | 0.209889928644594 | -0.23473411 | 0.224516918696505 | 0.295642700582224 | 0.192586676852029 | -0.041491172 | -0.112720731 | 0.121018594072814 | 0.150972842961102 |
| Total_Catechins | -0.38428935 | -0.330598622 | 1 | -0.865176479 | 0.487722877133283 | -0.60747322 | -0.579033399 | -0.822062015 | -0.602635462 | 0.654179902033884 | -0.316461223 | -0.647610098 |
| Total_Amino_Acids | 0.248841340084279 | 0.209889928644594 | -0.865176479 | 1 | -0.465952534 | 0.634405111199847 | 0.537512532829126 | 0.893339294084966 | 0.707018351013038 | -0.697469725 | 0.348305526137606 | 0.696975700092534 |
| L_star | -0.270734644 | -0.23473411 | 0.487722877133283 | -0.465952534 | 1 | -0.394383583 | -0.387639489 | -0.476866786 | -0.359392524 | 0.317646208624768 | -0.166232053 | -0.332497933 |
| a_star | 0.240826346640146 | 0.224516918696505 | -0.60747322 | 0.634405111199847 | -0.394383583 | 1 | 0.364114672630874 | 0.600012152988767 | 0.434804423687911 | -0.420082037 | 0.216945606694561 | 0.486052080926795 |
| b_star | 0.343677955543058 | 0.295642700582224 | -0.579033399 | 0.537512532829126 | -0.387639489 | 0.364114672630874 | 1 | 0.473548471108434 | 0.405774430872548 | -0.359562569 | 0.202021745265472 | 0.331040485445709 |
| Umami_Sweet_Ratio | 0.231520347540583 | 0.192586676852029 | -0.822062015 | 0.893339294084966 | -0.476866786 | 0.600012152988767 | 0.473548471108434 | 1 | 0.642163926457056 | -0.664022957 | 0.317094046771645 | 0.653686466563347 |
| Mellow_Smoothness | -0.010973548 | -0.041491172 | -0.602635462 | 0.707018351013038 | -0.359392524 | 0.434804423687911 | 0.405774430872548 | 0.642163926457056 | 1 | -0.522189301 | 0.231754023507353 | 0.534204843454441 |
| Astringency | -0.123990362 | -0.112720731 | 0.654179902033884 | -0.697469725 | 0.317646208624768 | -0.420082037 | -0.359562569 | -0.664022957 | -0.522189301 | 1 | -0.247893246 | -0.714139922 |
| Aftertaste_Sweetness | 0.146959477109868 | 0.121018594072814 | -0.316461223 | 0.348305526137606 | -0.166232053 | 0.216945606694561 | 0.202021745265472 | 0.317094046771645 | 0.231754023507353 | -0.247893246 | 1 | 0.277501105779074 |
| Overall_Acceptance | 0.183715275699983 | 0.150972842961102 | -0.647610098 | 0.696975700092534 | -0.332497933 | 0.486052080926795 | 0.331040485445709 | 0.653686466563347 | 0.534204843454441 | -0.714139922 | 0.277501105779074 | 1 |
